# Supplementary material for: Phosphorylation of ELYS promotes its interaction with VAPB at decondensing chromosomes during mitosis
Source: EMBO Rep. 2024 Apr 11;25(5):18. doi: 10.1038/s44319-024-00125-6 (PMC11094025; doi:10.1038/s44319-024-00125-6)
Supplement: Supplementary file 1 — Appendix [file 44319_2024_125_MOESM1_ESM.pdf]

Appendix for article ‘’**Phosphorylation of ELYS promotes its interaction with VAPB at decondensing chromosomes during mitosis**’’

Appendix Figure S1 page 1

Appendix Figure S2 page 2

Appendix Figure S3 page 3

Appendix Table S1 page 4

Appendix Table S2 page 5

Appendix Figure S1: Phostag gel detecting ELYS after GST/GST-MSP pull down.

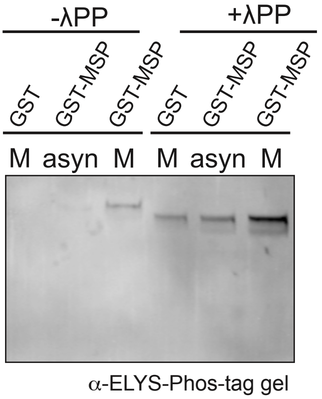

HeLa lysates from asynchronous (asyn) or mitotic (M) cells incubated with recombinant proteins (GST or GST-MSP) bound to GST-selector agarose beads. Eluted proteins were treated with (+) or without (-) λ-phosphatase (λPP) and analyzed using 6% Phos-tag gels, followed by Western blotting using antibodies against ELYS. Note the higher electrophoretic mobility of ELYS upon λPP treatment.

Appendix Figure S2: STED imaging of HeLa Flp-In-T-REx cells treated with non-targeting and VAPB siRNAs

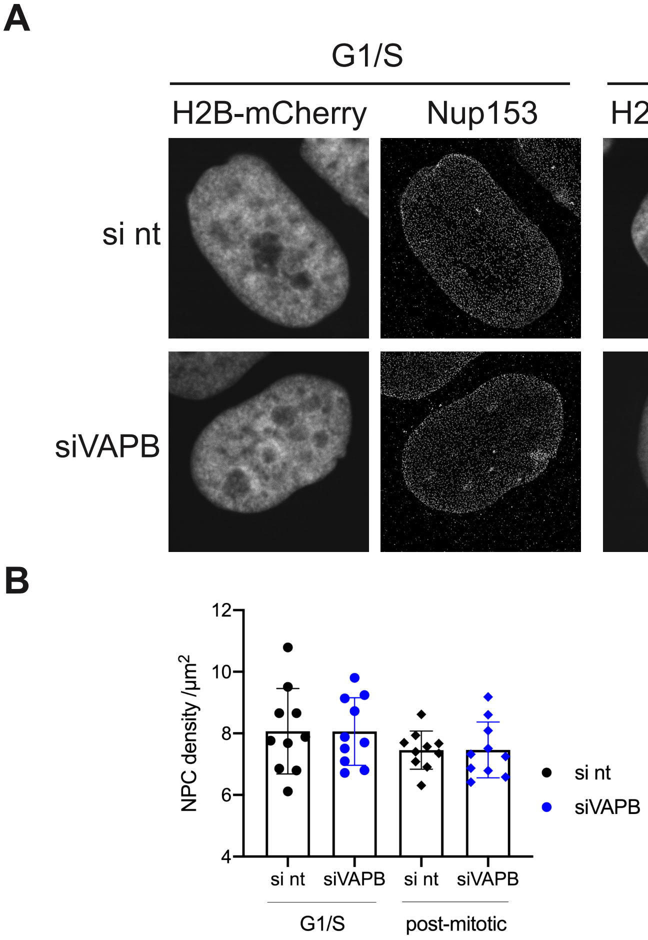

(A) HeLa Flp-In TRex cells stably expressing H2B-mCherry were treated with siRNAs against VAPB (siVAPB) or non-targeting siRNAs (si nt) and synchronized by double thymidine block (G1/S), released for 11 h (post-mitotic) before fixing and staining with antibodies against Nup153. STED imaging was performed using a laser-scanning microscope (TCS SP8, Leica) with a 100x/1.40 oil objective Scale bar, 10 µm. Images were processed using Cell Profiler (Carpenter et al., 2006). A pipeline was generated to identify the nucleus using H2B-mCherry signal and measure the number of NPCs using Nup153 signal intensities. (B) Quantification of NPC density/µm^2 as detected in A from a total of 10 technical replicates imaged per condition. The data are shown as mean ± standard deviation (n = 10).

Appendix Figure S3: Histograms showing G1, S and G2/M populations in different versions of GFPELYS1018- 1642 cells with or without tetracycline treatment

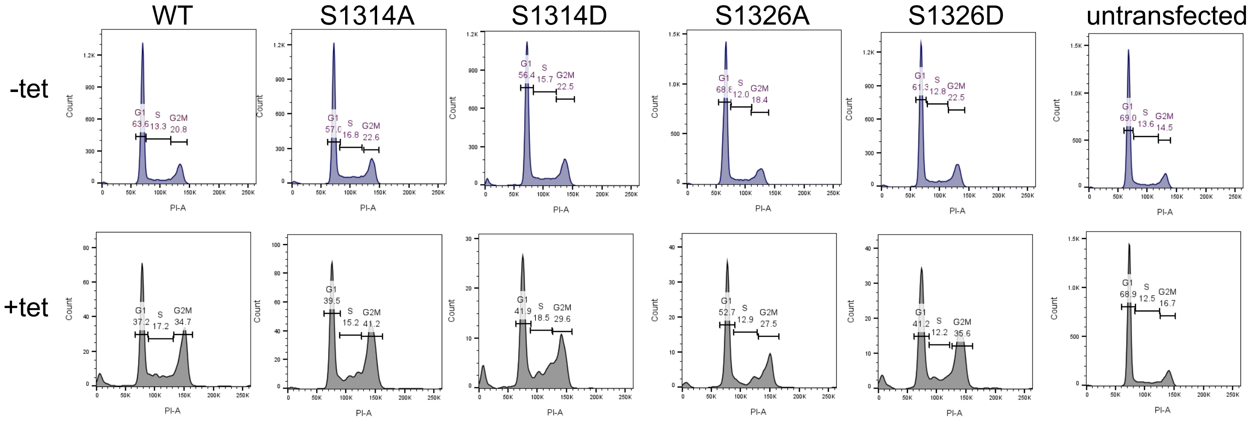

HeLa Flp In TREx stable cells expressing WT or S1314A or S1314D or S1326A or S1326D GFP-ELYS$_{1018-1642}$ or untransfected cells were treated with (+tet) or without (-tet) tetracycline for 16 h and analysed by flow cytometry using propidium iodide.

Appendix Table S1: Oligonucleotides used for cloning

**Number**	**Name**	**Sequence 5'→3'**
G1390	VAPB-fwd-KpnI	TTTGGTACCAGCGAAGGTGGAGCAGGTC
G1386	VAPB-rev-BamHI	GGATGGATCCCTACAAGGCAATCTTCCCAAT
G2387	MSP-fwd-BamHI	TTTGGATCCATGGCGAAGGTGGAG
G2388	MSP-rev-EcoRI	TCCTGAATTCCAATTCAAACACACATCTAAGTTTTG
G2403	MSP-K87D-sense	GATCCCAATGAGAAAAGTAAACACGATTTTATGGTTCAGTCTA TGTTTGCT
G2404	MSP-K87D-antisense	CTAGGGTTACTCTTTTCATTTGTGCTAAAATACCAAGTCAGAT ACAAACGA
G2405	MSP-M89D-sense	TCCCAATGAGAAAAGTAAACACGATTTTGATGTTCAGTCTATG TTTGCTCCAACTG
G2406	MSP-M89D-antisense	AGGGTTACTCTTTTCATTTGTGCTAAAACTACAAGTCAGATAC AAACGAGGTTGAC
G2594	GFP-fwd-HindIII	TTTAAGCTTGTGAGCAAGGGCGAGGAGCTG
G2595	GFP-rev-HindIII	TTTTAAGCTTCTTGTACAGCTCGTCCATGCC
G2599	HA-BamHI-sense	GATCCTACCCATACGATGTTCCAGATTACGCTGAT
G2600	HA-EcoRV-antisense	ATCAGCGTAATCTGGAACATCGTATGGGTAG
G2601	VAPB-fwd-EcoRV	TGGTGATATCATGGCGAAGGTGGAGCAGGTC
G2602	VAPB-rev-NotI	TTTGCGGCCGCCTACAAGGCAATCTTCCC
G2712	ELYS$_{1018-1642}$-fwd-BamHI	TTTGGATCCATGTATCATCTGTCAACATC
G2651	ELYS$_{1018-1642}$-rev-NotI	ATAGCGGCCGCTTAAGATGGCAAATTTGC
G2772	MSP-K43L-sense	GACAGACCGAAATGTGTGTTTTCTGGTGAAGACTACAGCACC
G2773	MSP-K43L-antisense	GGTGCTGTAGTCTTCACCAGAAAACACACATTTCGGTCTGTC
G2764	ELYS$_{1018-1642}$-fwd-EcoRI	TTTTGAATTCATGTATCATCTGTCAACATC
G2482	ELYS$_{1018-1642}$-fwd-BamHI	AAAGGATCCAGATGGCAAATTTGCAATTTG
G2781	ELYS-S1314A-sense	GTAGTCTCATCGGCTGTGATTGAAACACTGCTGTTTCCT
G2782	ELYS-S1314A-antisense	AGGAAACAGCAGTGTTTCAATCACAGCCGATGAGACTAC
G2783	ELYS-S1314D-sense	AAGGTAGTCTCATCGTCTGTGATTGAAACACTGCTGTTTCCTT TGC
G2784	ELYS-S1314D-antisense	GCAAAGGAAACAGCAGTGTTTCAATCACAGACGATGAGACTA CCTT
G2785	ELYS-S1326A-sense	CAAGGTCTTCCGGTGCCGGTGCATCCTGATA
G2786	ELYS-S1326A-antisense	TATCAGGATGCACCGGCACCGGAAGACCTTG
G2787	ELYS-S1326D-sense	GTCTCTTCAAGGTCTTCCGGGTCCGGTGCATCCTGATACTCT A
G2788	ELYS-S1326D-antisense	TAGAGTATCAGGATGCACCGGACCCGGAAGACCTTGAAGAG AC

Appendix Table S2: Antibodies used in this study

Name	Species	Origin	Application	Dilution
**Primary antibodies**				
α-ELYS	rabbit	#ab14431, Abcam	Western blotting	1:500
α-ELYS	rabbit	#HPA031658, Sigma-Aldrich	Immunofluorescence<br>PLA	1:500<br>1:750
α-emerin	mouse	#AMab90562, Sigma-Aldrich	immunofluorescence	1:500
α-GAPDH	rabbit	#10494-1-AP, Proteintech	Western blotting	1:5000
α-GFP	rat	#3H9, ChromoTek	Western blotting	1:1,000
α-HA	mouse	#HA.11 Clone 16B12, Enzo Life Sciences GmbH	Western blotting<br>Immunofluorescence<br>PLA	1:1,000<br>1:500<br>1:500
α-LBR	rabbit	#12398-1-AP, Proteintech	immunofluorescence	1:500
α-Phospho-Histone H3 (Ser28) -Alexa Fluor 488	rabbit	#53-9124-82, Thermo Fisher Scientific	flow cytometry	1:100
α-Tubulin	mouse	# 11224-1-AP, Proteintech	Western blotting	1:5000
α-VAPB	rabbit	#14477-1-AP, Proteintech	Western blotting<br>immunofluorescence	1:1,000<br>1:50
α-Cyclin E	mouse	#32-1500, Invitrogen	Western blotting	1:500
α-phospho Histone H3(Ser10)	rabbit	#9701L	Cell Signaling Technology	1:500
**Secondary antibodies**				
IRDye® 800CW α -rabbit IgG (H + L)	donkey	LI-COR Biosciences	Western blotting	1:10,000
IRDye® 800CW α-mouse IgG (H + L)	donkey	LI-COR Biosciences	Western blotting	1:10,000
α-rabbit-Alexa Fluor 488	donkey	#A-21206, Thermo Fisher Scientific	immunofluorescence	1:500
α-mouse-Alexa Fluor 488	donkey	#A-21202, Thermo Fisher Scientific	immunofluorescence	1:500
α-rabbit-Alexa Fluor 594	donkey	#A-21207, Thermo Fisher Scientific	immunofluorescence	1:500
α-mouse-Alexa Fluor 594	donkey	#A-21203, Thermo Fisher Scientific	immunofluorescence	1:500
α-rabbit-Alexa Fluor 647	donkey	#A-31573, Thermo Fisher Scientific	immunofluorescence	1:500
