# Supplementary figures and images for: Phosphorylation of ELYS promotes its interaction with VAPB at decondensing chromosomes during mitosis (part 1 of 2)
Source: EMBO Rep. 2024 Apr 11;25(5):18. doi: 10.1038/s44319-024-00125-6 (PMC11094025; doi:10.1038/s44319-024-00125-6)

Figure 1E

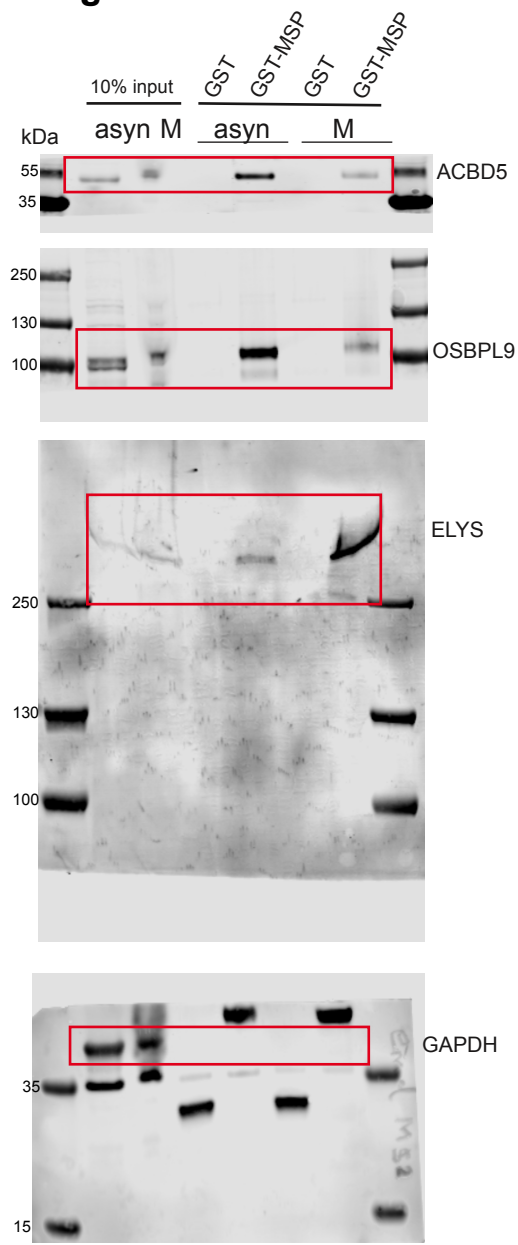

Supplement: Supplementary file 9 — Source data Fig. 1 [file 44319_2024_125_MOESM9_ESM.zip › Figure 1/Figure 1E.pdf]

Figure 1A

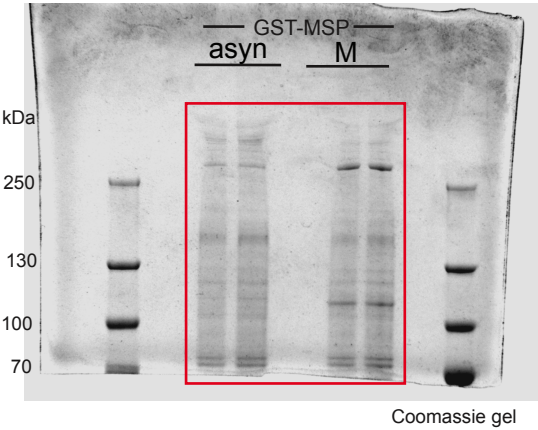

Supplement: Supplementary file 9 — Source data Fig. 1 [file 44319_2024_125_MOESM9_ESM.zip › Figure 1/Figure 1A.pdf]

Figure 2A

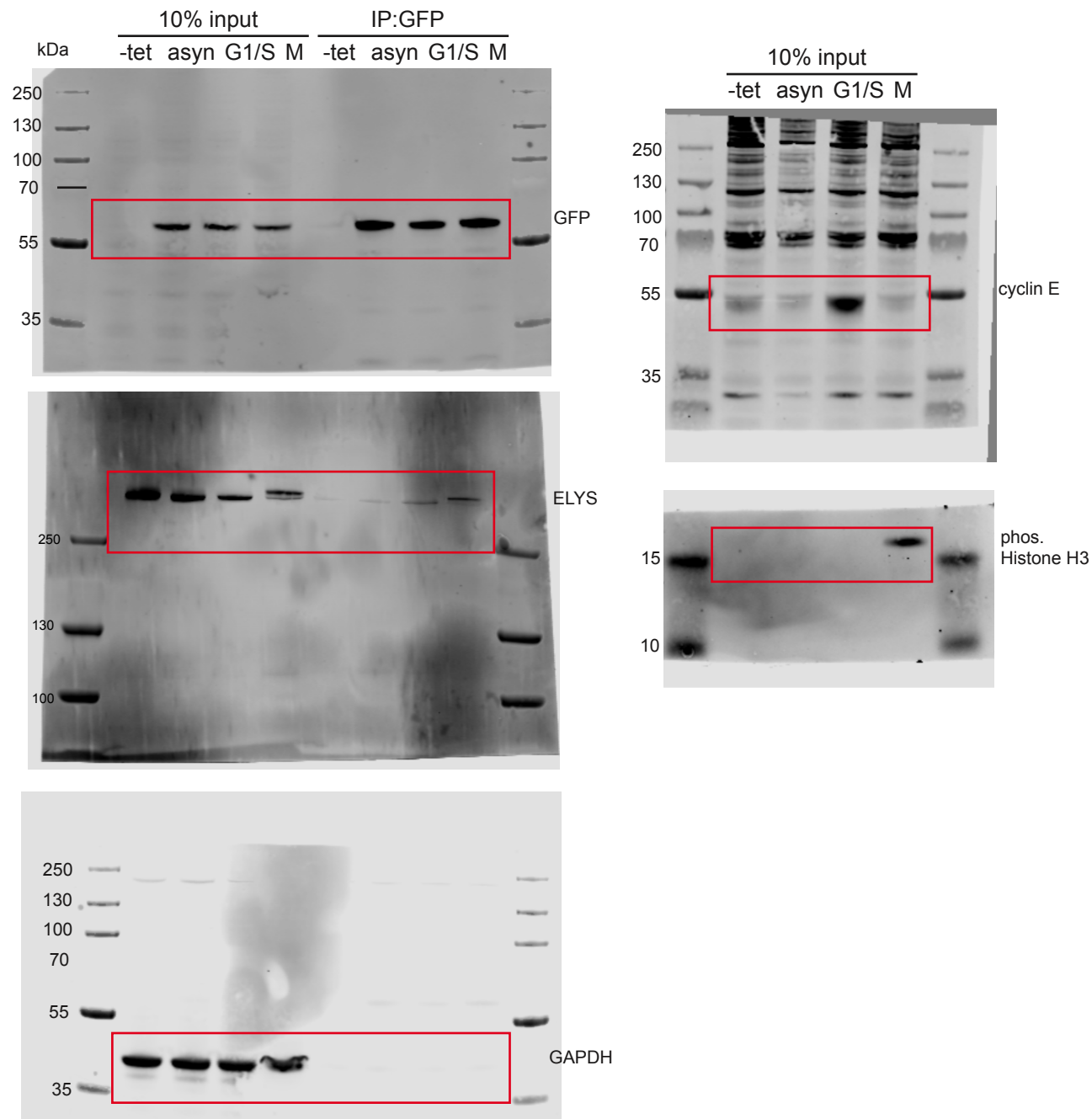

Supplement: Supplementary file 10 — Source data Fig. 2 [file 44319_2024_125_MOESM10_ESM.zip › Figure 2/Figure 2A.pdf]

Figure 2C

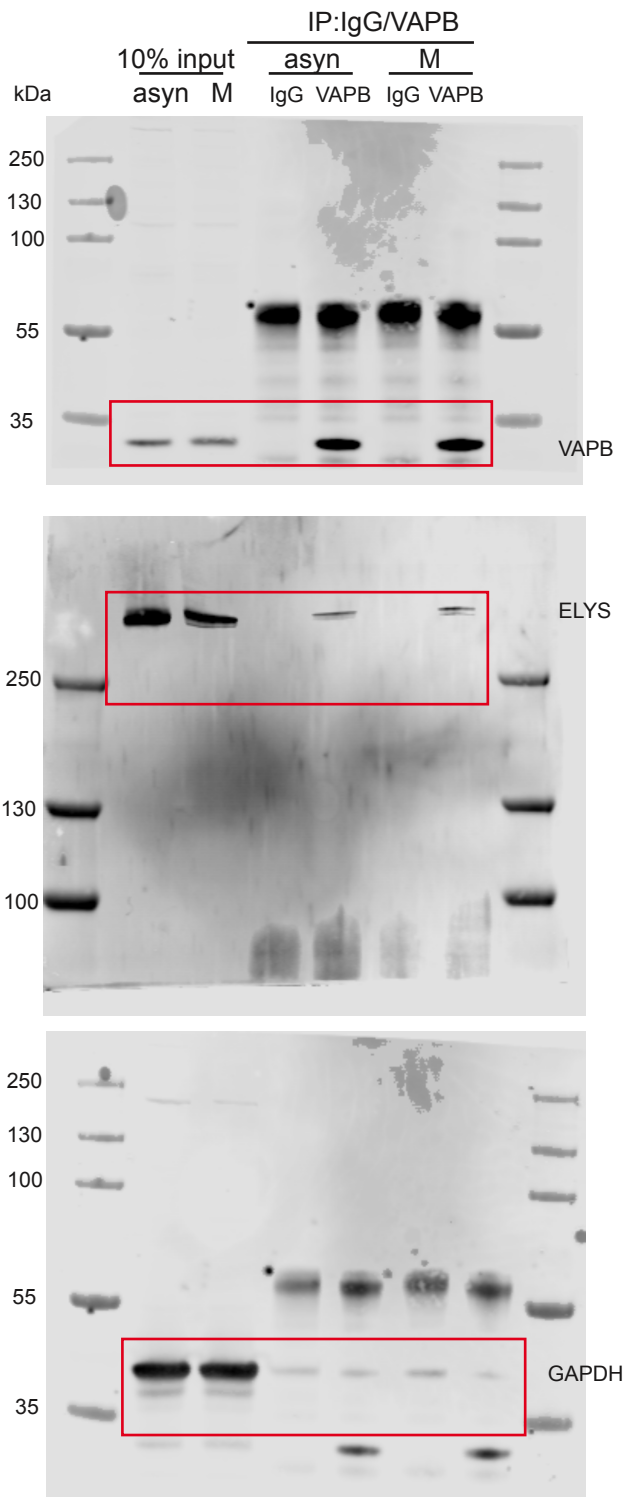

Supplement: Supplementary file 10 — Source data Fig. 2 [file 44319_2024_125_MOESM10_ESM.zip › Figure 2/Figure 2C.pdf]

Figure 2E

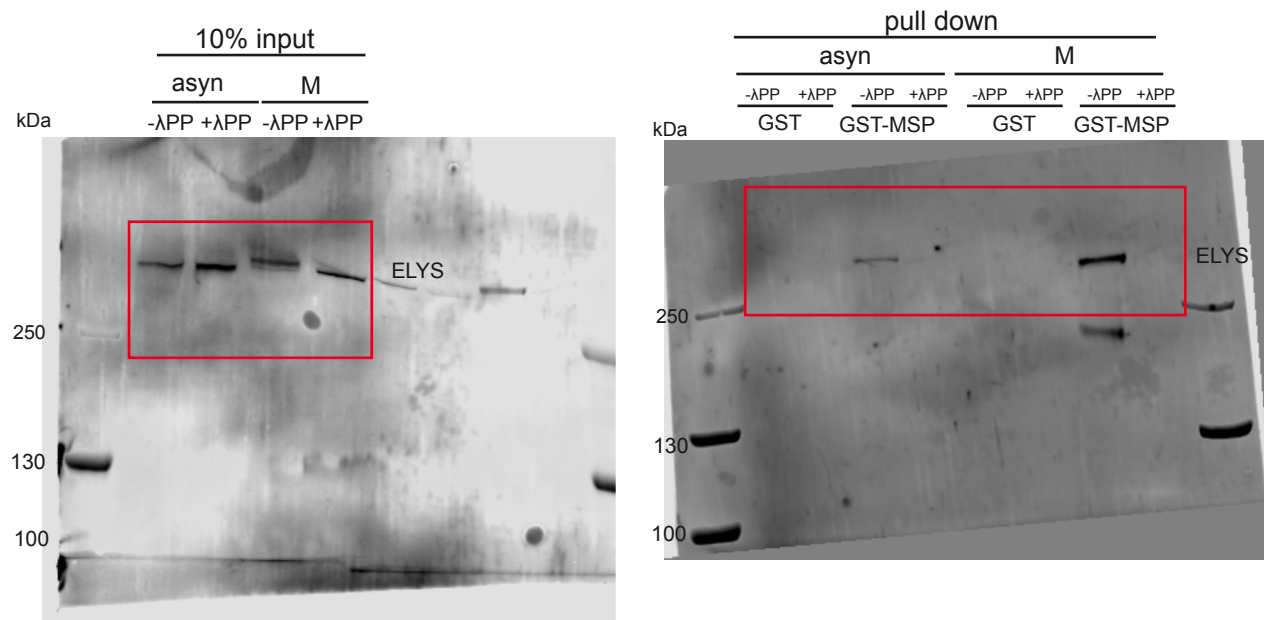

Supplement: Supplementary file 10 — Source data Fig. 2 [file 44319_2024_125_MOESM10_ESM.zip › Figure 2/Figure 2E.pdf]

Figure 2D

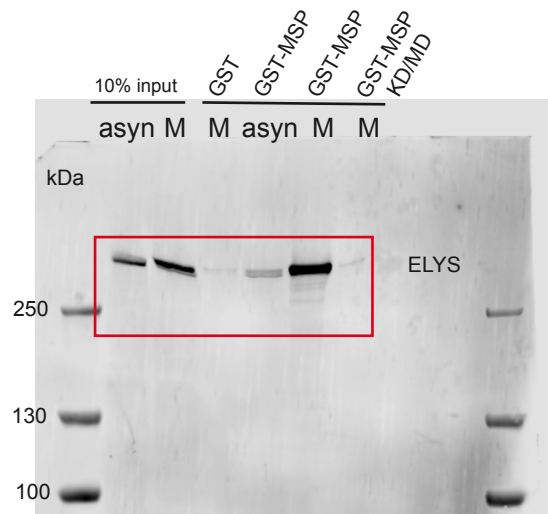

Supplement: Supplementary file 10 — Source data Fig. 2 [file 44319_2024_125_MOESM10_ESM.zip › Figure 2/Figure 2D.pdf]

Figure 3D

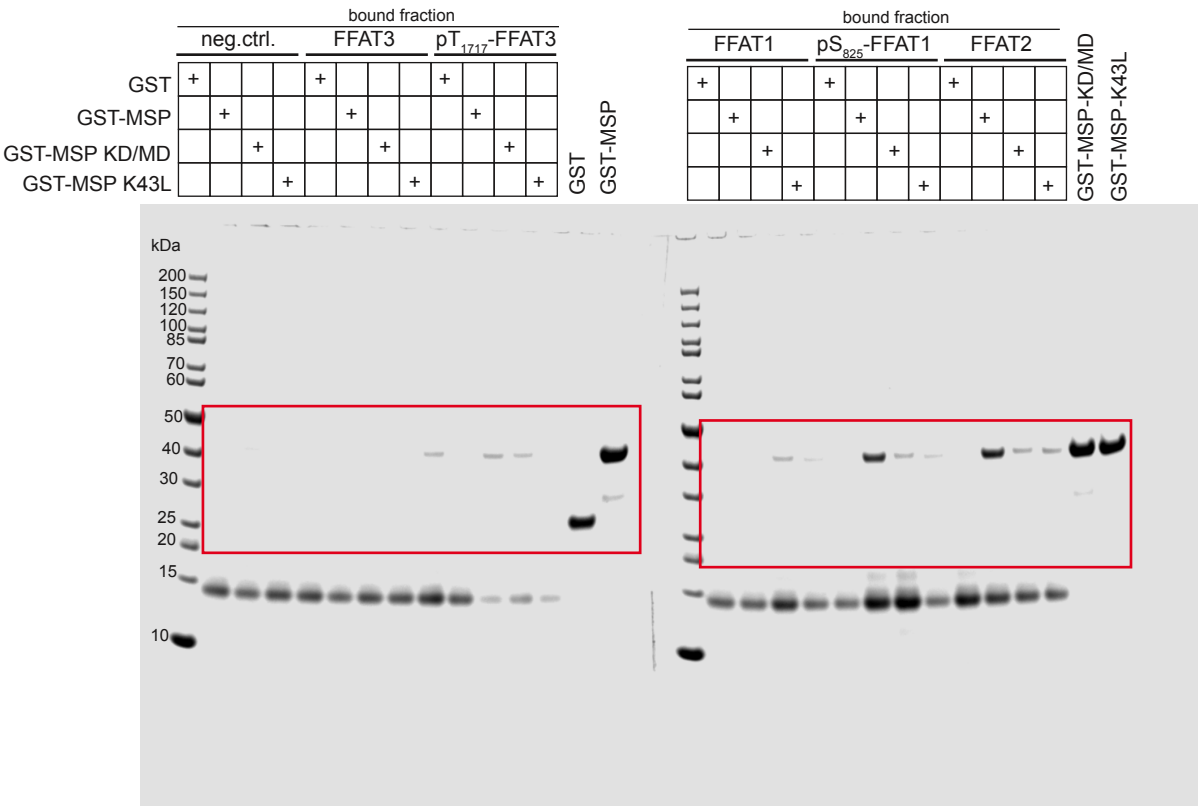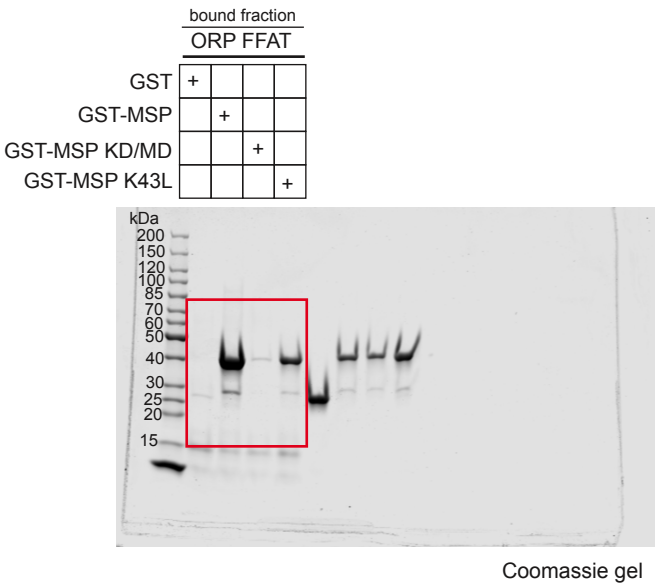

Supplement: Supplementary file 11 — Source data Fig. 3 [file 44319_2024_125_MOESM11_ESM.zip › Figure 3/Figure 3D.pdf]

Figure 3C

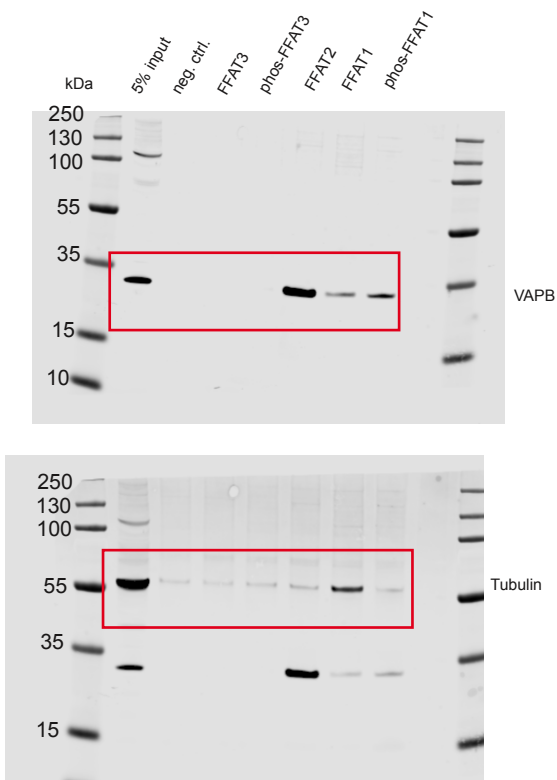

Supplement: Supplementary file 11 — Source data Fig. 3 [file 44319_2024_125_MOESM11_ESM.zip › Figure 3/Figure 3C.pdf]

Figure 4C

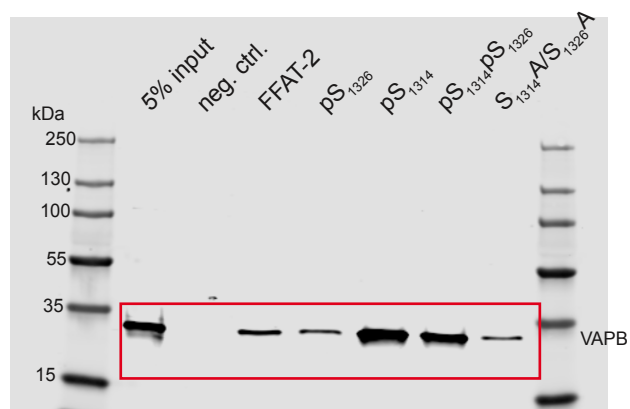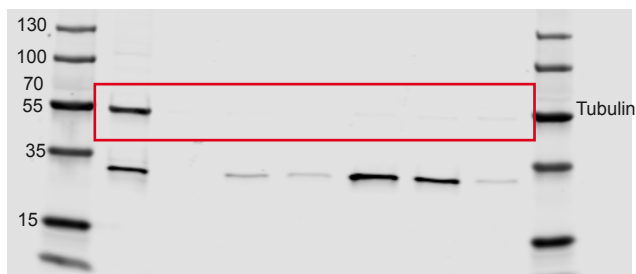

Supplement: Supplementary file 12 — Source data Fig. 4 [file 44319_2024_125_MOESM12_ESM.zip › Figure 4/Figure 4C.pdf]

Figure 4E

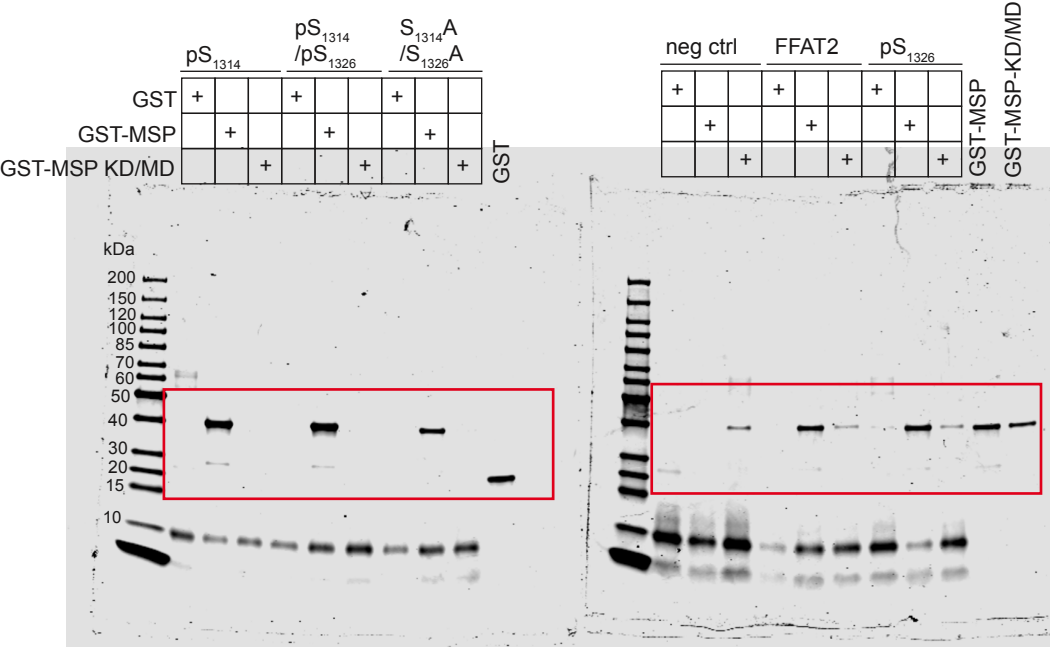

Coomassie gel

Supplement: Supplementary file 12 — Source data Fig. 4 [file 44319_2024_125_MOESM12_ESM.zip › Figure 4/Figure 4E.pdf]

Figure 5E

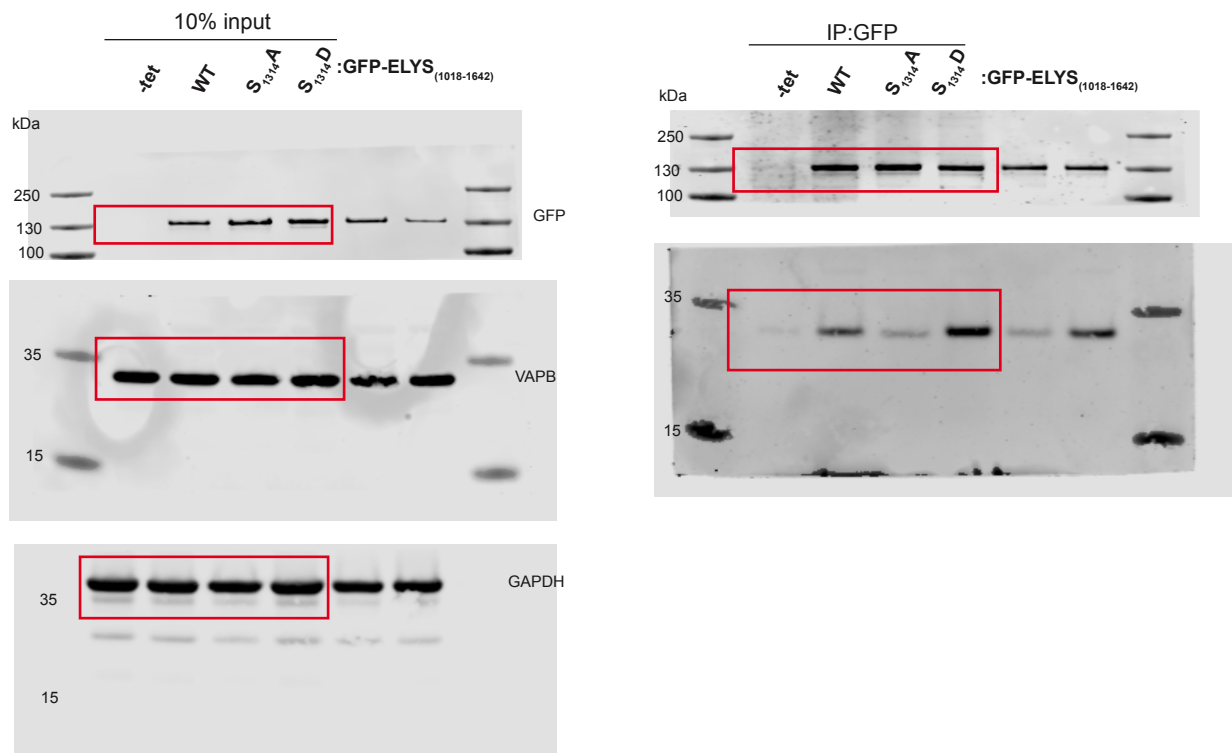

Supplement: Supplementary file 13 — Source data Fig. 5 [file 44319_2024_125_MOESM13_ESM.zip › Figure 5/Figure 5E.pdf]

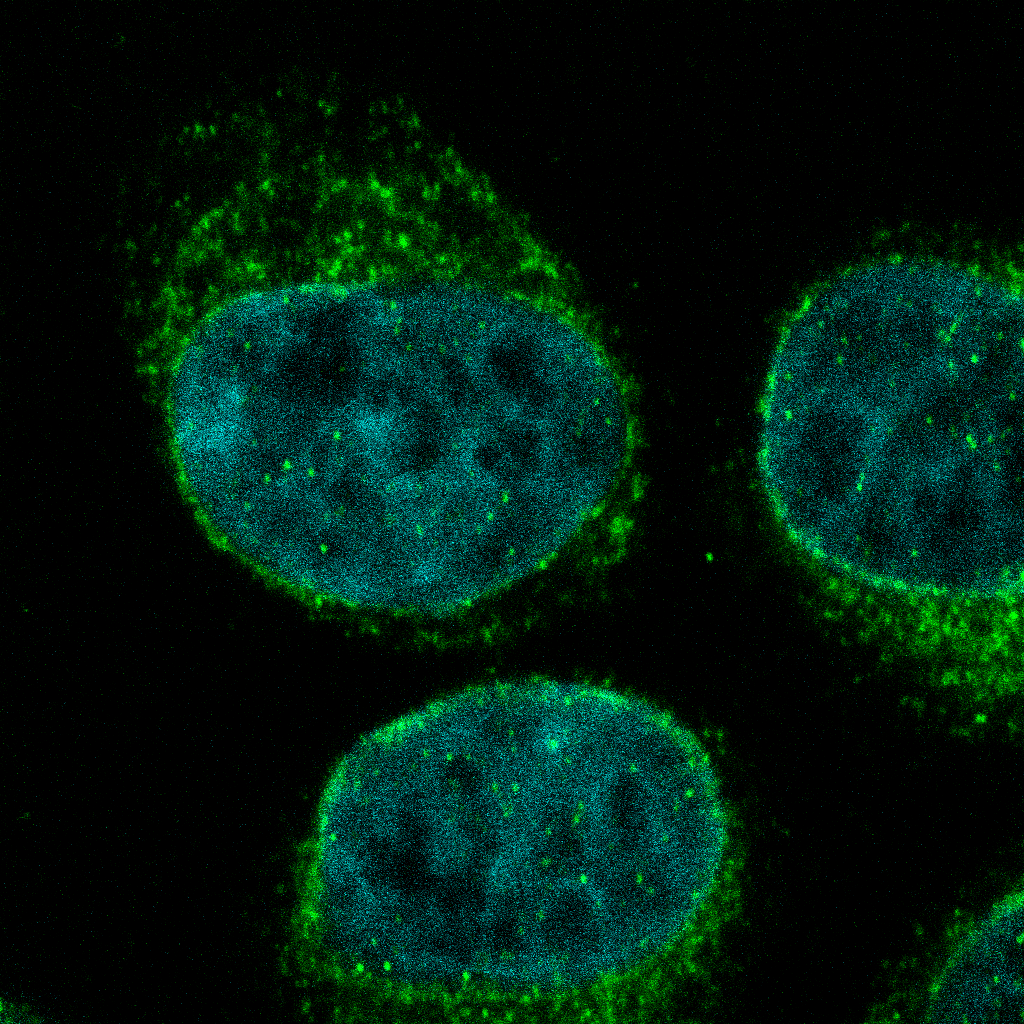

Supplement: Supplementary file 14 — Source data Fig. 6 [file 44319_2024_125_MOESM14_ESM.zip › Figure 6/Figure 6A/interphase 02_merge.tif]

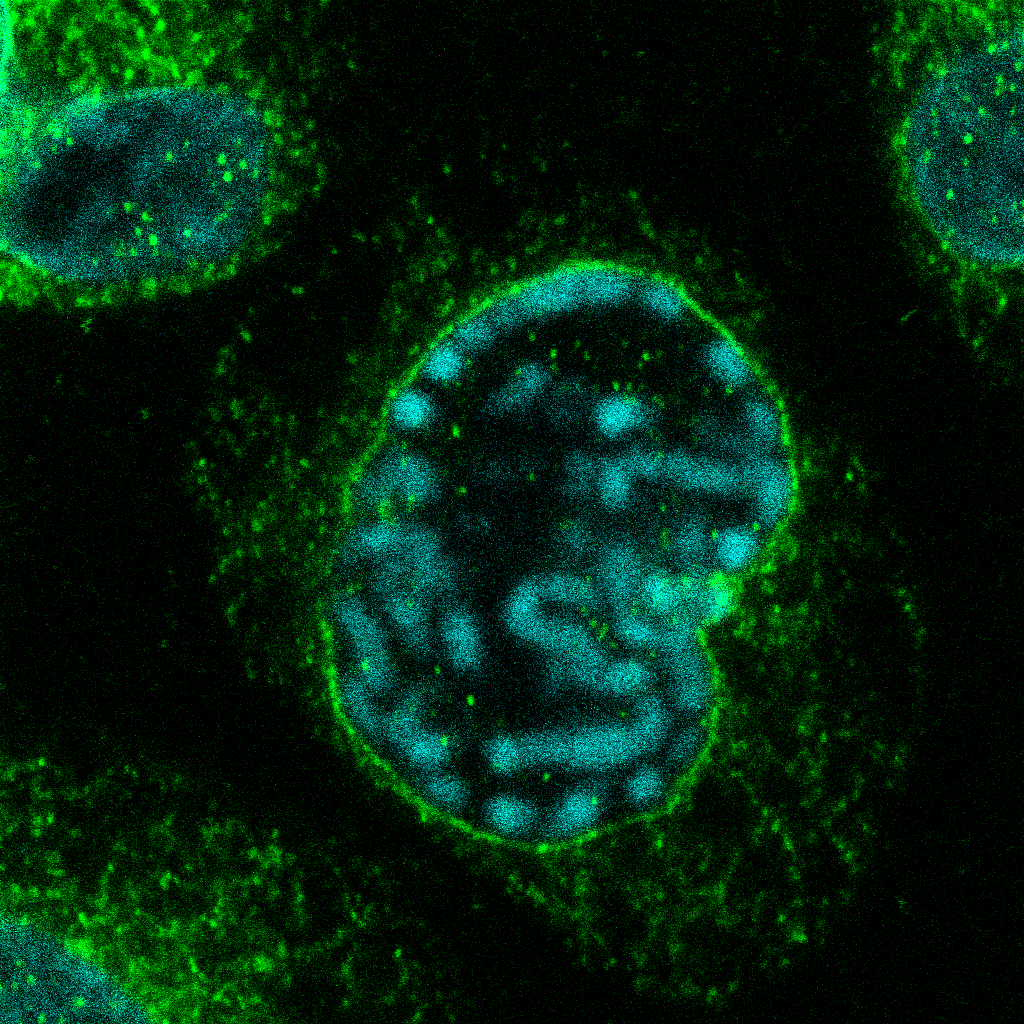

Supplement: Supplementary file 14 — Source data Fig. 6 [file 44319_2024_125_MOESM14_ESM.zip › Figure 6/Figure 6A/prophase_merge.tif]

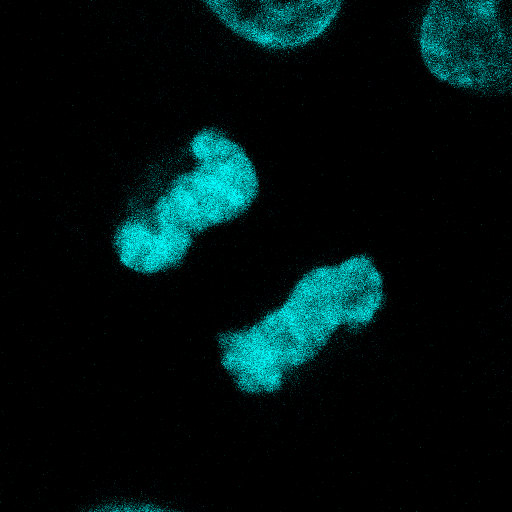

Supplement: Supplementary file 14 — Source data Fig. 6 [file 44319_2024_125_MOESM14_ESM.zip › Figure 6/Figure 6A/telophase_DAPI.tif]

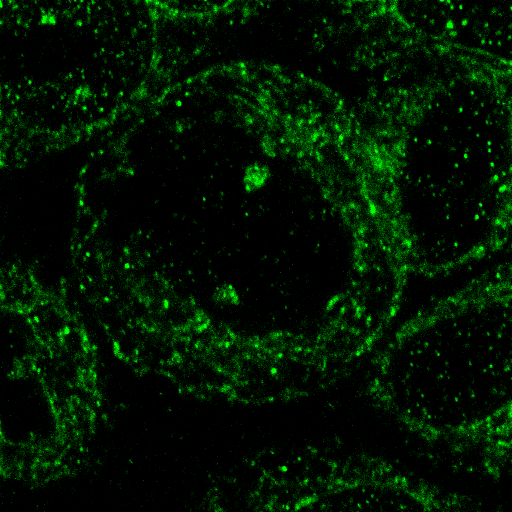

Supplement: Supplementary file 14 — Source data Fig. 6 [file 44319_2024_125_MOESM14_ESM.zip › Figure 6/Figure 6A/metaphase 02 _vapb.tif]

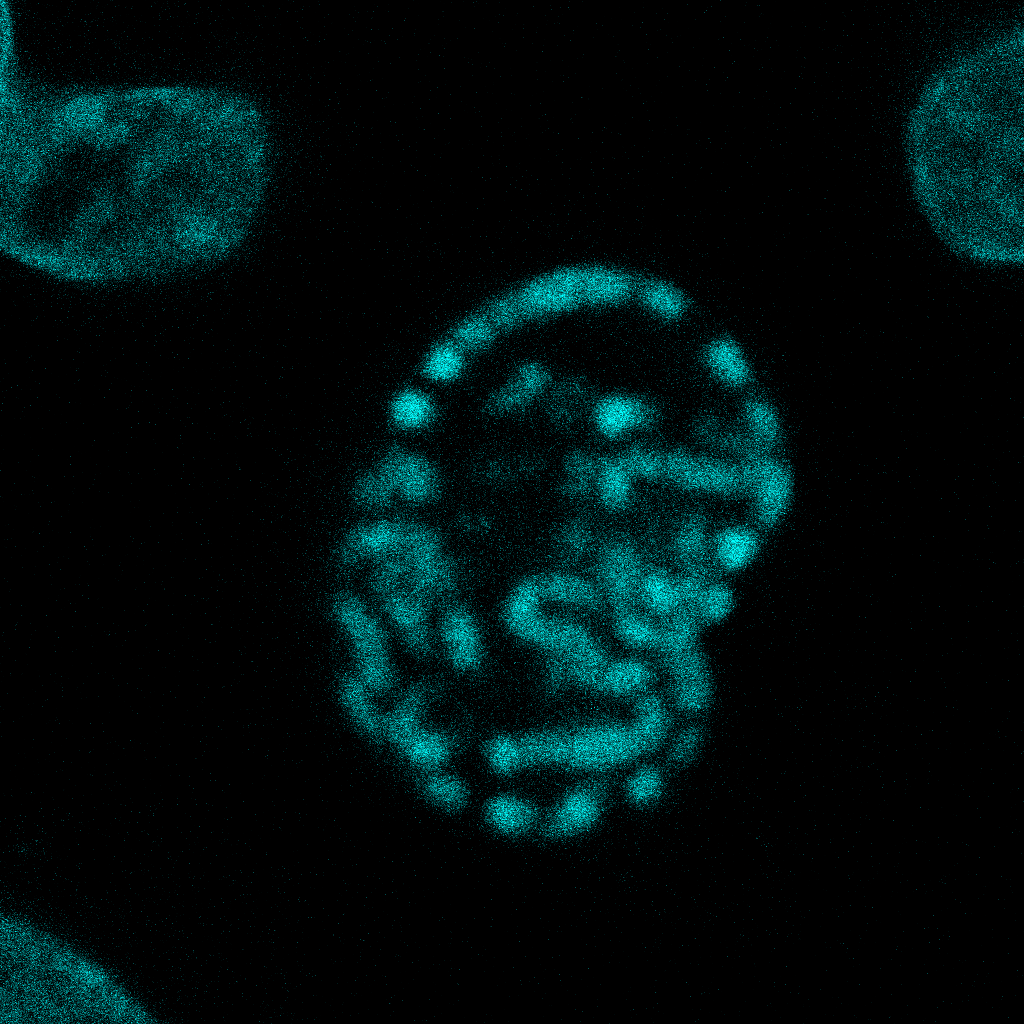

Supplement: Supplementary file 14 — Source data Fig. 6 [file 44319_2024_125_MOESM14_ESM.zip › Figure 6/Figure 6A/prophase_DAPI.tif]

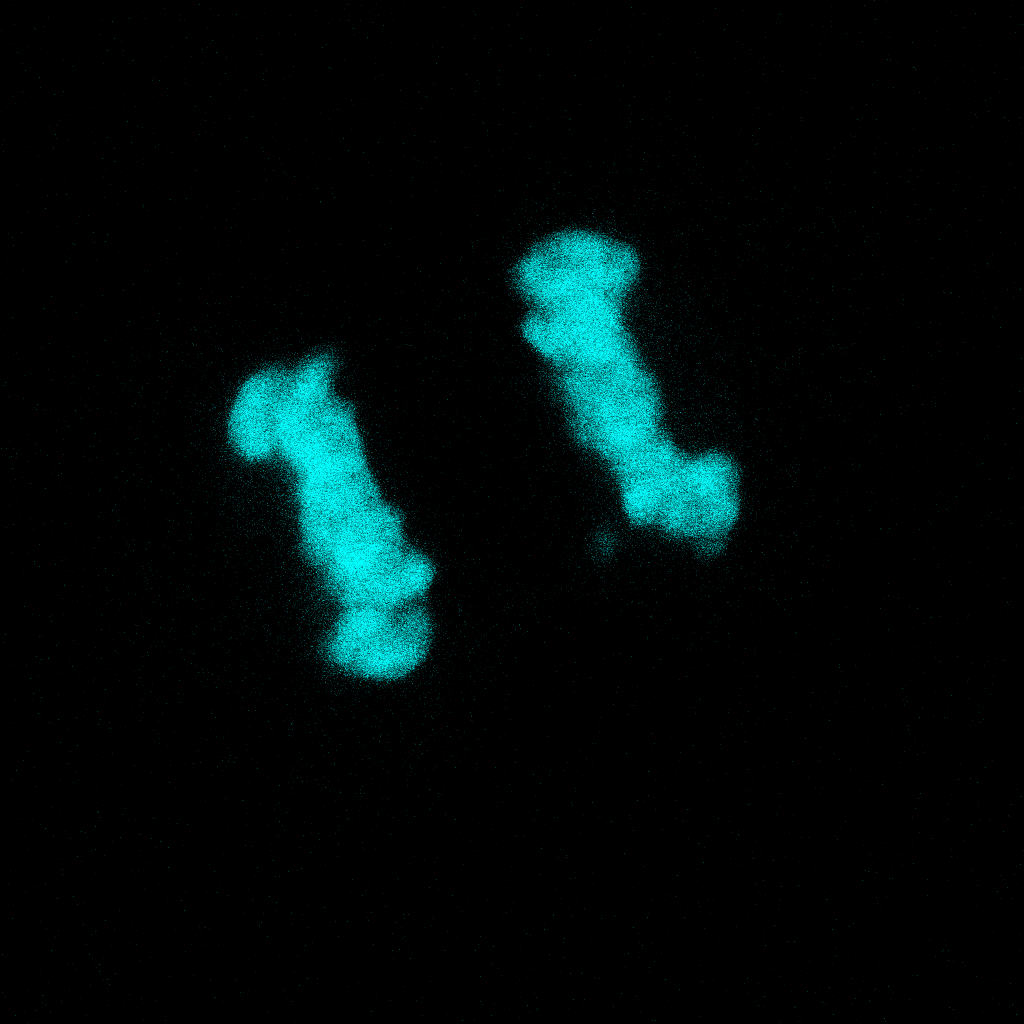

Supplement: Supplementary file 14 — Source data Fig. 6 [file 44319_2024_125_MOESM14_ESM.zip › Figure 6/Figure 6A/anaphase 02_DAPI.tif]

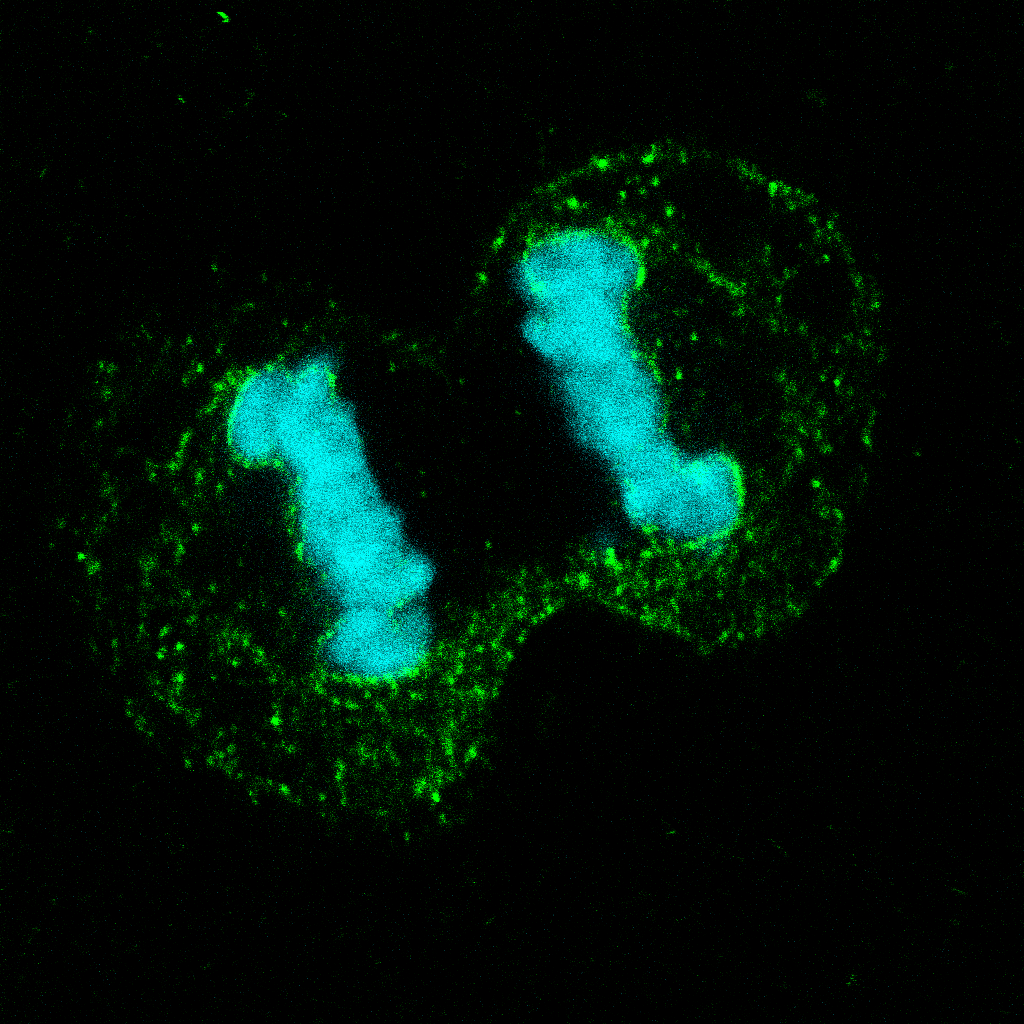

Supplement: Supplementary file 14 — Source data Fig. 6 [file 44319_2024_125_MOESM14_ESM.zip › Figure 6/Figure 6A/anaphase 02_merge.tif]

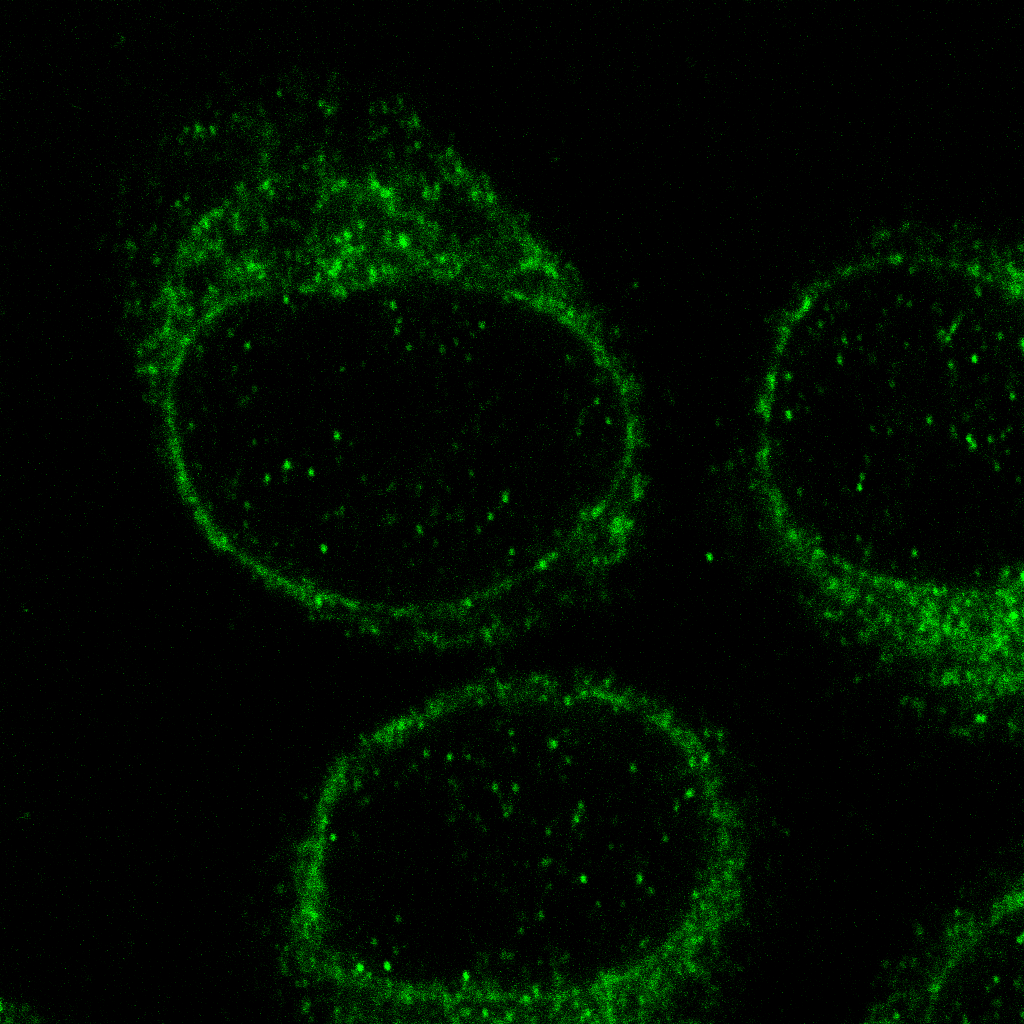

Supplement: Supplementary file 14 — Source data Fig. 6 [file 44319_2024_125_MOESM14_ESM.zip › Figure 6/Figure 6A/interphase 02_VAPB.tif]

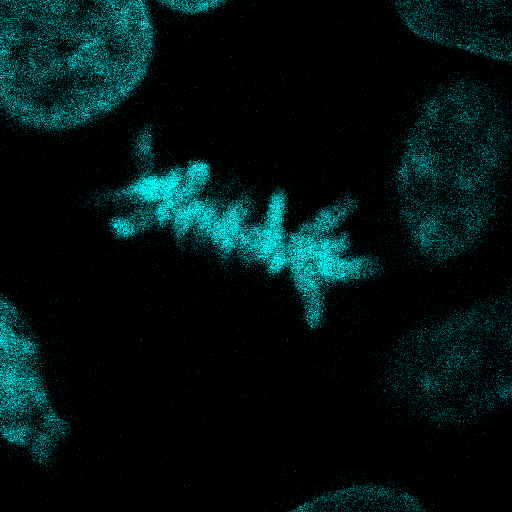

Supplement: Supplementary file 14 — Source data Fig. 6 [file 44319_2024_125_MOESM14_ESM.zip › Figure 6/Figure 6A/metaphase 02_DAPI.tif]

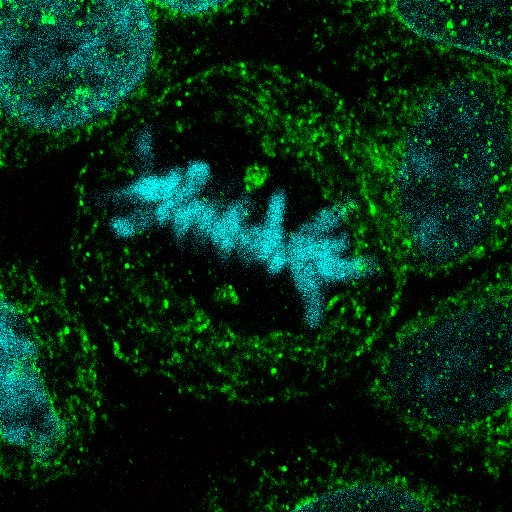

Supplement: Supplementary file 14 — Source data Fig. 6 [file 44319_2024_125_MOESM14_ESM.zip › Figure 6/Figure 6A/metaphase 02_merge.tif]

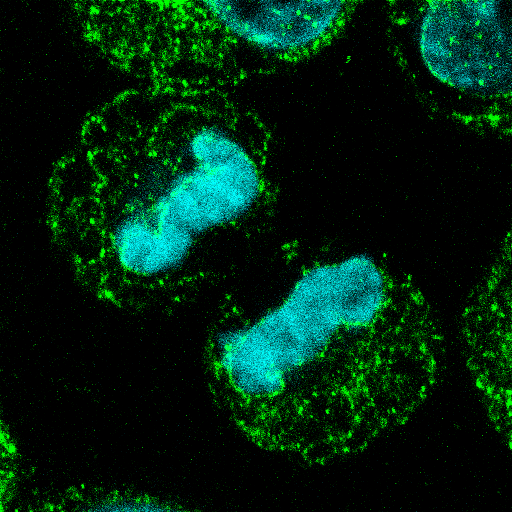

Supplement: Supplementary file 14 — Source data Fig. 6 [file 44319_2024_125_MOESM14_ESM.zip › Figure 6/Figure 6A/telophase_merge.tif]

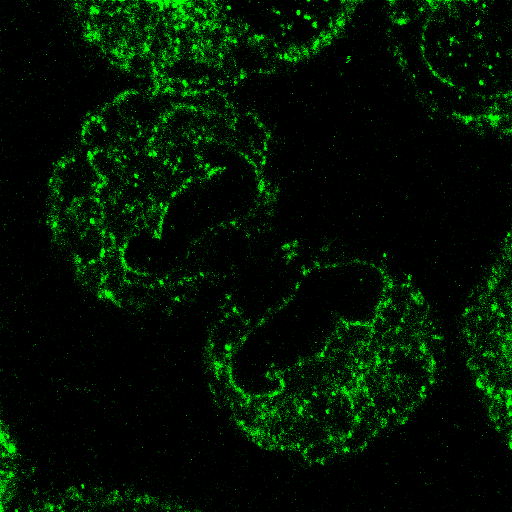

Supplement: Supplementary file 14 — Source data Fig. 6 [file 44319_2024_125_MOESM14_ESM.zip › Figure 6/Figure 6A/telophase_vapb.tif]

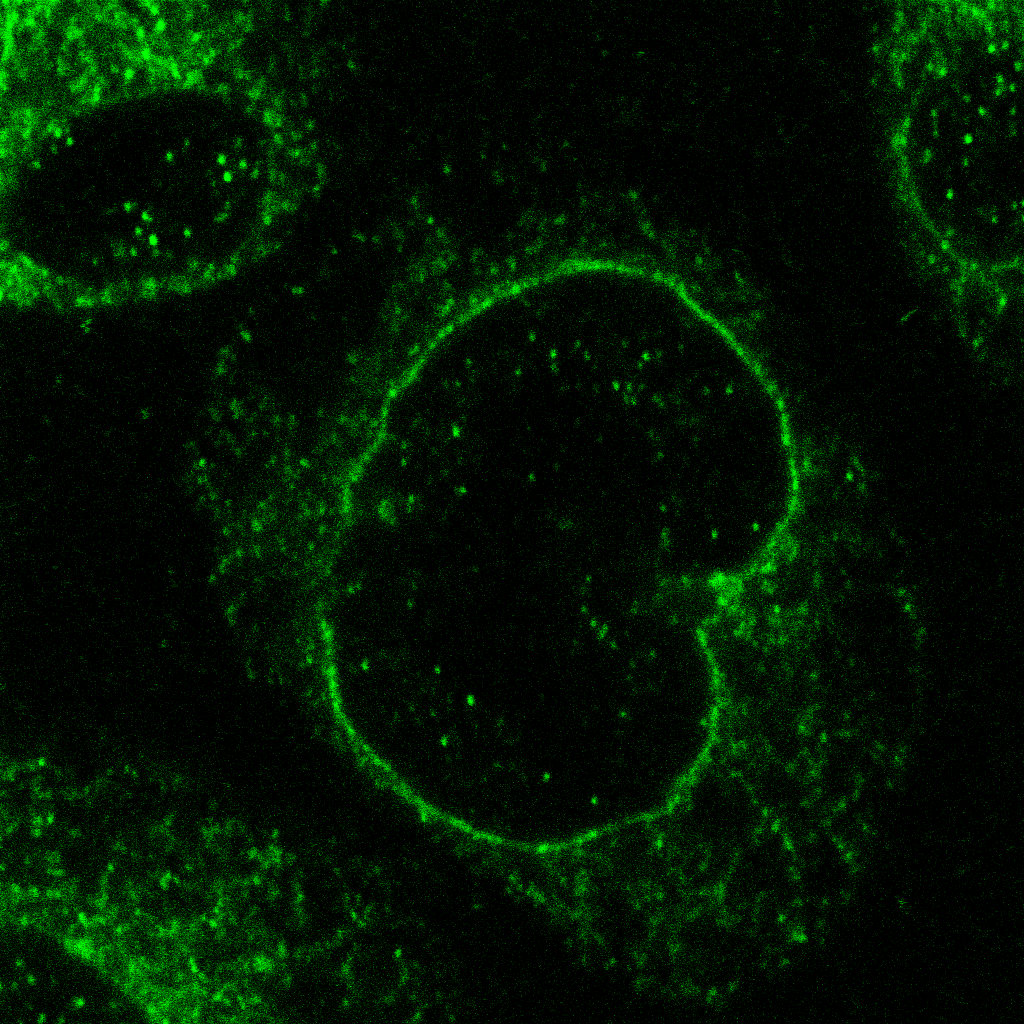

Supplement: Supplementary file 14 — Source data Fig. 6 [file 44319_2024_125_MOESM14_ESM.zip › Figure 6/Figure 6A/prophase_vapb.tif]

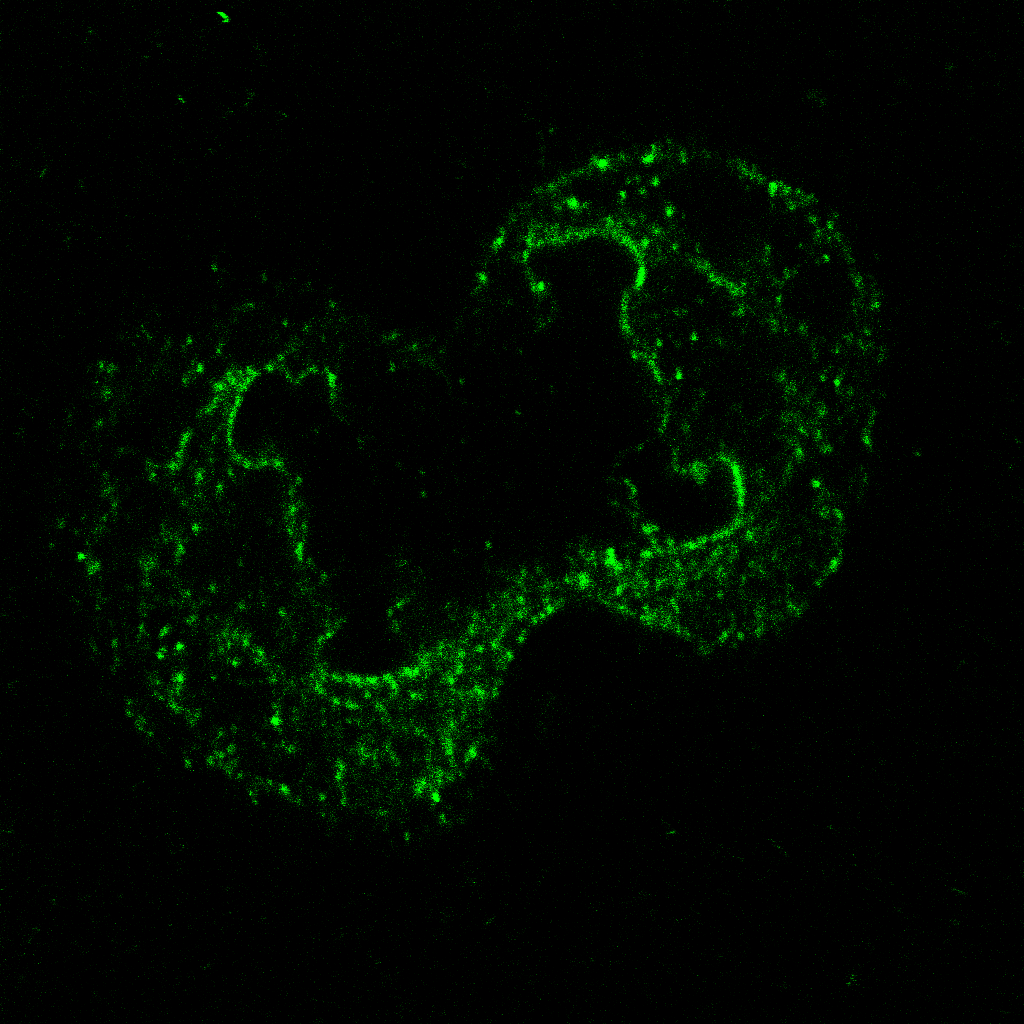

Supplement: Supplementary file 14 — Source data Fig. 6 [file 44319_2024_125_MOESM14_ESM.zip › Figure 6/Figure 6A/anaphase 02_vapb.tif]

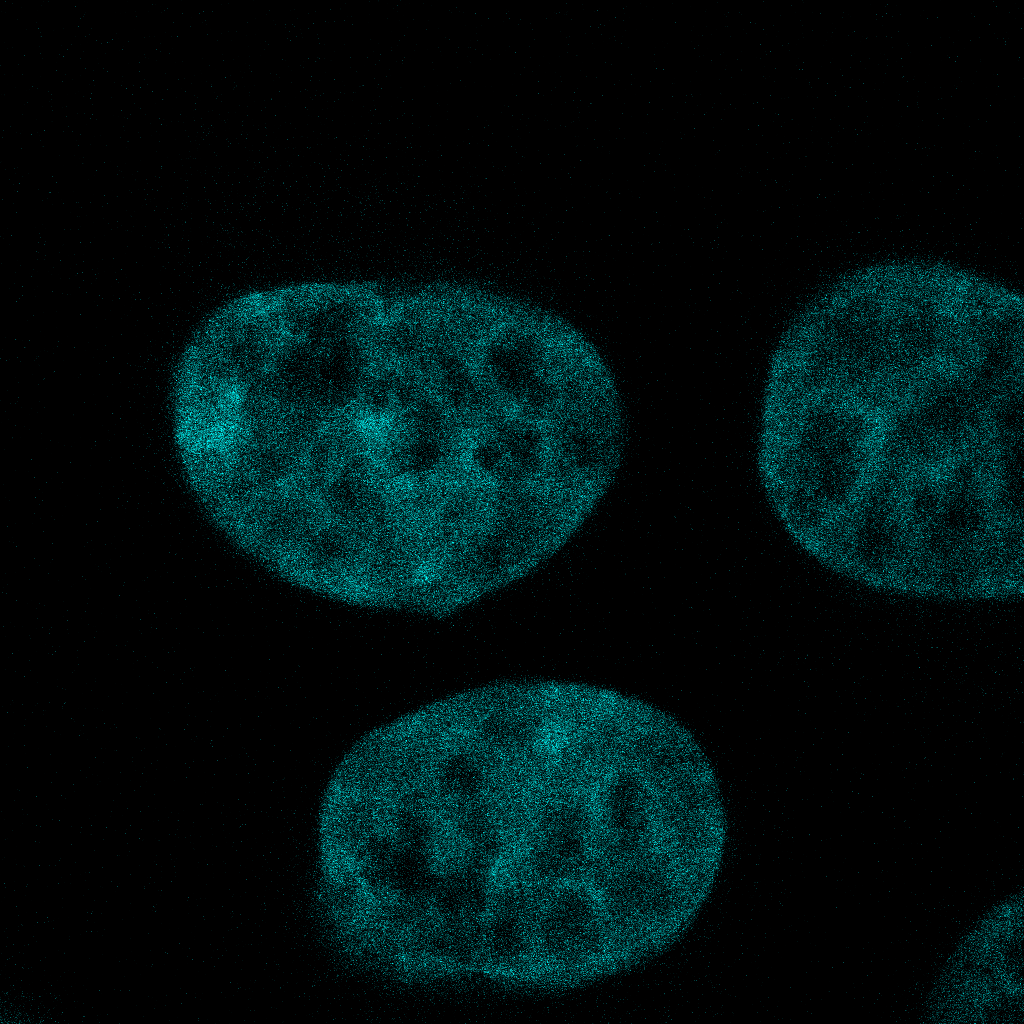

Supplement: Supplementary file 14 — Source data Fig. 6 [file 44319_2024_125_MOESM14_ESM.zip › Figure 6/Figure 6A/interphase 02_DAPI.tif]

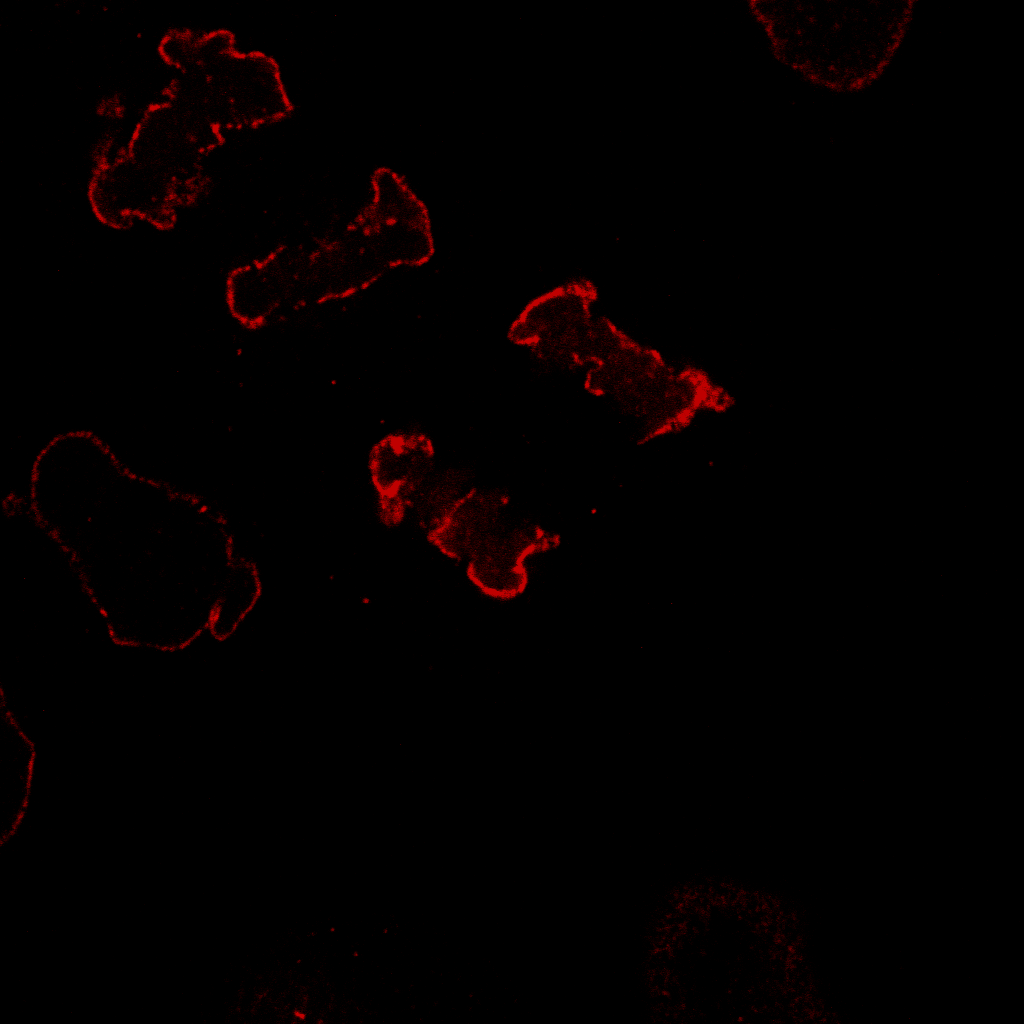

Supplement: Supplementary file 14 — Source data Fig. 6 [file 44319_2024_125_MOESM14_ESM.zip › Figure 6/Figure 6C/GFP VAPB_ELYS_ELYS.tif]

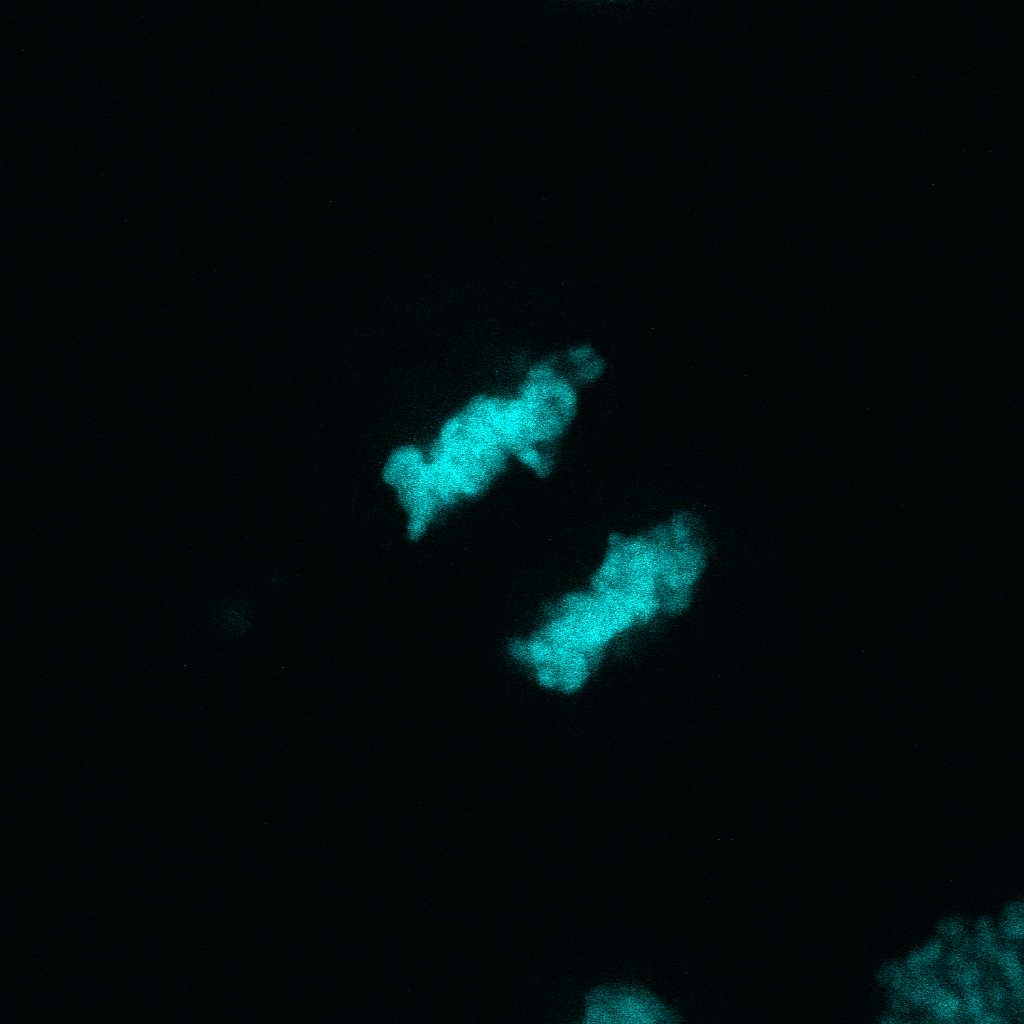

Supplement: Supplementary file 14 — Source data Fig. 6 [file 44319_2024_125_MOESM14_ESM.zip › Figure 6/Figure 6C/GFP VAPB_emerin_DAPI.tif]

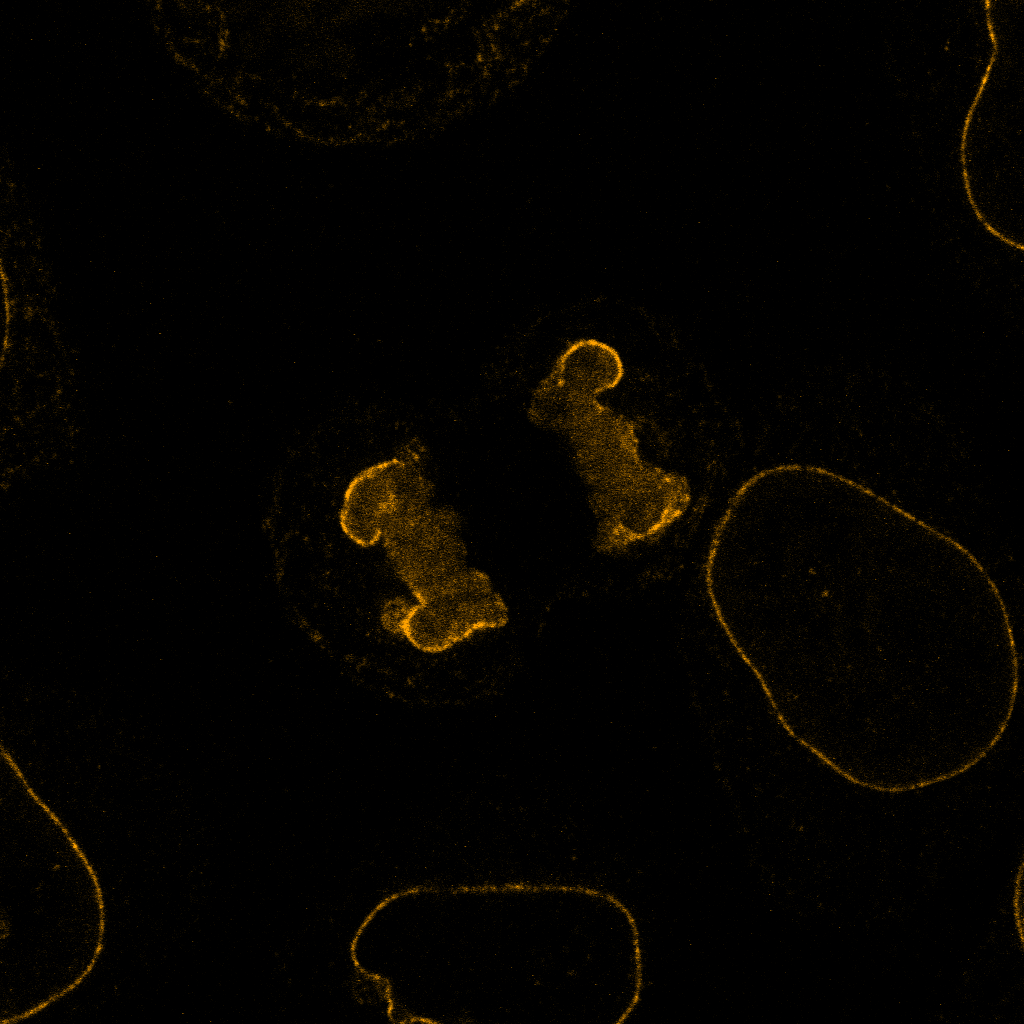

Supplement: Supplementary file 14 — Source data Fig. 6 [file 44319_2024_125_MOESM14_ESM.zip › Figure 6/Figure 6C/GFP VAPB_LBR_LBR.tif]

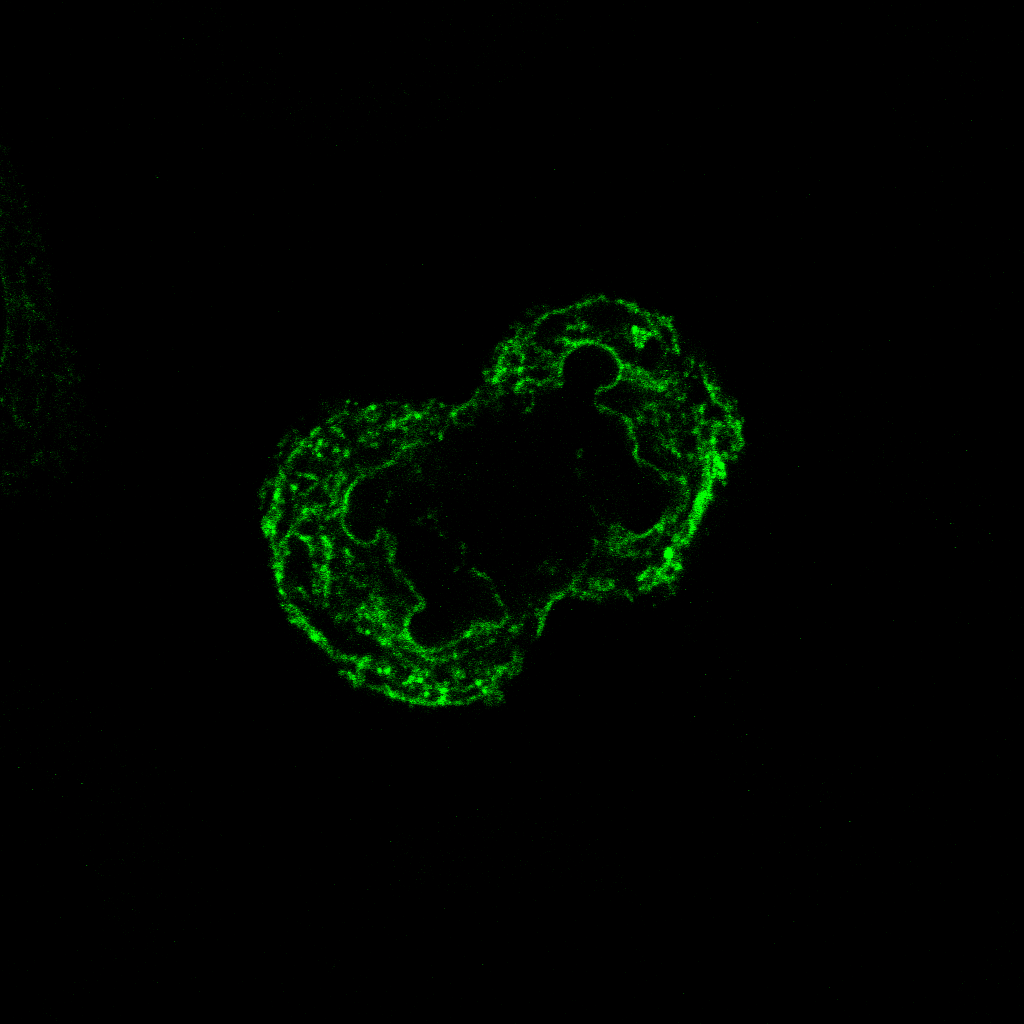

Supplement: Supplementary file 14 — Source data Fig. 6 [file 44319_2024_125_MOESM14_ESM.zip › Figure 6/Figure 6C/GFP VAPB_LBR_GFP.tif]

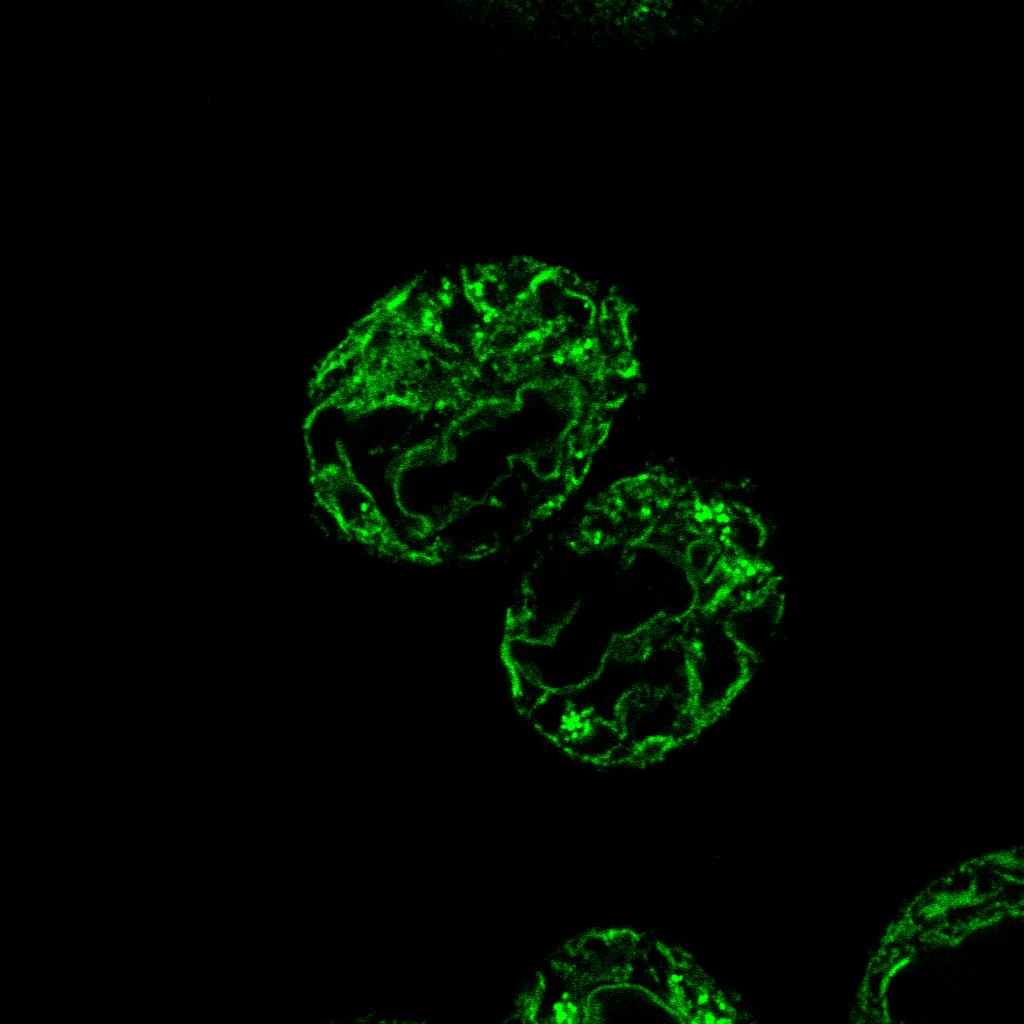

Supplement: Supplementary file 14 — Source data Fig. 6 [file 44319_2024_125_MOESM14_ESM.zip › Figure 6/Figure 6C/GFP VAPB_emerin_GFP.tif]

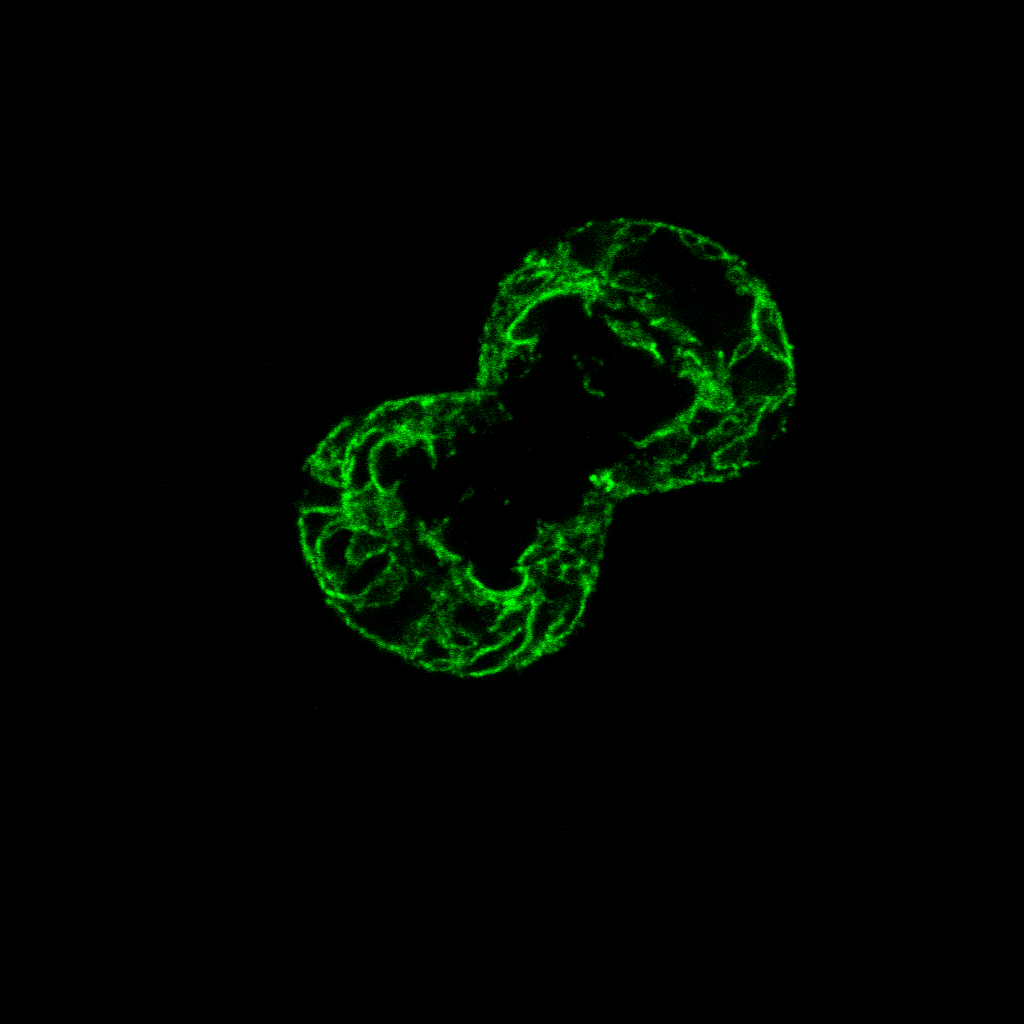

Supplement: Supplementary file 14 — Source data Fig. 6 [file 44319_2024_125_MOESM14_ESM.zip › Figure 6/Figure 6C/GFP VAPB_ELYS_GFP.tif]

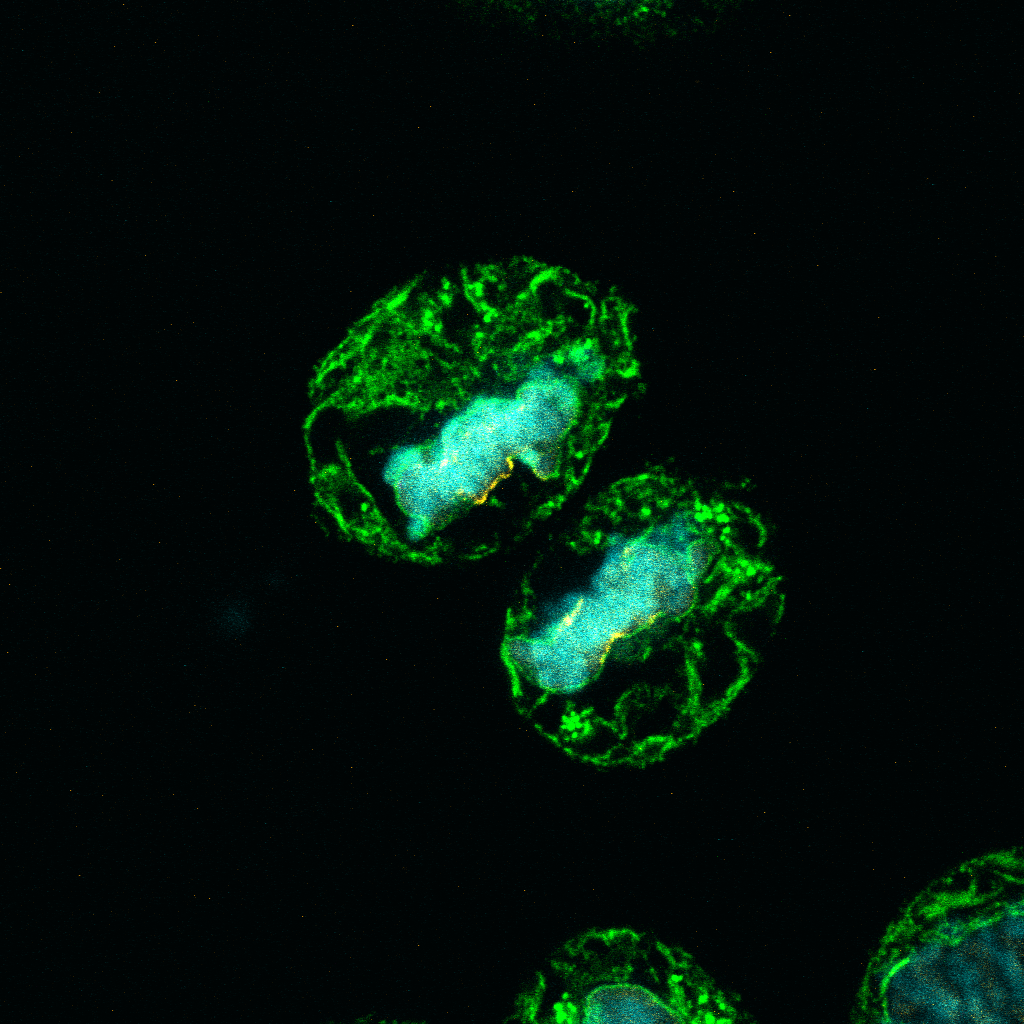

Supplement: Supplementary file 14 — Source data Fig. 6 [file 44319_2024_125_MOESM14_ESM.zip › Figure 6/Figure 6C/GFP VAPB_emerin_merge.tif]

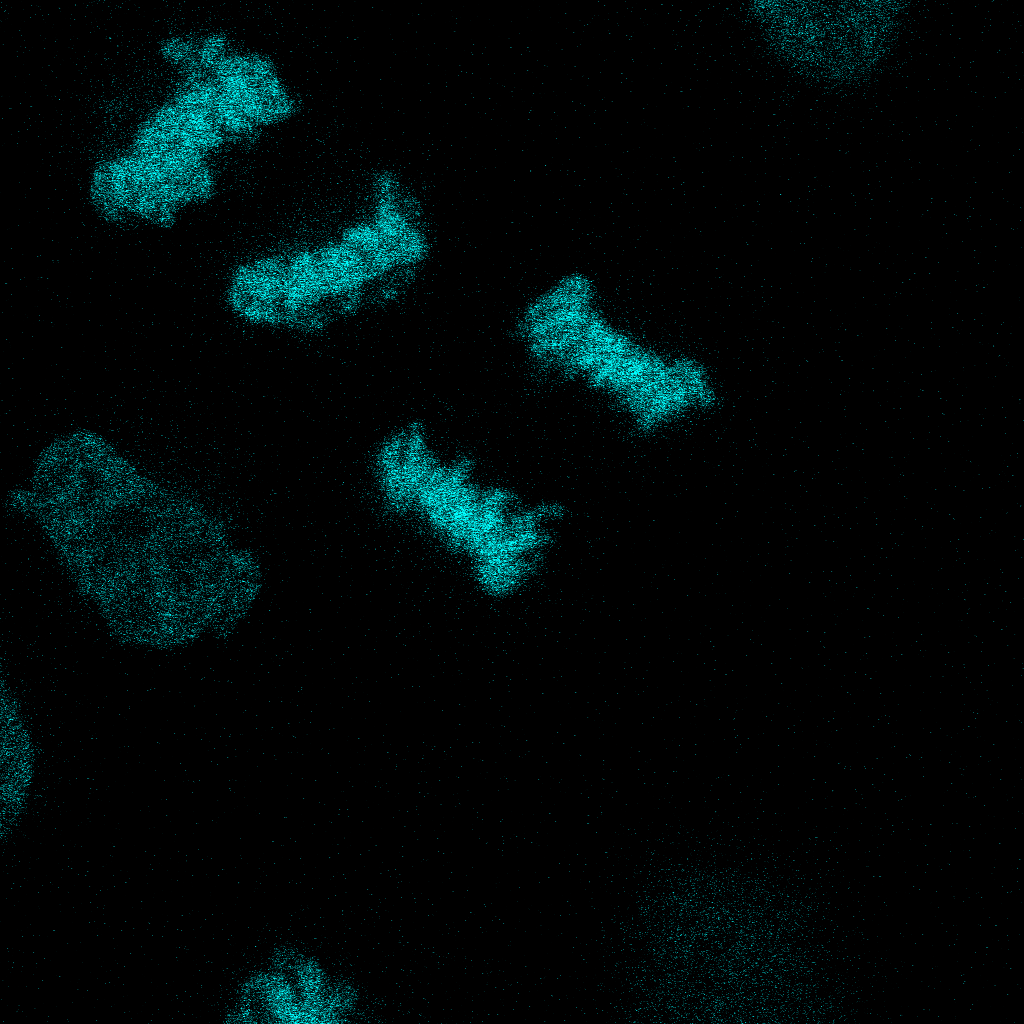

Supplement: Supplementary file 14 — Source data Fig. 6 [file 44319_2024_125_MOESM14_ESM.zip › Figure 6/Figure 6C/GFP VAPB_ELYS_DAPI.tif]

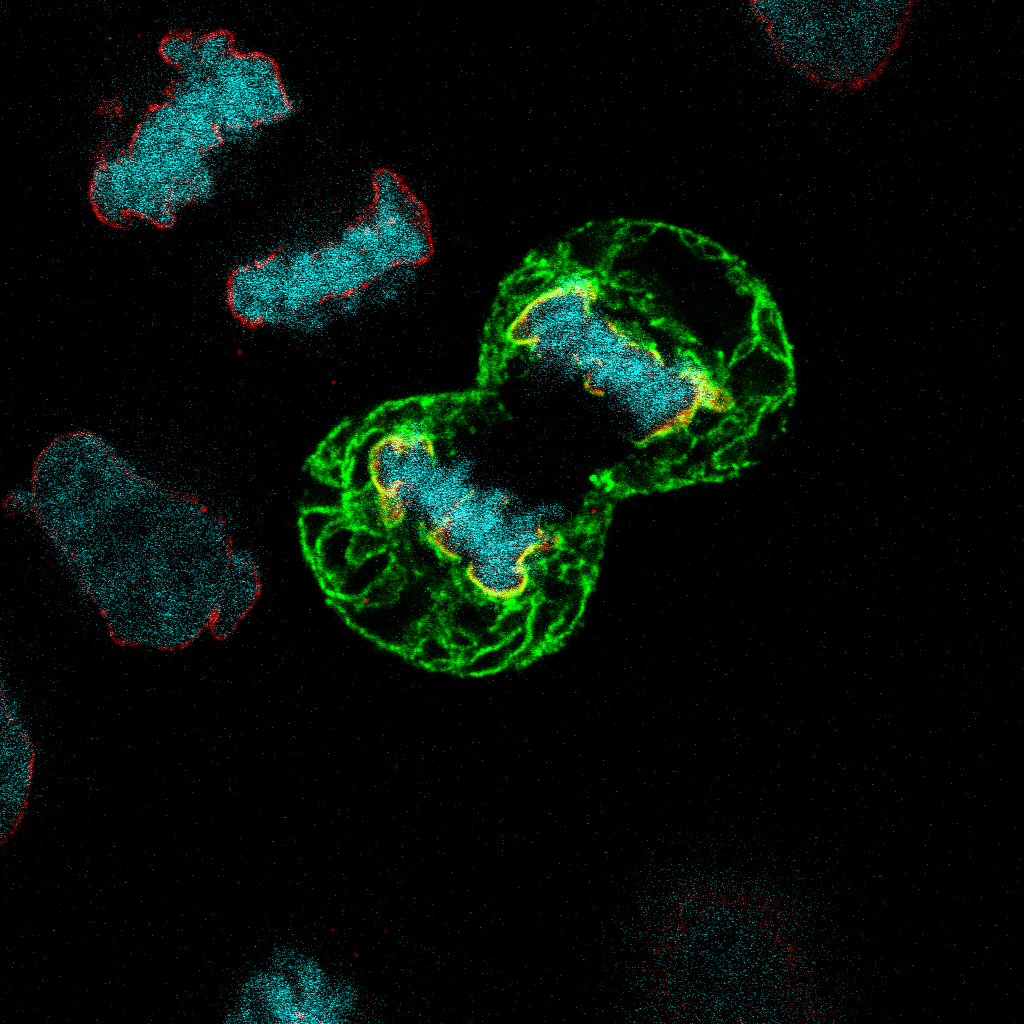

Supplement: Supplementary file 14 — Source data Fig. 6 [file 44319_2024_125_MOESM14_ESM.zip › Figure 6/Figure 6C/GFP VAPB_ELYS_merge.tif]

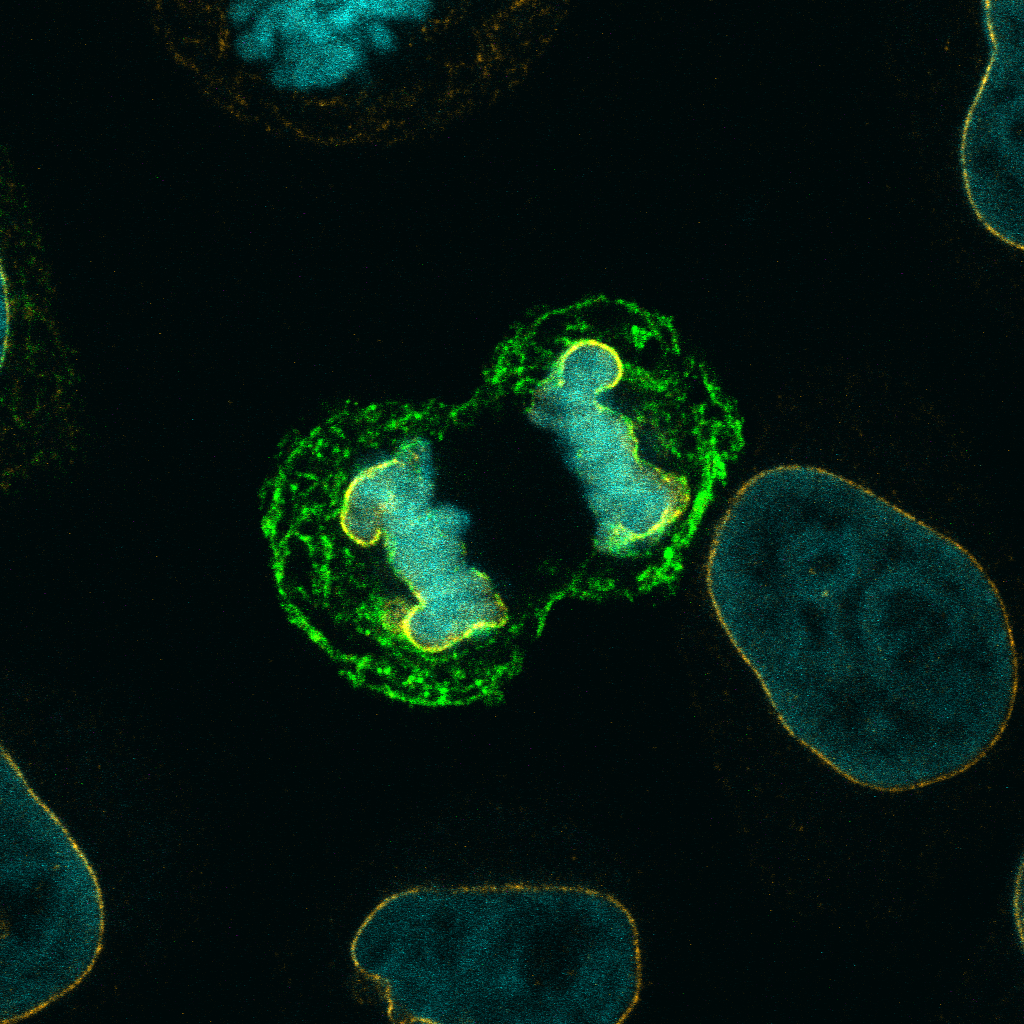

Supplement: Supplementary file 14 — Source data Fig. 6 [file 44319_2024_125_MOESM14_ESM.zip › Figure 6/Figure 6C/GFP VAPB_LBR_merge.tif]

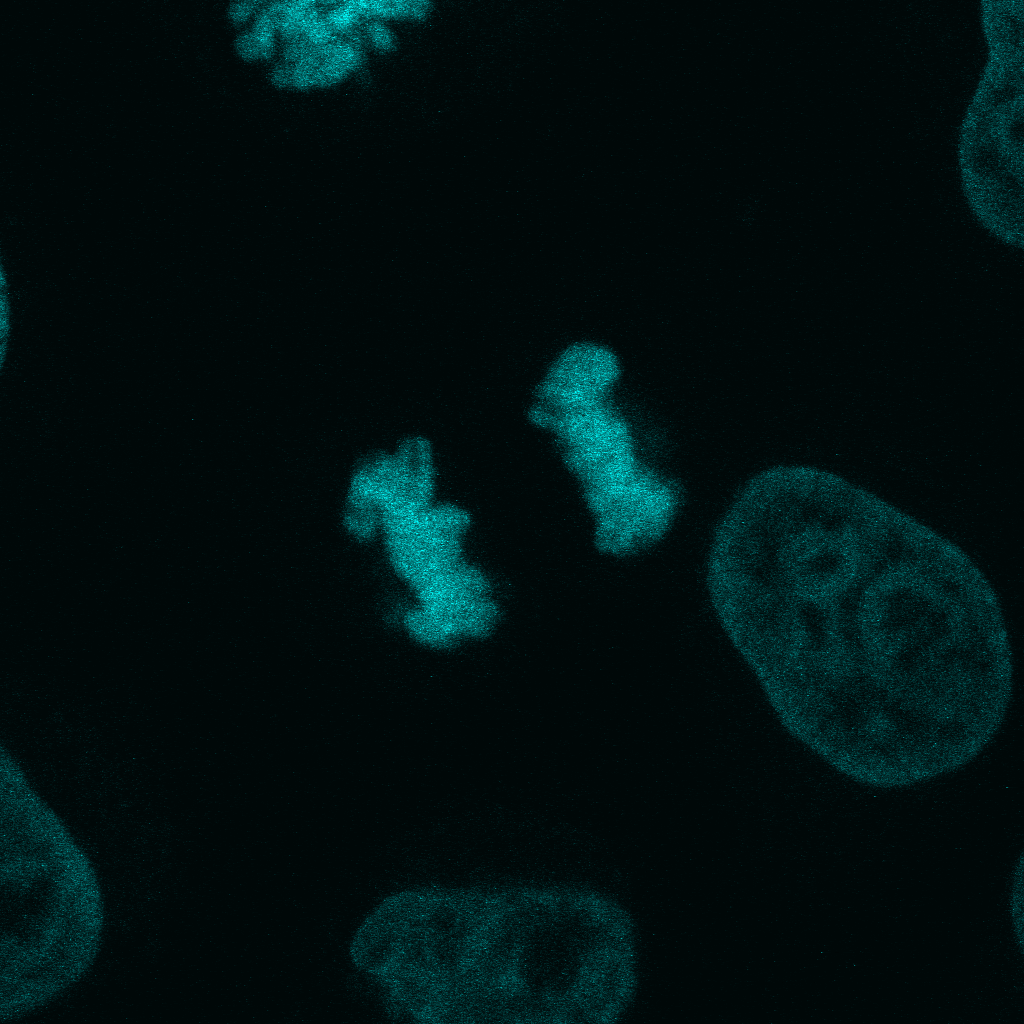

Supplement: Supplementary file 14 — Source data Fig. 6 [file 44319_2024_125_MOESM14_ESM.zip › Figure 6/Figure 6C/GFP VAPB_LBR_DAPI.tif]

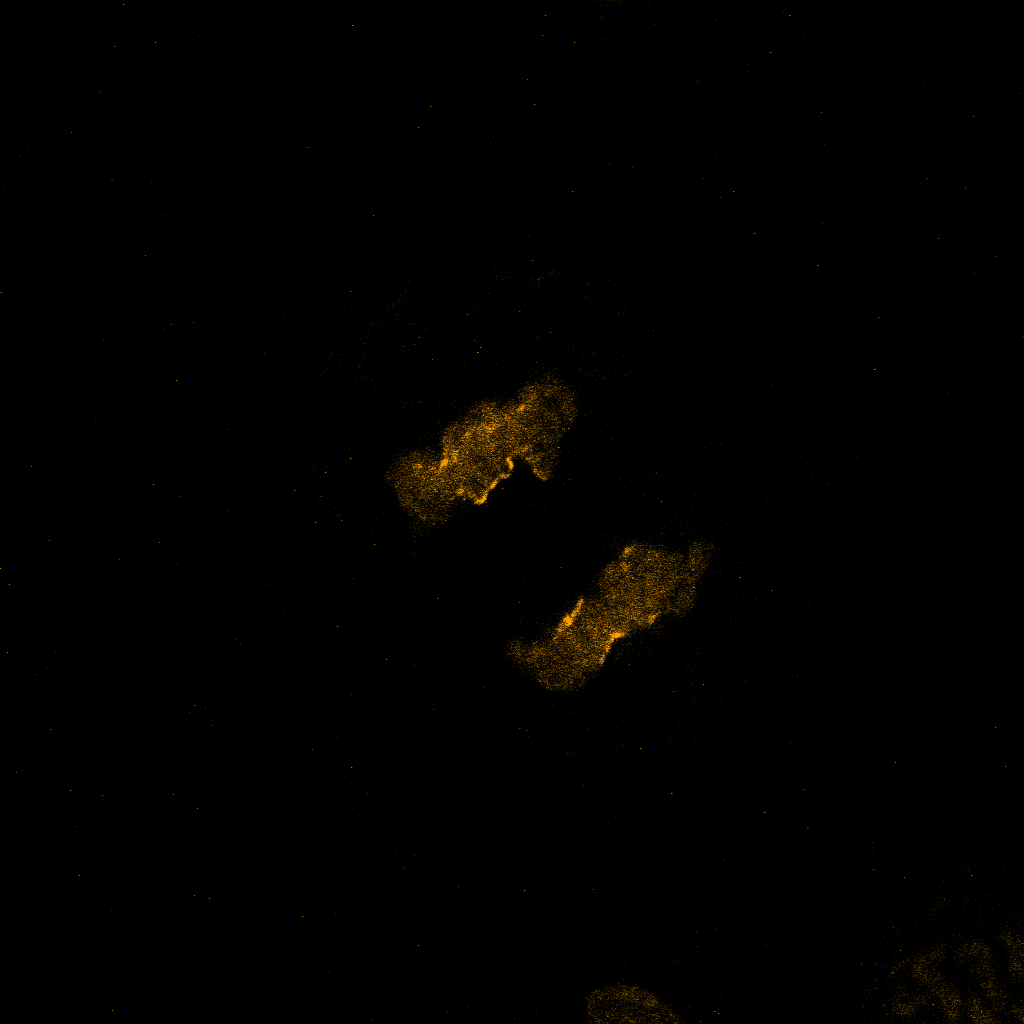

Supplement: Supplementary file 14 — Source data Fig. 6 [file 44319_2024_125_MOESM14_ESM.zip › Figure 6/Figure 6C/GFP VAPB_emerin_emerin.tif]

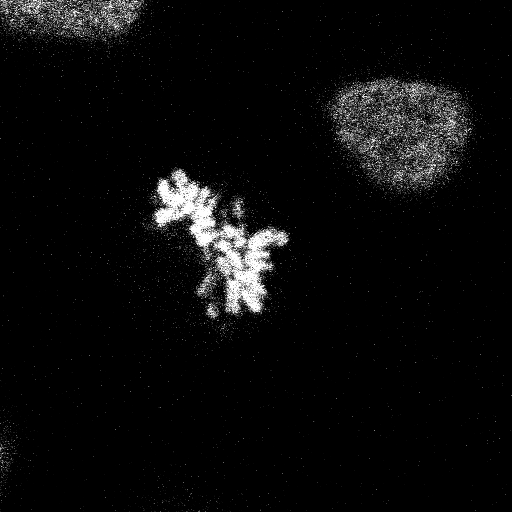

Supplement: Supplementary file 14 — Source data Fig. 6 [file 44319_2024_125_MOESM14_ESM.zip › Figure 6/Figure 6D/metaphase/sint metaphase_DAPI.tif]

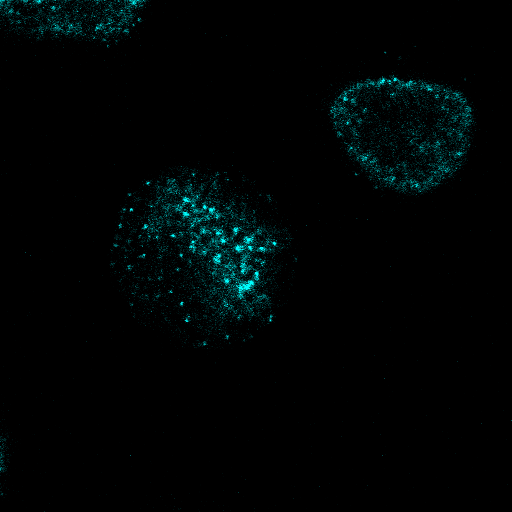

Supplement: Supplementary file 14 — Source data Fig. 6 [file 44319_2024_125_MOESM14_ESM.zip › Figure 6/Figure 6D/metaphase/sint metaphase_ELYS.tif]

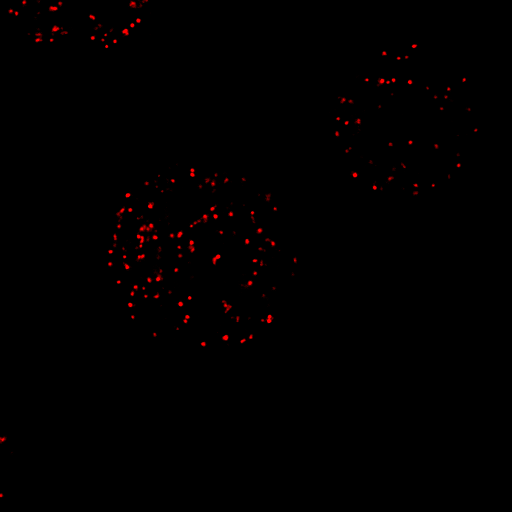

Supplement: Supplementary file 14 — Source data Fig. 6 [file 44319_2024_125_MOESM14_ESM.zip › Figure 6/Figure 6D/metaphase/sint metaphase_PLA.tif]

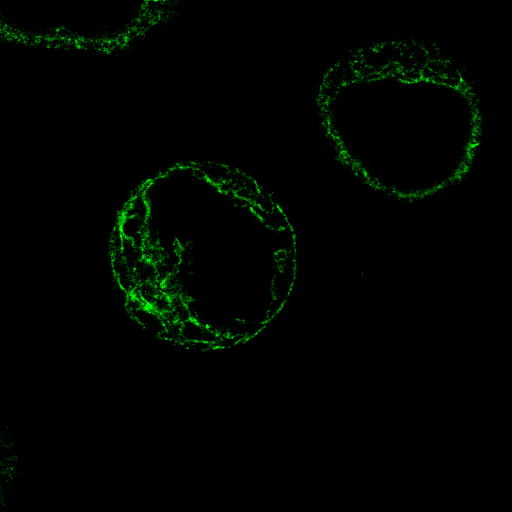

Supplement: Supplementary file 14 — Source data Fig. 6 [file 44319_2024_125_MOESM14_ESM.zip › Figure 6/Figure 6D/metaphase/sint metaphase_HA.tif]

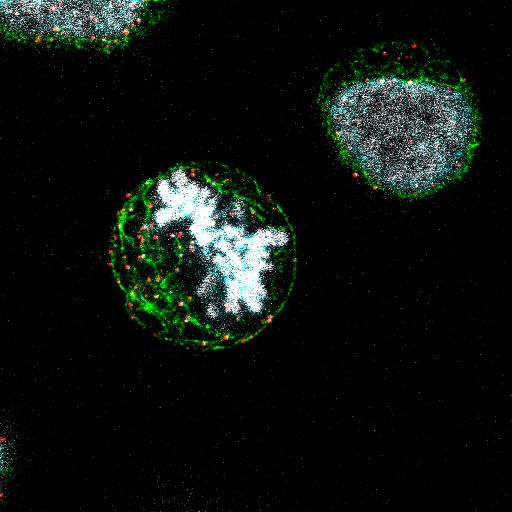

Supplement: Supplementary file 14 — Source data Fig. 6 [file 44319_2024_125_MOESM14_ESM.zip › Figure 6/Figure 6D/metaphase/sint metaphase_merge.tif]

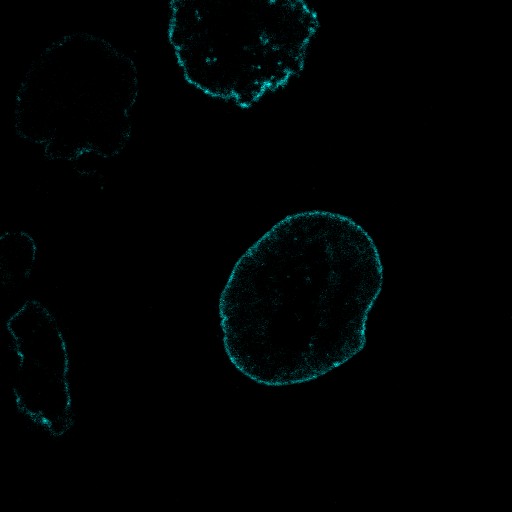

Supplement: Supplementary file 14 — Source data Fig. 6 [file 44319_2024_125_MOESM14_ESM.zip › Figure 6/Figure 6D/interphase/sint interphase_ELYS.tif]

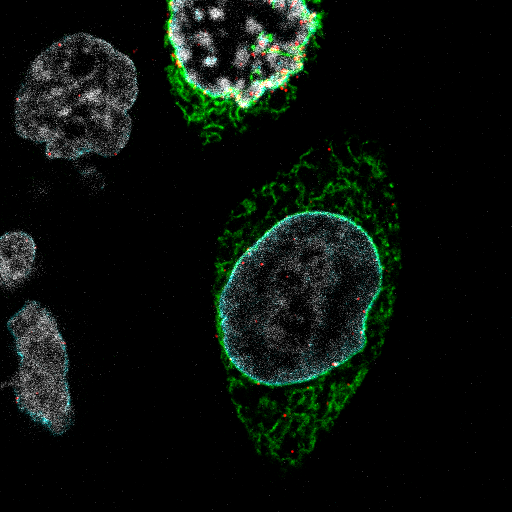

Supplement: Supplementary file 14 — Source data Fig. 6 [file 44319_2024_125_MOESM14_ESM.zip › Figure 6/Figure 6D/interphase/sint interphase_merge.tif]

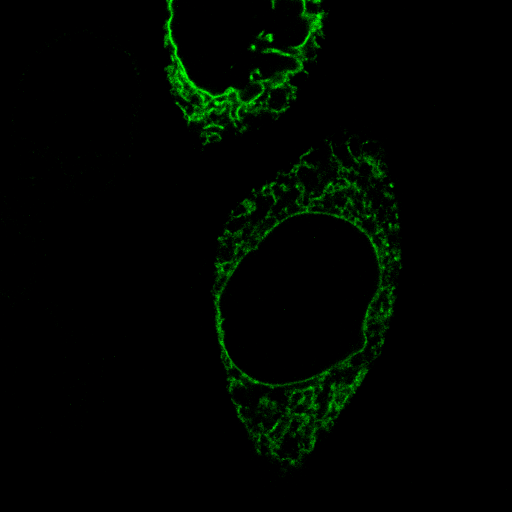

Supplement: Supplementary file 14 — Source data Fig. 6 [file 44319_2024_125_MOESM14_ESM.zip › Figure 6/Figure 6D/interphase/sint interphase_HA.tif]

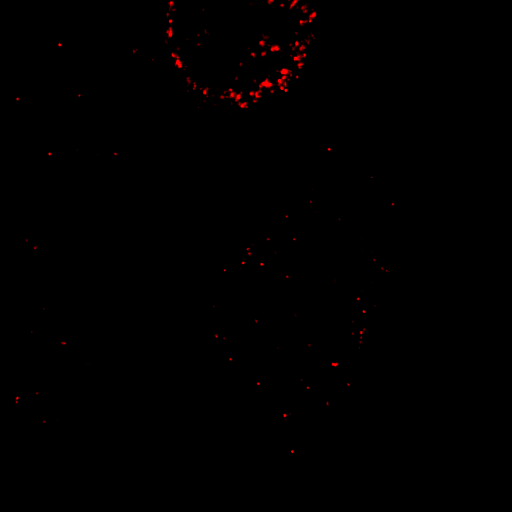

Supplement: Supplementary file 14 — Source data Fig. 6 [file 44319_2024_125_MOESM14_ESM.zip › Figure 6/Figure 6D/interphase/sint interphase_PLA.tif]

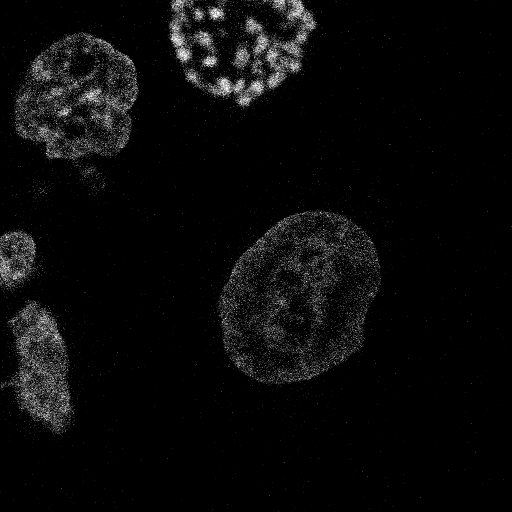

Supplement: Supplementary file 14 — Source data Fig. 6 [file 44319_2024_125_MOESM14_ESM.zip › Figure 6/Figure 6D/interphase/sint interphase_DAPI.tif]

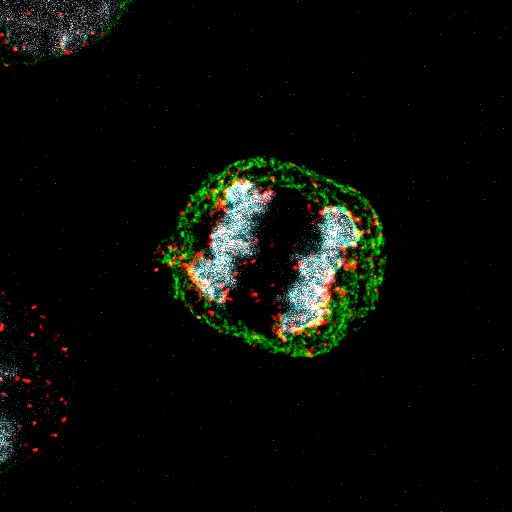

Supplement: Supplementary file 14 — Source data Fig. 6 [file 44319_2024_125_MOESM14_ESM.zip › Figure 6/Figure 6D/anaphase/sint anaphase 02_merge.tif]

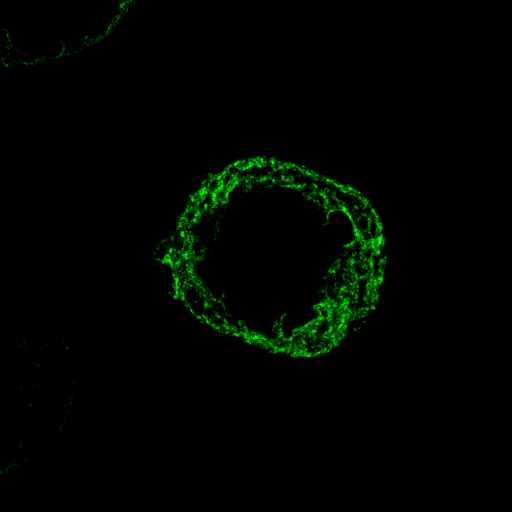

Supplement: Supplementary file 14 — Source data Fig. 6 [file 44319_2024_125_MOESM14_ESM.zip › Figure 6/Figure 6D/anaphase/sint anaphase 02_HA.tif]

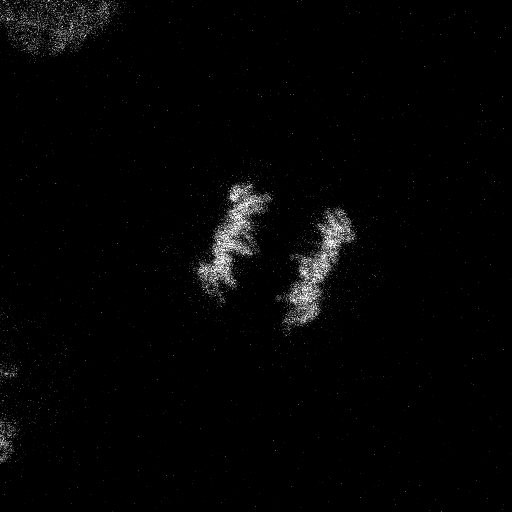

Supplement: Supplementary file 14 — Source data Fig. 6 [file 44319_2024_125_MOESM14_ESM.zip › Figure 6/Figure 6D/anaphase/sint anaphase 02_DAPI.tif]

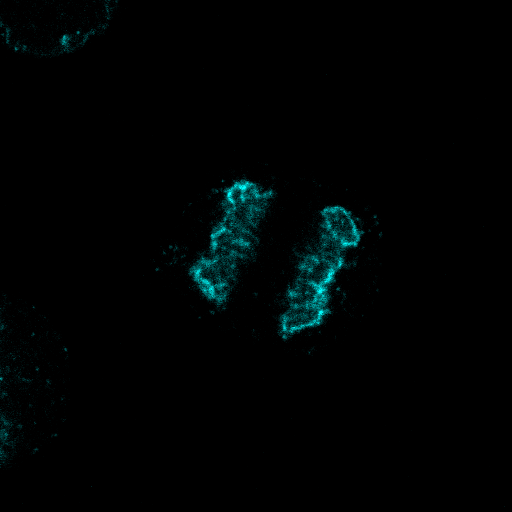

Supplement: Supplementary file 14 — Source data Fig. 6 [file 44319_2024_125_MOESM14_ESM.zip › Figure 6/Figure 6D/anaphase/sint anaphase 02_ELYS.tif]

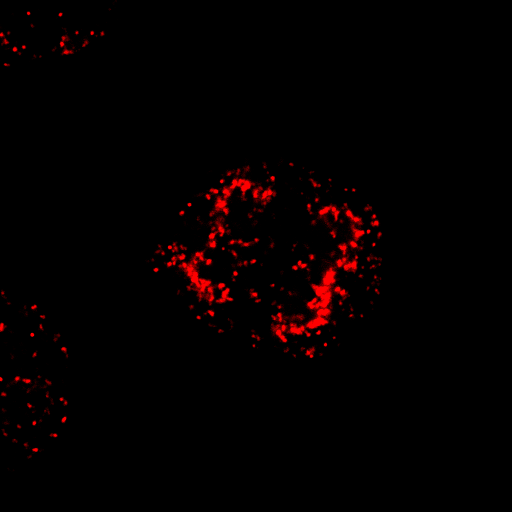

Supplement: Supplementary file 14 — Source data Fig. 6 [file 44319_2024_125_MOESM14_ESM.zip › Figure 6/Figure 6D/anaphase/sint anaphase 02_PLA.tif]

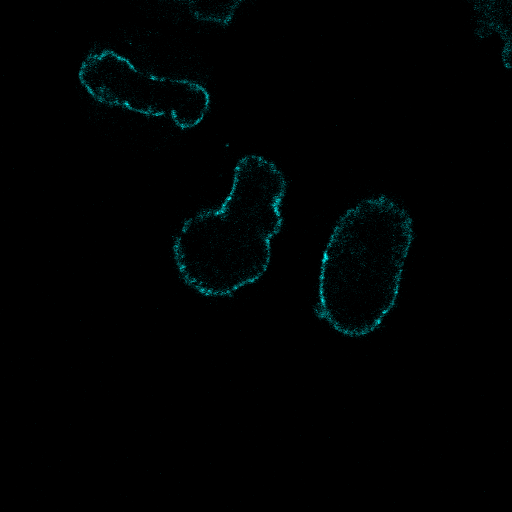

Supplement: Supplementary file 14 — Source data Fig. 6 [file 44319_2024_125_MOESM14_ESM.zip › Figure 6/Figure 6D/telophase/sint telophase_ELYS.tif]

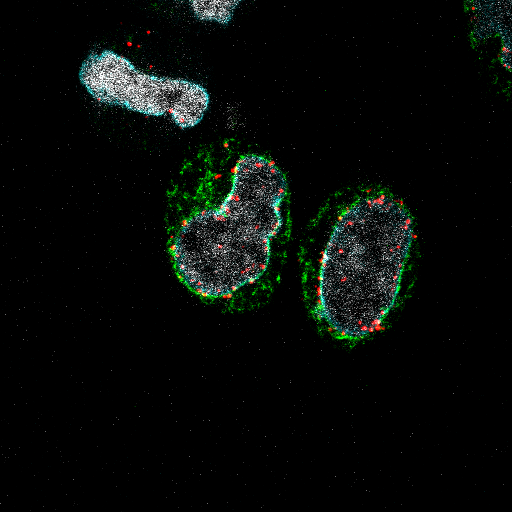

Supplement: Supplementary file 14 — Source data Fig. 6 [file 44319_2024_125_MOESM14_ESM.zip › Figure 6/Figure 6D/telophase/sint telophase_merge.tif]

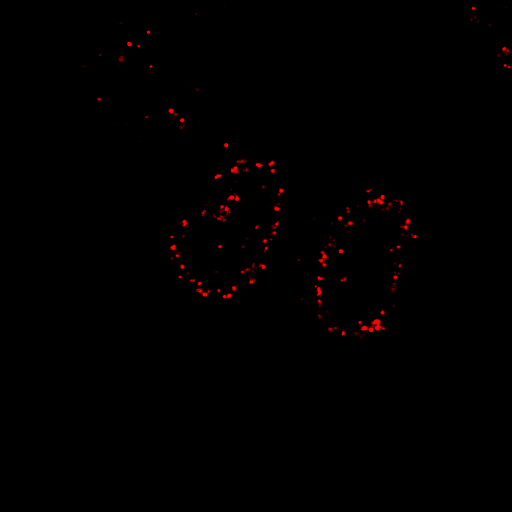

Supplement: Supplementary file 14 — Source data Fig. 6 [file 44319_2024_125_MOESM14_ESM.zip › Figure 6/Figure 6D/telophase/sint telophase_PLA.tif]

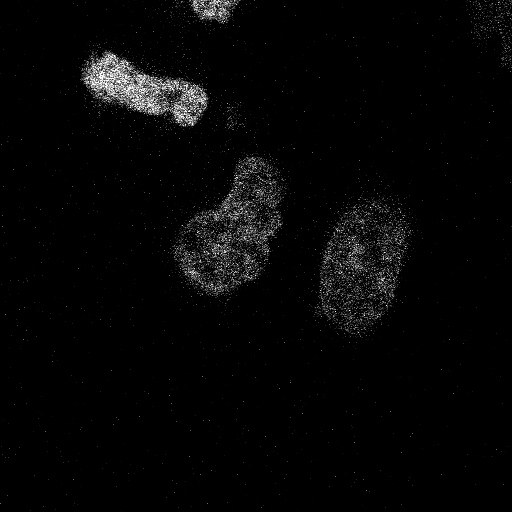

Supplement: Supplementary file 14 — Source data Fig. 6 [file 44319_2024_125_MOESM14_ESM.zip › Figure 6/Figure 6D/telophase/sint telophase_DAPI.tif]

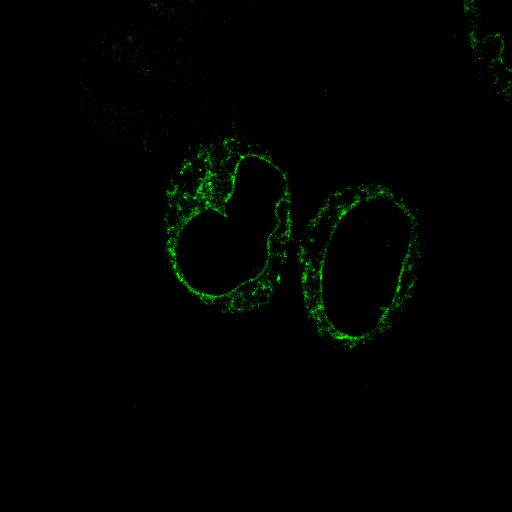

Supplement: Supplementary file 14 — Source data Fig. 6 [file 44319_2024_125_MOESM14_ESM.zip › Figure 6/Figure 6D/telophase/sint telophase_HA.tif]

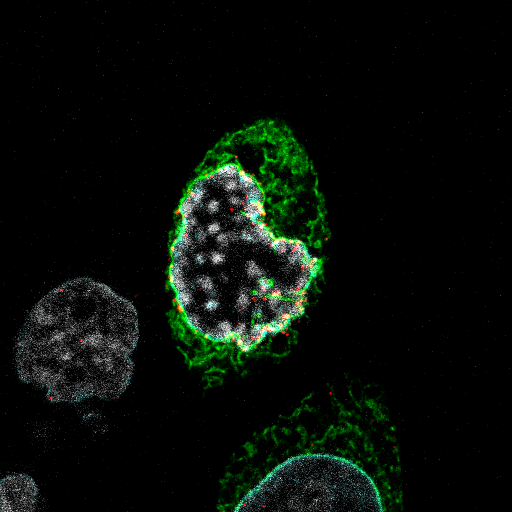

Supplement: Supplementary file 14 — Source data Fig. 6 [file 44319_2024_125_MOESM14_ESM.zip › Figure 6/Figure 6D/prophase/sint prophase_merge.tif]

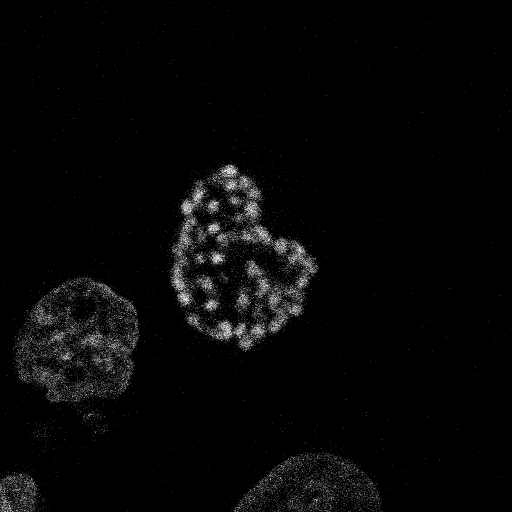

Supplement: Supplementary file 14 — Source data Fig. 6 [file 44319_2024_125_MOESM14_ESM.zip › Figure 6/Figure 6D/prophase/sint prophase_DAPI.tif]

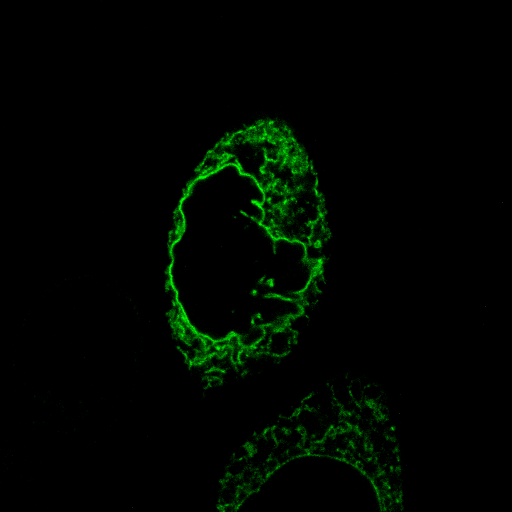

Supplement: Supplementary file 14 — Source data Fig. 6 [file 44319_2024_125_MOESM14_ESM.zip › Figure 6/Figure 6D/prophase/sint prophase_HA.tif]

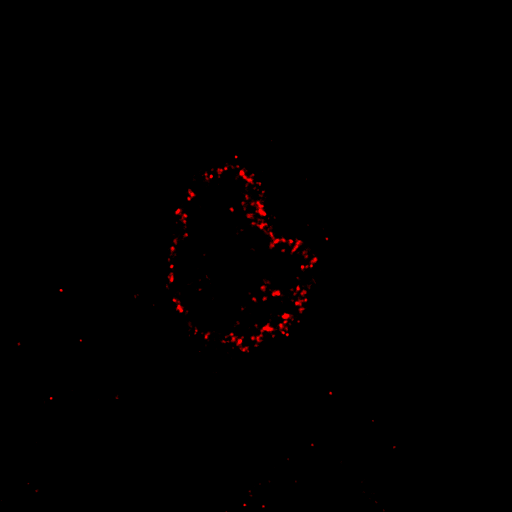

Supplement: Supplementary file 14 — Source data Fig. 6 [file 44319_2024_125_MOESM14_ESM.zip › Figure 6/Figure 6D/prophase/sint prophase_PLA.tif]

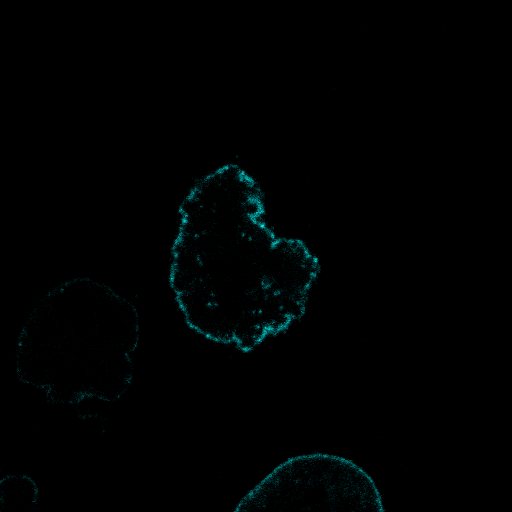

Supplement: Supplementary file 14 — Source data Fig. 6 [file 44319_2024_125_MOESM14_ESM.zip › Figure 6/Figure 6D/prophase/sint prophase_ELYS.tif]

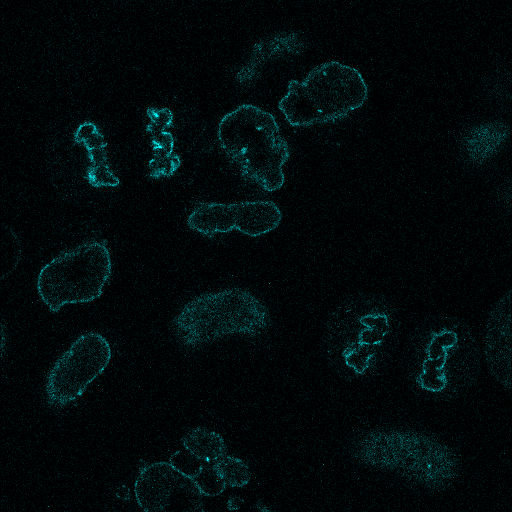

Supplement: Supplementary file 15 — Source data Fig. 7 [file 44319_2024_125_MOESM15_ESM.zip › Figure 7/Figure 7A/HA-VAPB_ELYS_WT_anaphase/HA elys 01_ELYS.tif]

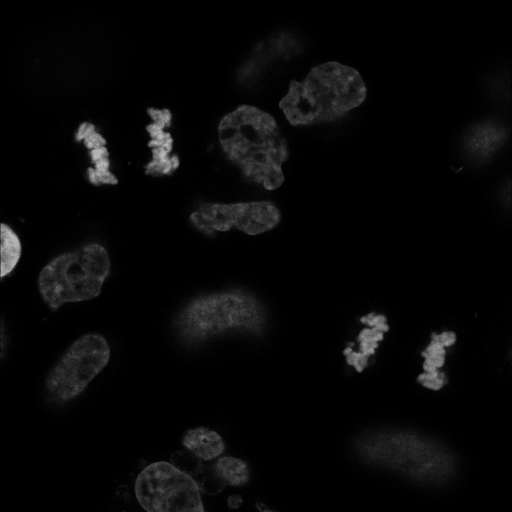

Supplement: Supplementary file 15 — Source data Fig. 7 [file 44319_2024_125_MOESM15_ESM.zip › Figure 7/Figure 7A/HA-VAPB_ELYS_WT_anaphase/HA elys 01_DAPI.tif]

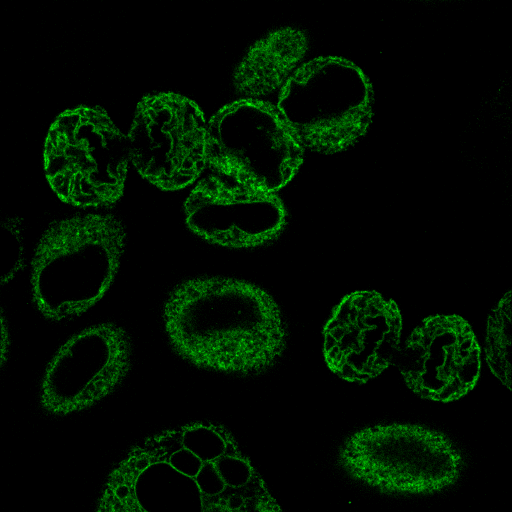

Supplement: Supplementary file 15 — Source data Fig. 7 [file 44319_2024_125_MOESM15_ESM.zip › Figure 7/Figure 7A/HA-VAPB_ELYS_WT_anaphase/HA elys 01_HA.tif]

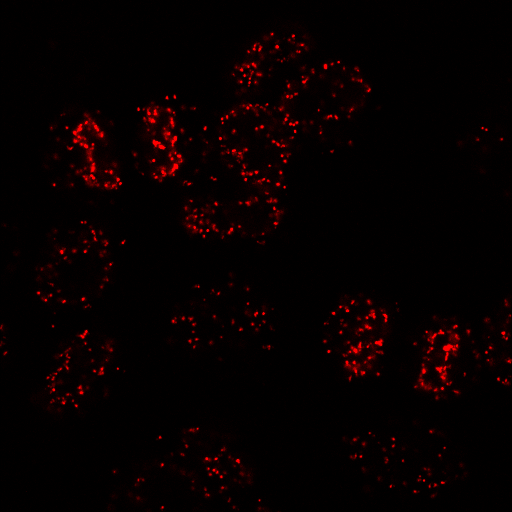

Supplement: Supplementary file 15 — Source data Fig. 7 [file 44319_2024_125_MOESM15_ESM.zip › Figure 7/Figure 7A/HA-VAPB_ELYS_WT_anaphase/HA elys 01_PLA.tif]

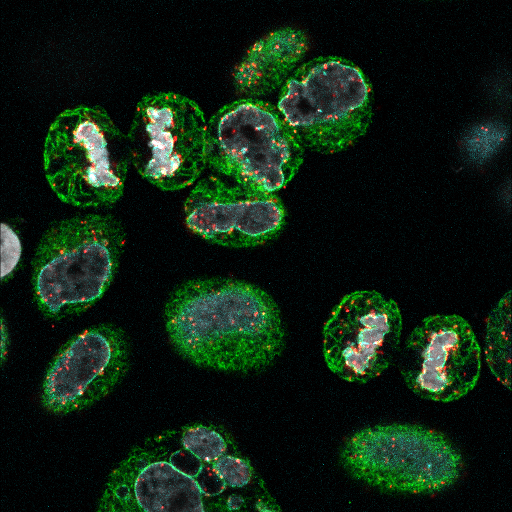

Supplement: Supplementary file 15 — Source data Fig. 7 [file 44319_2024_125_MOESM15_ESM.zip › Figure 7/Figure 7A/HA-VAPB_ELYS_WT_anaphase/HA elys 01_merge.tif]

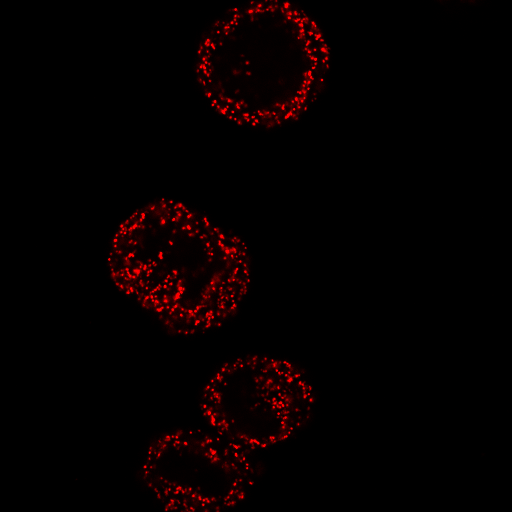

Supplement: Supplementary file 15 — Source data Fig. 7 [file 44319_2024_125_MOESM15_ESM.zip › Figure 7/Figure 7A/HA-VAPB_emerin_KD_MD_anaphase/HA emd 06_PLA.tif]

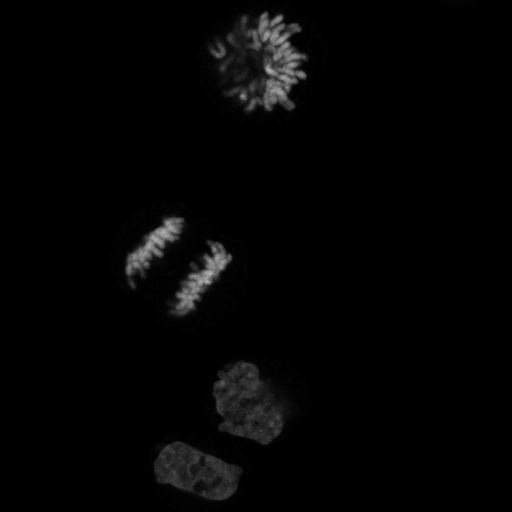

Supplement: Supplementary file 15 — Source data Fig. 7 [file 44319_2024_125_MOESM15_ESM.zip › Figure 7/Figure 7A/HA-VAPB_emerin_KD_MD_anaphase/HA emd 06_DAPI.tif]

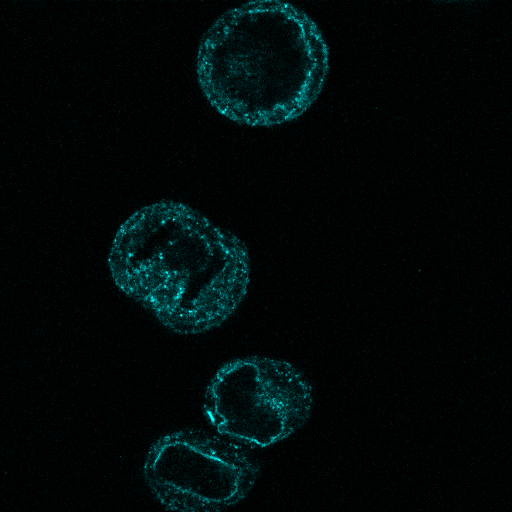

Supplement: Supplementary file 15 — Source data Fig. 7 [file 44319_2024_125_MOESM15_ESM.zip › Figure 7/Figure 7A/HA-VAPB_emerin_KD_MD_anaphase/HA emd 06_emerin.tif]

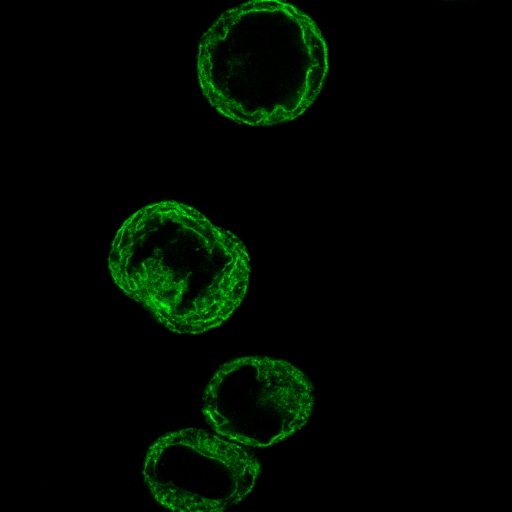

Supplement: Supplementary file 15 — Source data Fig. 7 [file 44319_2024_125_MOESM15_ESM.zip › Figure 7/Figure 7A/HA-VAPB_emerin_KD_MD_anaphase/HA emd 06_HA.tif]

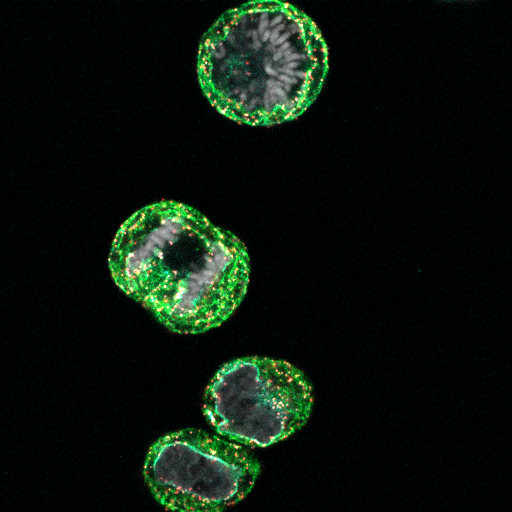

Supplement: Supplementary file 15 — Source data Fig. 7 [file 44319_2024_125_MOESM15_ESM.zip › Figure 7/Figure 7A/HA-VAPB_emerin_KD_MD_anaphase/HA emd 06_merge.tif]

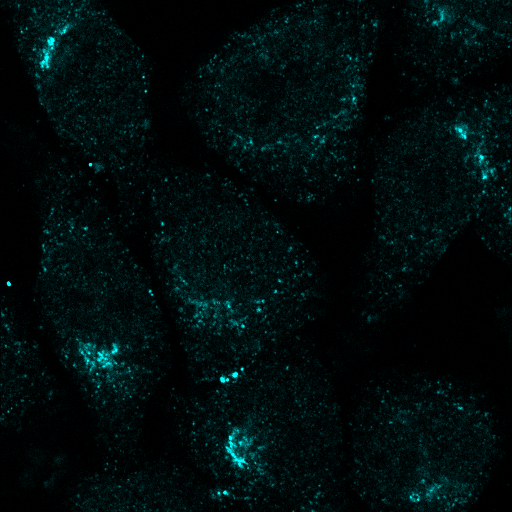

Supplement: Supplementary file 15 — Source data Fig. 7 [file 44319_2024_125_MOESM15_ESM.zip › Figure 7/Figure 7A/HA-VAPB_OSBPL9_WT_interphase cells/HA osbp interphase_osbpl9.tif]

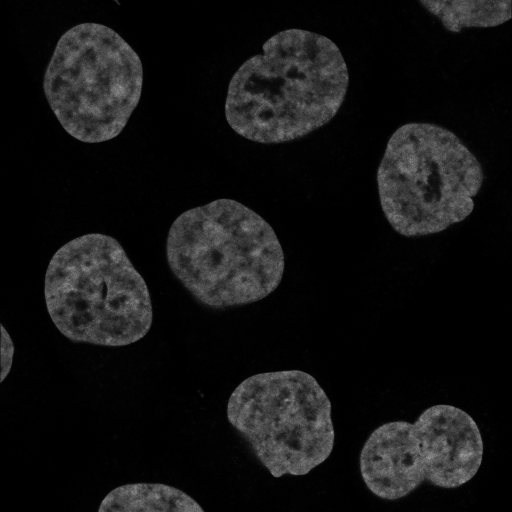

Supplement: Supplementary file 15 — Source data Fig. 7 [file 44319_2024_125_MOESM15_ESM.zip › Figure 7/Figure 7A/HA-VAPB_OSBPL9_WT_interphase cells/HA osbp interphase_DAPI.tif]

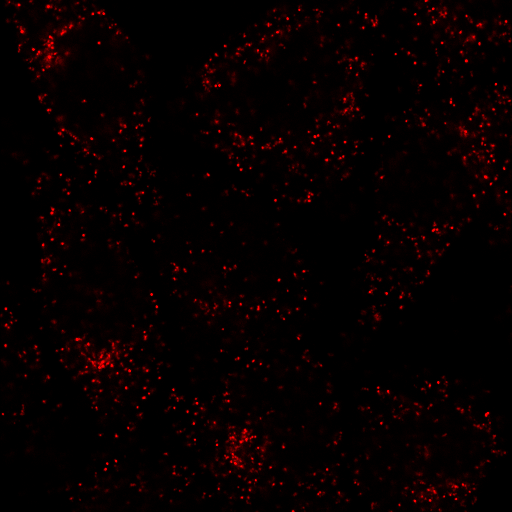

Supplement: Supplementary file 15 — Source data Fig. 7 [file 44319_2024_125_MOESM15_ESM.zip › Figure 7/Figure 7A/HA-VAPB_OSBPL9_WT_interphase cells/HA osbp interphase_PLA.tif]

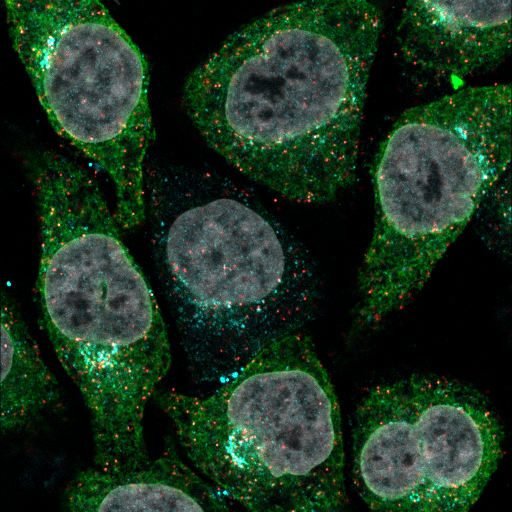

Supplement: Supplementary file 15 — Source data Fig. 7 [file 44319_2024_125_MOESM15_ESM.zip › Figure 7/Figure 7A/HA-VAPB_OSBPL9_WT_interphase cells/HA osbp interphase_merge.tif]

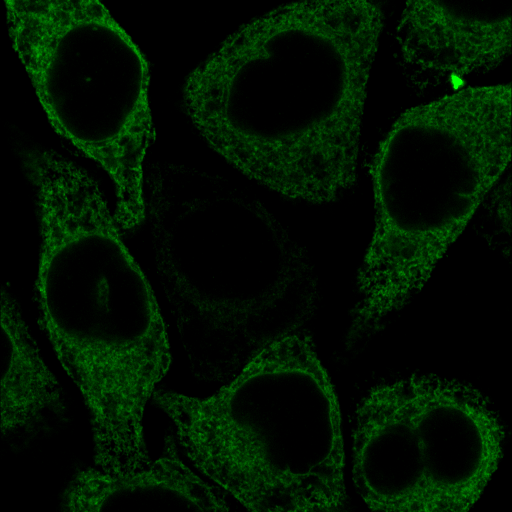

Supplement: Supplementary file 15 — Source data Fig. 7 [file 44319_2024_125_MOESM15_ESM.zip › Figure 7/Figure 7A/HA-VAPB_OSBPL9_WT_interphase cells/HA osbp interphase_HA.tif]

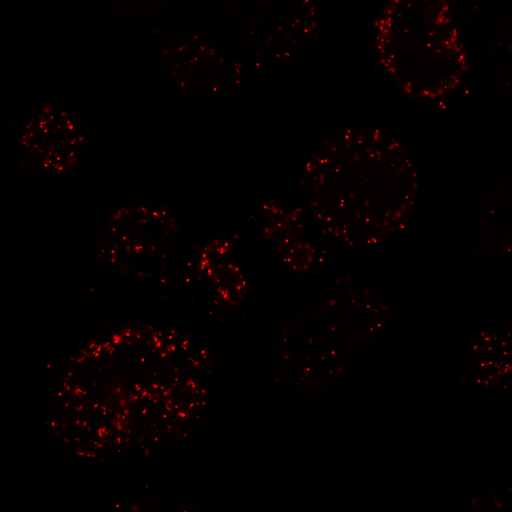

Supplement: Supplementary file 15 — Source data Fig. 7 [file 44319_2024_125_MOESM15_ESM.zip › Figure 7/Figure 7A/HA-VAPB_ELYS_KD_MD_anaphase/HA ELZS 04_PLA.tif]

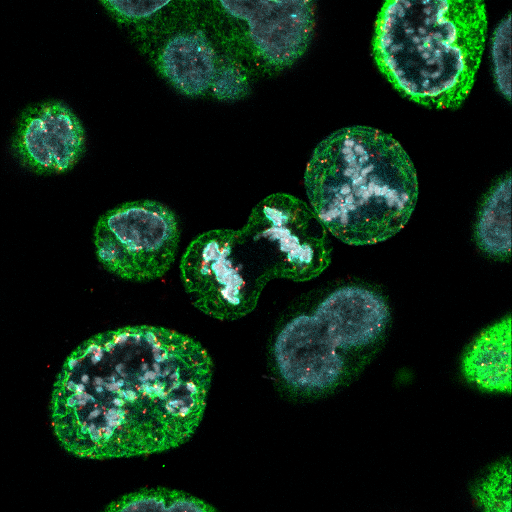

Supplement: Supplementary file 15 — Source data Fig. 7 [file 44319_2024_125_MOESM15_ESM.zip › Figure 7/Figure 7A/HA-VAPB_ELYS_KD_MD_anaphase/HA ELZS 04_merge.tif]

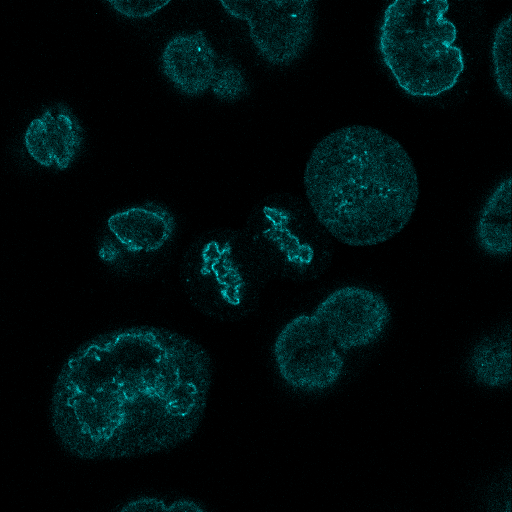

Supplement: Supplementary file 15 — Source data Fig. 7 [file 44319_2024_125_MOESM15_ESM.zip › Figure 7/Figure 7A/HA-VAPB_ELYS_KD_MD_anaphase/HA ELZS 04_ELYS.tif]

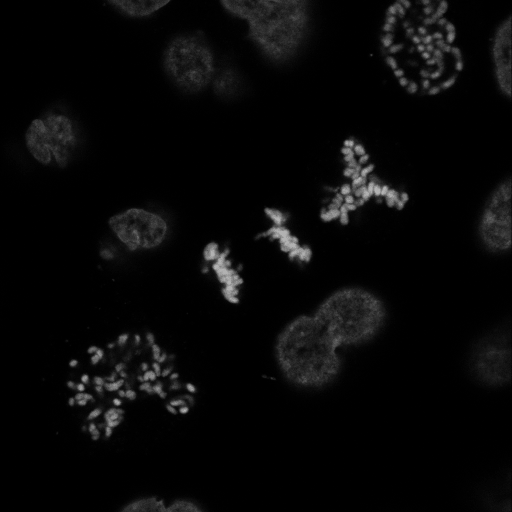

Supplement: Supplementary file 15 — Source data Fig. 7 [file 44319_2024_125_MOESM15_ESM.zip › Figure 7/Figure 7A/HA-VAPB_ELYS_KD_MD_anaphase/HA ELZS 04_DAPI.tif]

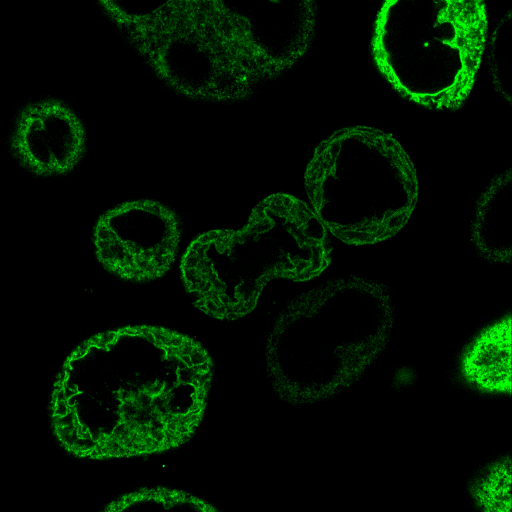

Supplement: Supplementary file 15 — Source data Fig. 7 [file 44319_2024_125_MOESM15_ESM.zip › Figure 7/Figure 7A/HA-VAPB_ELYS_KD_MD_anaphase/HA ELZS 04_HA.tif]

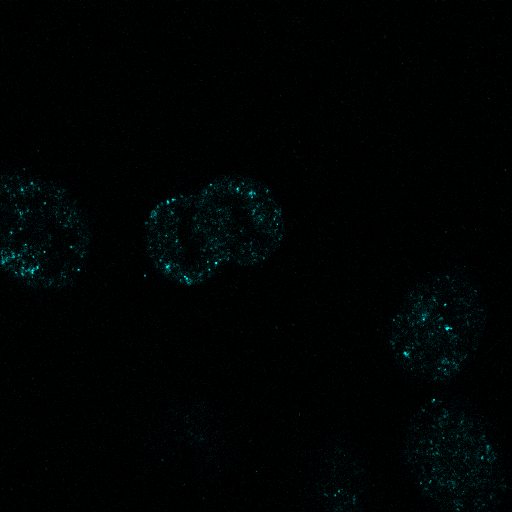

Supplement: Supplementary file 15 — Source data Fig. 7 [file 44319_2024_125_MOESM15_ESM.zip › Figure 7/Figure 7A/HA-VAPB_OSBPL9_KD_MD_anaphase/HA OSBP 05_OSBPL9.tif]

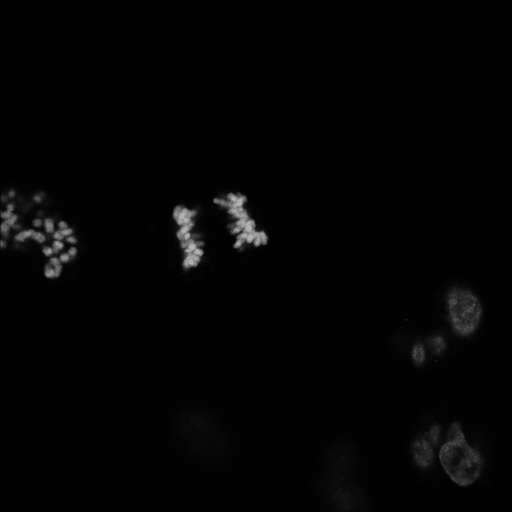

Supplement: Supplementary file 15 — Source data Fig. 7 [file 44319_2024_125_MOESM15_ESM.zip › Figure 7/Figure 7A/HA-VAPB_OSBPL9_KD_MD_anaphase/HA OSBP 05_DAPI.tif]

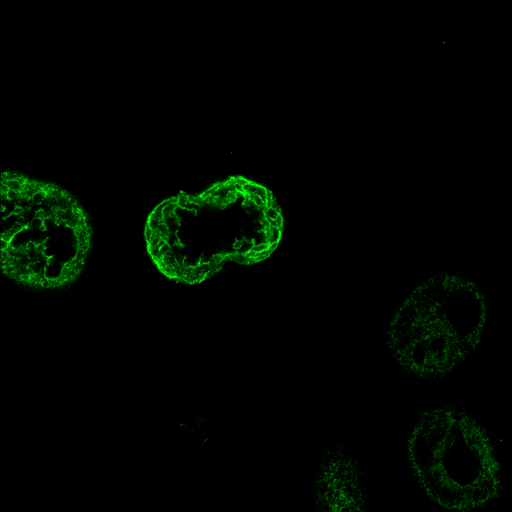

Supplement: Supplementary file 15 — Source data Fig. 7 [file 44319_2024_125_MOESM15_ESM.zip › Figure 7/Figure 7A/HA-VAPB_OSBPL9_KD_MD_anaphase/HA OSBP 05_HA.tif]

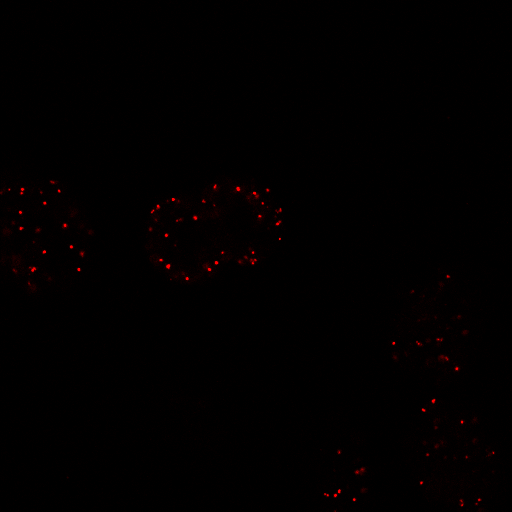

Supplement: Supplementary file 15 — Source data Fig. 7 [file 44319_2024_125_MOESM15_ESM.zip › Figure 7/Figure 7A/HA-VAPB_OSBPL9_KD_MD_anaphase/HA OSBP 05_PLA.tif]

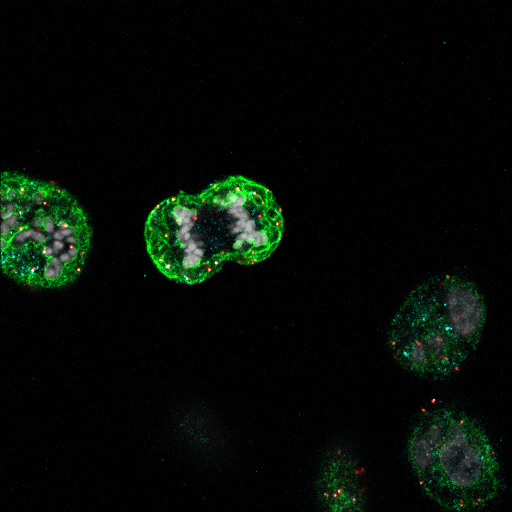

Supplement: Supplementary file 15 — Source data Fig. 7 [file 44319_2024_125_MOESM15_ESM.zip › Figure 7/Figure 7A/HA-VAPB_OSBPL9_KD_MD_anaphase/HA OSBP 05_merge.tif]

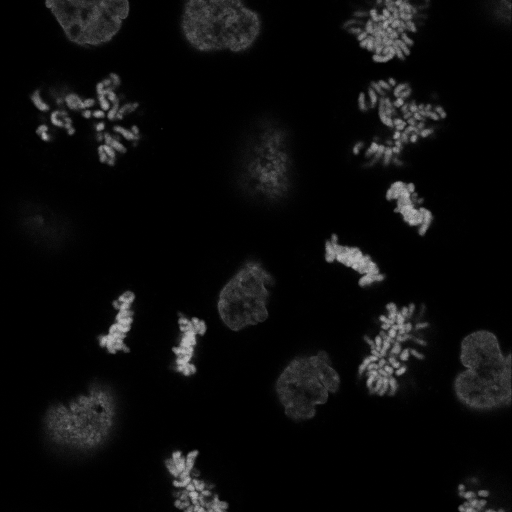

Supplement: Supplementary file 15 — Source data Fig. 7 [file 44319_2024_125_MOESM15_ESM.zip › Figure 7/Figure 7A/HA-VAPB_OSBPL9_WT_anaphase/HA osbpl9 05_DAPI.tif]

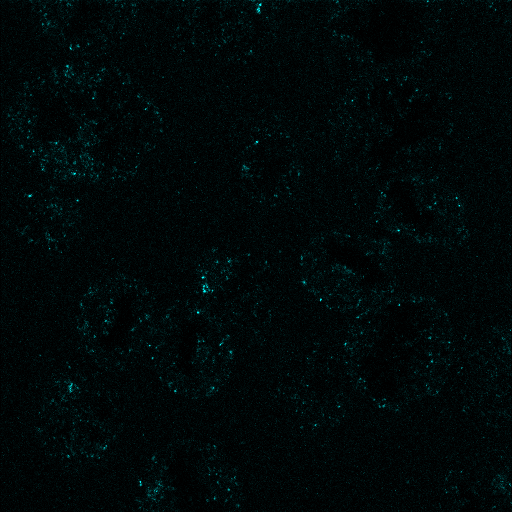

Supplement: Supplementary file 15 — Source data Fig. 7 [file 44319_2024_125_MOESM15_ESM.zip › Figure 7/Figure 7A/HA-VAPB_OSBPL9_WT_anaphase/HA osbpl9 05_OSBPL9.tif]

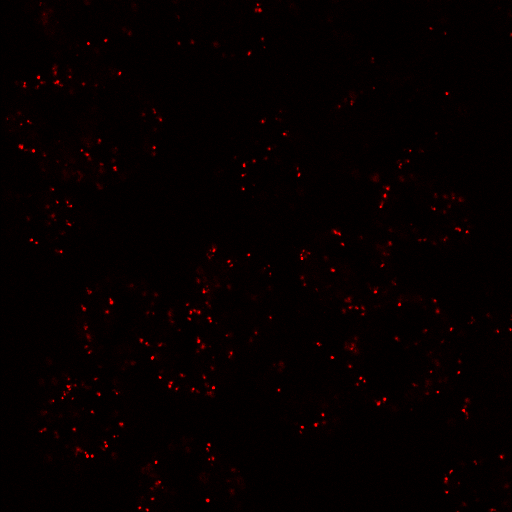

Supplement: Supplementary file 15 — Source data Fig. 7 [file 44319_2024_125_MOESM15_ESM.zip › Figure 7/Figure 7A/HA-VAPB_OSBPL9_WT_anaphase/HA osbpl9 05_PLA.tif]

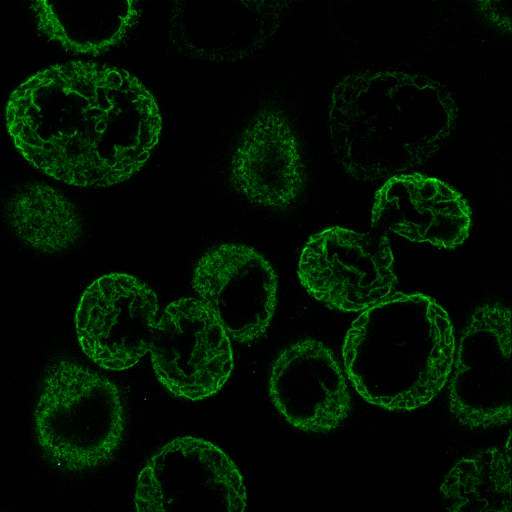

Supplement: Supplementary file 15 — Source data Fig. 7 [file 44319_2024_125_MOESM15_ESM.zip › Figure 7/Figure 7A/HA-VAPB_OSBPL9_WT_anaphase/HA osbpl9 05_HA.tif]

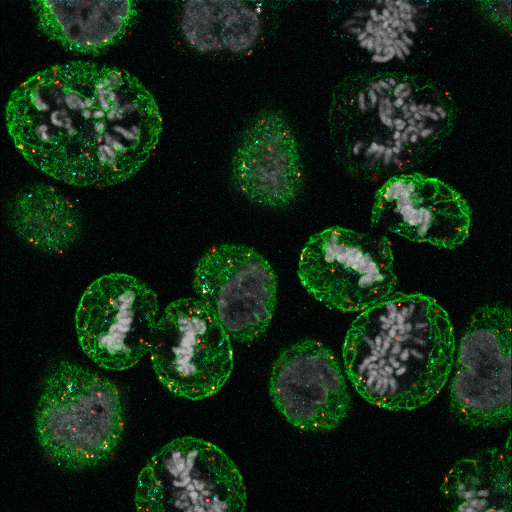

Supplement: Supplementary file 15 — Source data Fig. 7 [file 44319_2024_125_MOESM15_ESM.zip › Figure 7/Figure 7A/HA-VAPB_OSBPL9_WT_anaphase/HA osbpl9 05_merge.tif]

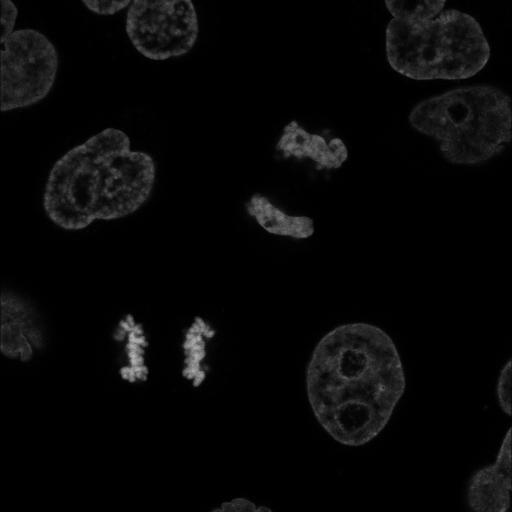

Supplement: Supplementary file 15 — Source data Fig. 7 [file 44319_2024_125_MOESM15_ESM.zip › Figure 7/Figure 7A/HA-VAPB_LBR_KD_MD_anaphase/HA LBR 09_DAPI.tif]

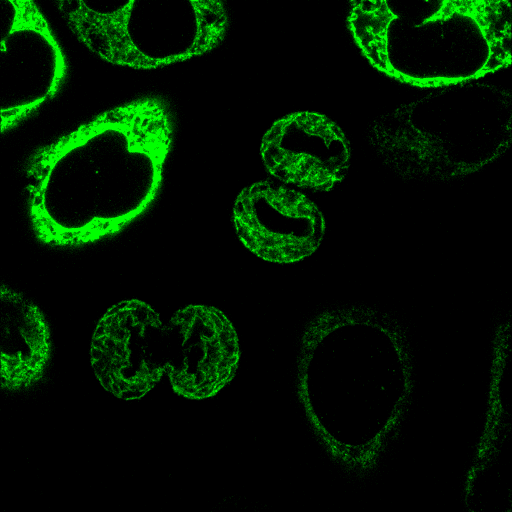

Supplement: Supplementary file 15 — Source data Fig. 7 [file 44319_2024_125_MOESM15_ESM.zip › Figure 7/Figure 7A/HA-VAPB_LBR_KD_MD_anaphase/HA LBR 09_HA.tif]

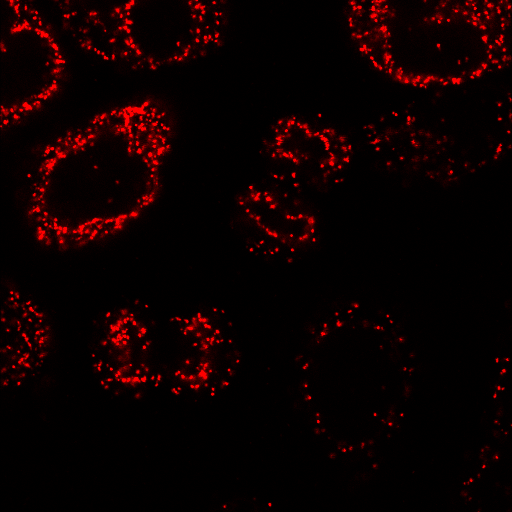

Supplement: Supplementary file 15 — Source data Fig. 7 [file 44319_2024_125_MOESM15_ESM.zip › Figure 7/Figure 7A/HA-VAPB_LBR_KD_MD_anaphase/HA LBR 09_PLA.tif]

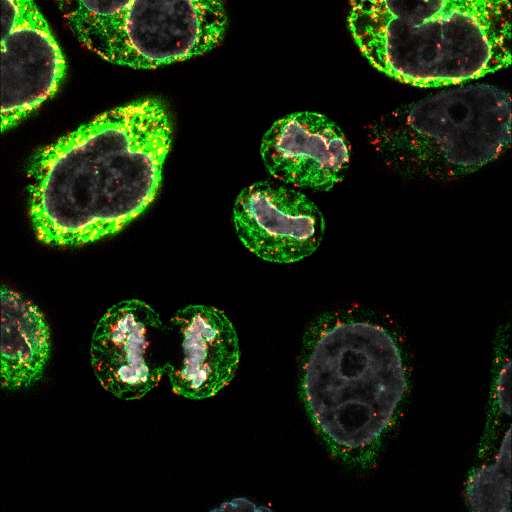

Supplement: Supplementary file 15 — Source data Fig. 7 [file 44319_2024_125_MOESM15_ESM.zip › Figure 7/Figure 7A/HA-VAPB_LBR_KD_MD_anaphase/HA LBR 09_merge.tif]

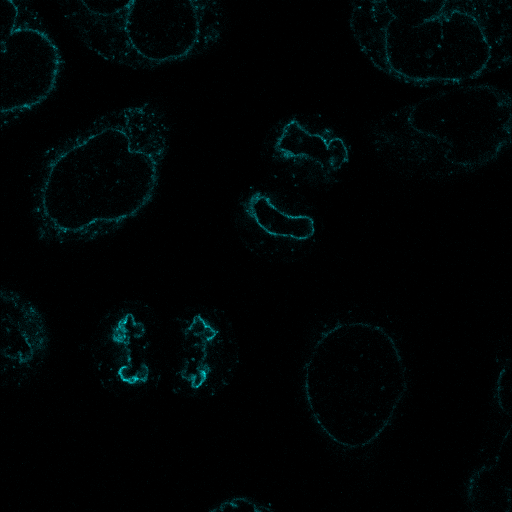

Supplement: Supplementary file 15 — Source data Fig. 7 [file 44319_2024_125_MOESM15_ESM.zip › Figure 7/Figure 7A/HA-VAPB_LBR_KD_MD_anaphase/HA LBR 09_LBR.tif]

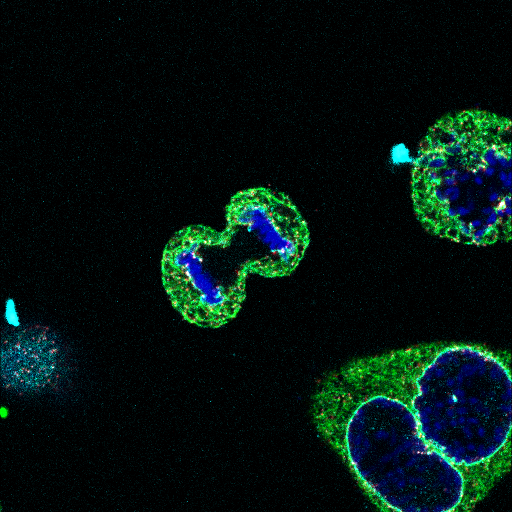

Supplement: Supplementary file 15 — Source data Fig. 7 [file 44319_2024_125_MOESM15_ESM.zip › Figure 7/Figure 7A/HA-VAPB_emerin_WT_anaphase/HA emd 05_merge.tif]

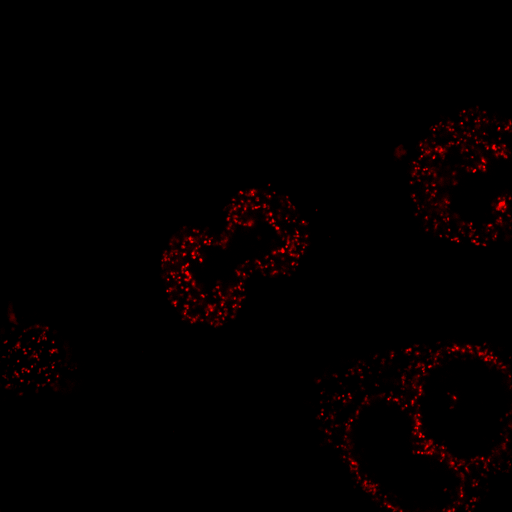

Supplement: Supplementary file 15 — Source data Fig. 7 [file 44319_2024_125_MOESM15_ESM.zip › Figure 7/Figure 7A/HA-VAPB_emerin_WT_anaphase/HA emd 05_PLA.tif]
