# Supplementary material for: Phosphorylation of ELYS promotes its interaction with VAPB at decondensing chromosomes during mitosis
Source: EMBO Rep. 2024 Apr 11;25(5):18. doi: 10.1038/s44319-024-00125-6 (PMC11094025; doi:10.1038/s44319-024-00125-6)
Supplement: Supplementary file 13 — Source data Fig. 5 [file 44319_2024_125_MOESM13_ESM.zip › Figure 5/Figure 5B.pdf]

Figure 5B

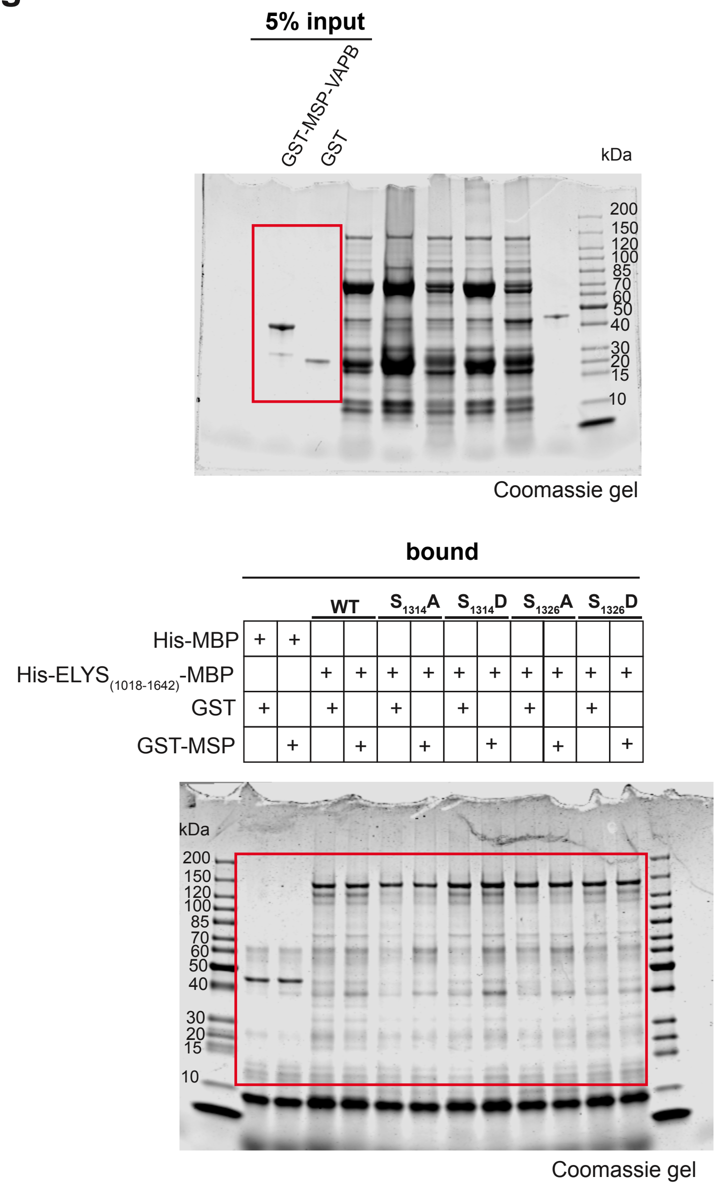

5% input
GST-MSP-VAPB
GST
kDa
200
150
120
100
85
70
60
50
40
30
20
15
10
Coomassie gel
bound
WT
S1314A
S1314D
S1326A
S1326D
His-MBP
His-ELYS(1018-1642)-MBP
GST
GST-MSP
kDa
200
150
120
100
85
70
60
50
40
30
20
15
10
Coomassie gel
