# Supplementary figures and images for: Phosphorylation of ELYS promotes its interaction with VAPB at decondensing chromosomes during mitosis (part 2 of 2)
Source: EMBO Rep. 2024 Apr 11;25(5):18. doi: 10.1038/s44319-024-00125-6 (PMC11094025; doi:10.1038/s44319-024-00125-6)

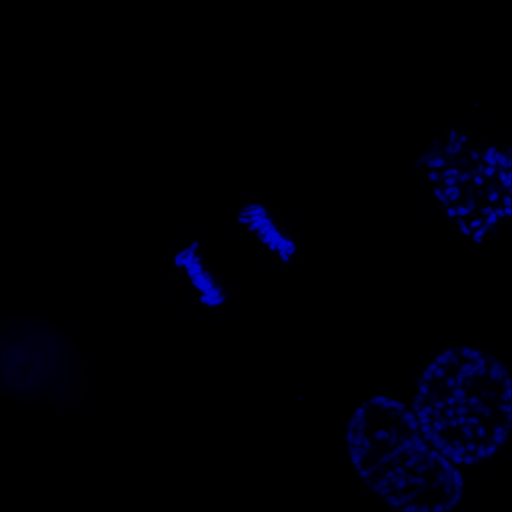

Supplement: Supplementary file 15 — Source data Fig. 7 [file 44319_2024_125_MOESM15_ESM.zip › Figure 7/Figure 7A/HA-VAPB_emerin_WT_anaphase/HA emd 05_DAPI.tif]

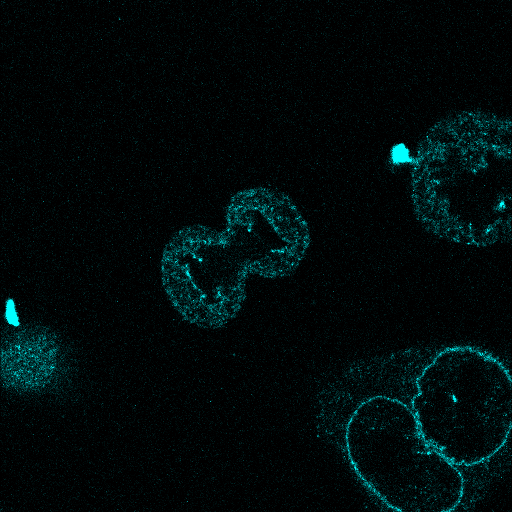

Supplement: Supplementary file 15 — Source data Fig. 7 [file 44319_2024_125_MOESM15_ESM.zip › Figure 7/Figure 7A/HA-VAPB_emerin_WT_anaphase/HA emd 05_emerin.tif]

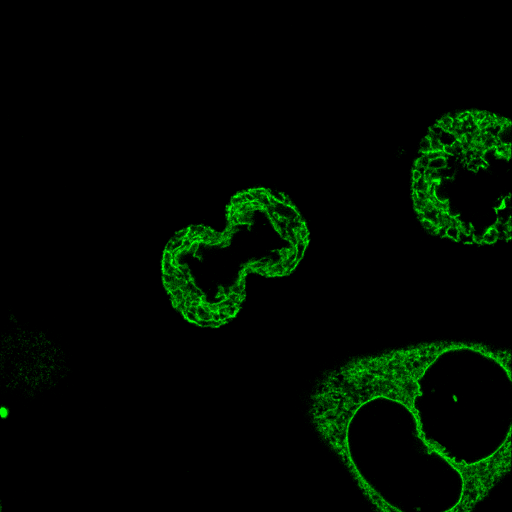

Supplement: Supplementary file 15 — Source data Fig. 7 [file 44319_2024_125_MOESM15_ESM.zip › Figure 7/Figure 7A/HA-VAPB_emerin_WT_anaphase/HA emd 05_HA.tif]

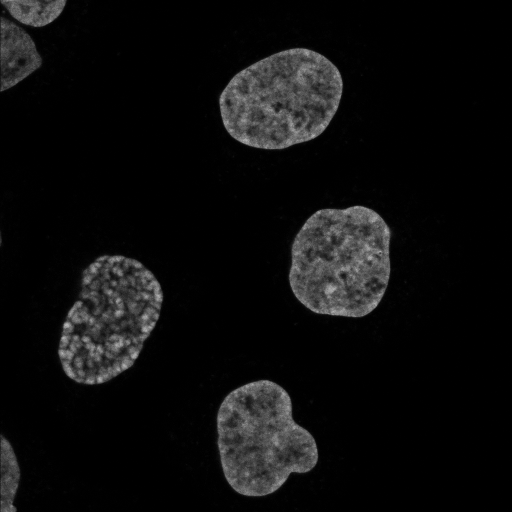

Supplement: Supplementary file 15 — Source data Fig. 7 [file 44319_2024_125_MOESM15_ESM.zip › Figure 7/Figure 7A/HA-VAPB_OSBPL9_KD_MD_interphase cells/HA osbp interphase 02_DAPI.tif]

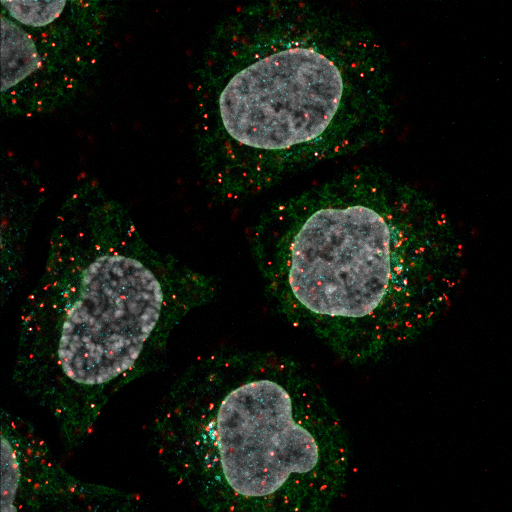

Supplement: Supplementary file 15 — Source data Fig. 7 [file 44319_2024_125_MOESM15_ESM.zip › Figure 7/Figure 7A/HA-VAPB_OSBPL9_KD_MD_interphase cells/HA osbp interphase 02_merge.tif]

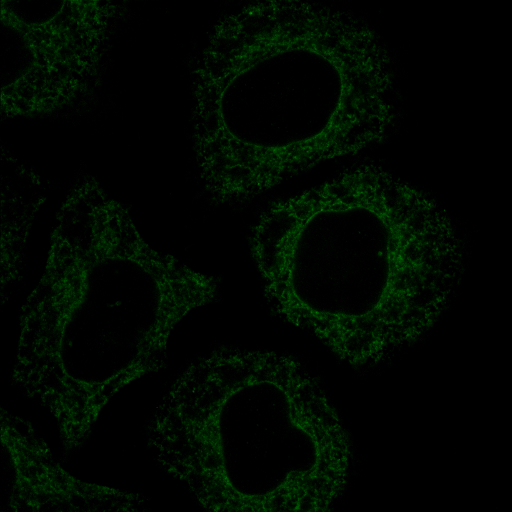

Supplement: Supplementary file 15 — Source data Fig. 7 [file 44319_2024_125_MOESM15_ESM.zip › Figure 7/Figure 7A/HA-VAPB_OSBPL9_KD_MD_interphase cells/HA osbp interphase 02_HA.tif]

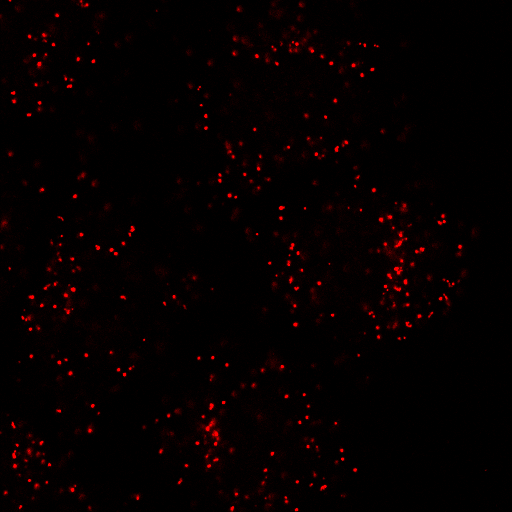

Supplement: Supplementary file 15 — Source data Fig. 7 [file 44319_2024_125_MOESM15_ESM.zip › Figure 7/Figure 7A/HA-VAPB_OSBPL9_KD_MD_interphase cells/HA osbp interphase 02_PLA.tif]

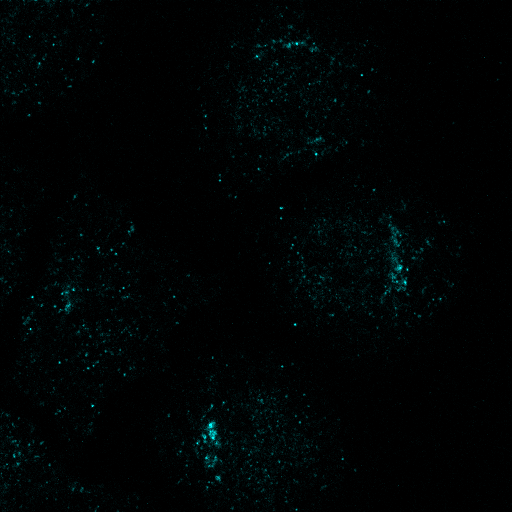

Supplement: Supplementary file 15 — Source data Fig. 7 [file 44319_2024_125_MOESM15_ESM.zip › Figure 7/Figure 7A/HA-VAPB_OSBPL9_KD_MD_interphase cells/HA osbp interphase 02_OSBPL9.tif]

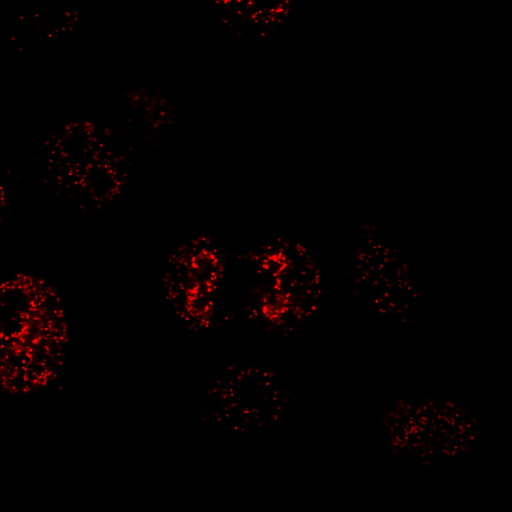

Supplement: Supplementary file 15 — Source data Fig. 7 [file 44319_2024_125_MOESM15_ESM.zip › Figure 7/Figure 7A/HA-VAPB_LBR_WT_anaphase/HA LBR 02_PLA.tif]

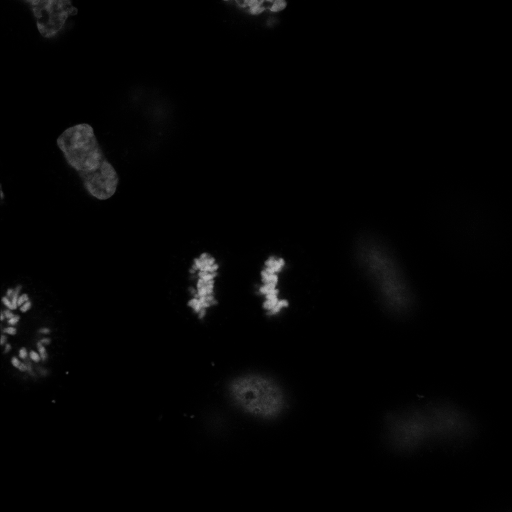

Supplement: Supplementary file 15 — Source data Fig. 7 [file 44319_2024_125_MOESM15_ESM.zip › Figure 7/Figure 7A/HA-VAPB_LBR_WT_anaphase/HA LBR 02_DAPI.tif]

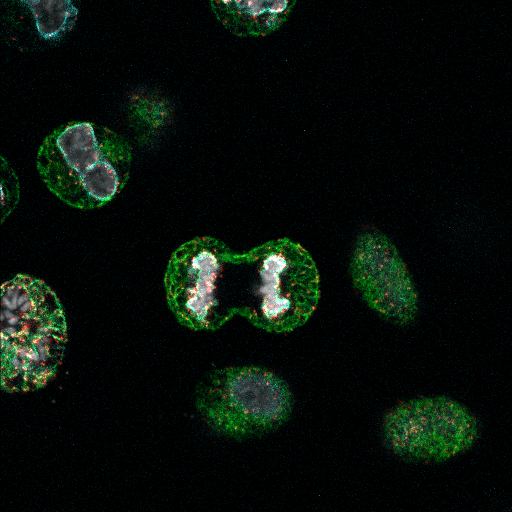

Supplement: Supplementary file 15 — Source data Fig. 7 [file 44319_2024_125_MOESM15_ESM.zip › Figure 7/Figure 7A/HA-VAPB_LBR_WT_anaphase/HA LBR 02_merge.tif]

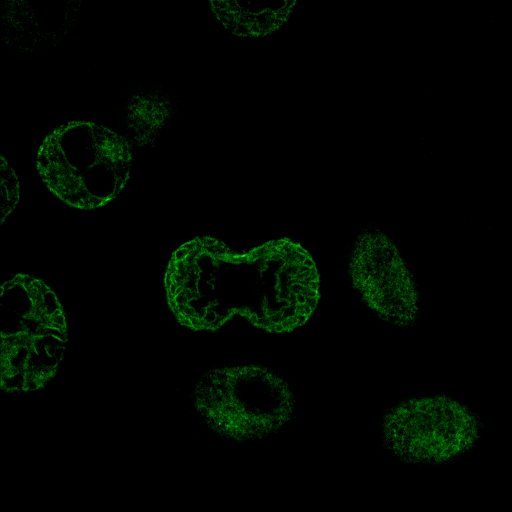

Supplement: Supplementary file 15 — Source data Fig. 7 [file 44319_2024_125_MOESM15_ESM.zip › Figure 7/Figure 7A/HA-VAPB_LBR_WT_anaphase/HA LBR 02_HA.tif]

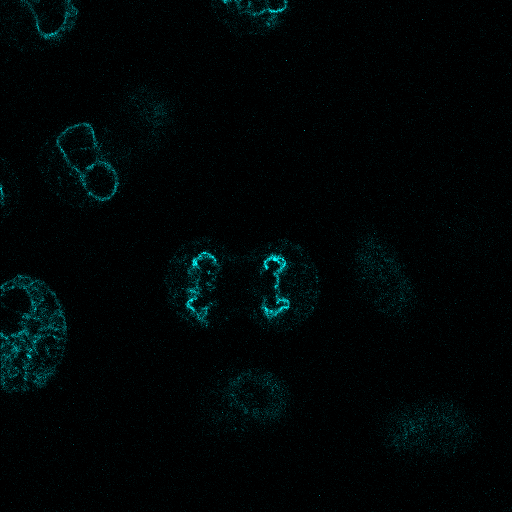

Supplement: Supplementary file 15 — Source data Fig. 7 [file 44319_2024_125_MOESM15_ESM.zip › Figure 7/Figure 7A/HA-VAPB_LBR_WT_anaphase/HA LBR 02_LBR.tif]

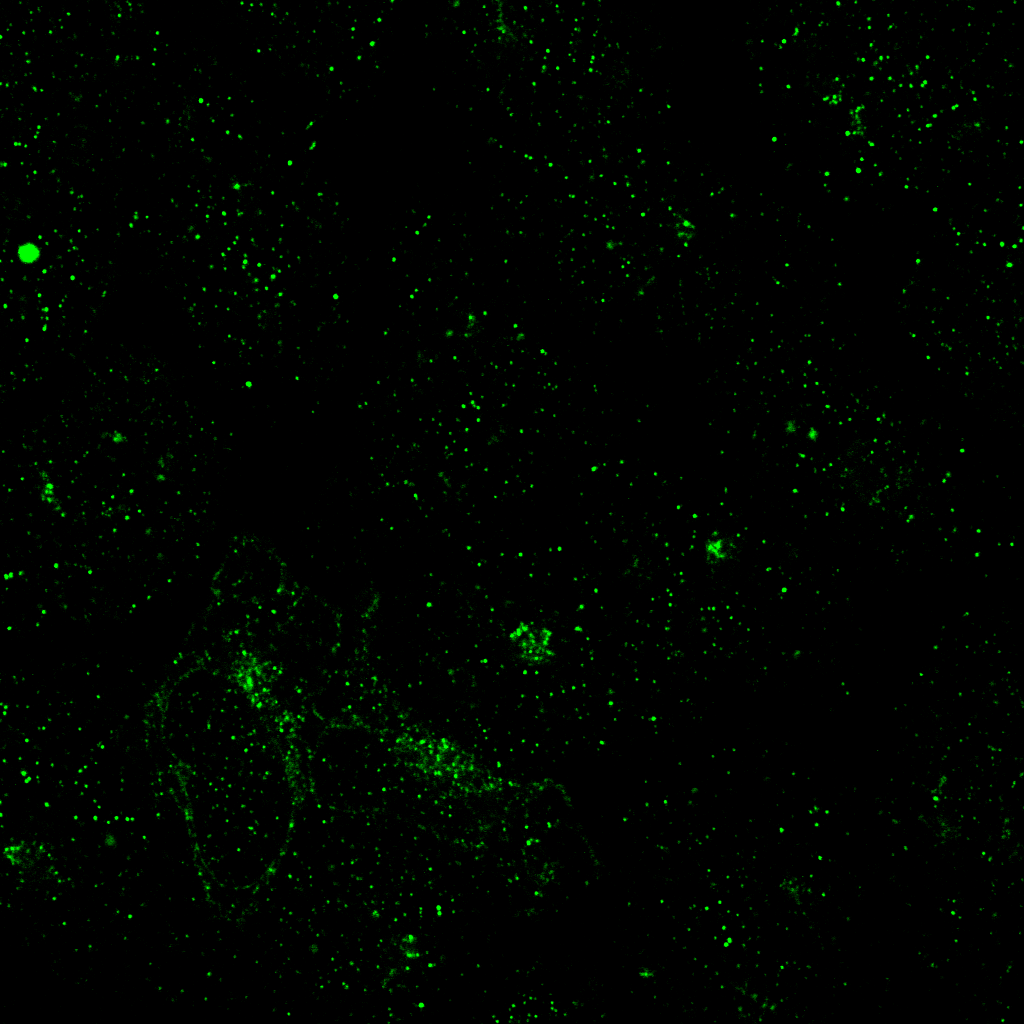

Supplement: Supplementary file 16 — Source data Fig. 8 [file 44319_2024_125_MOESM16_ESM.zip › Figure 8/Figure 8B/siVAPB/sivapb rabbit vapb_VAPB.tif]

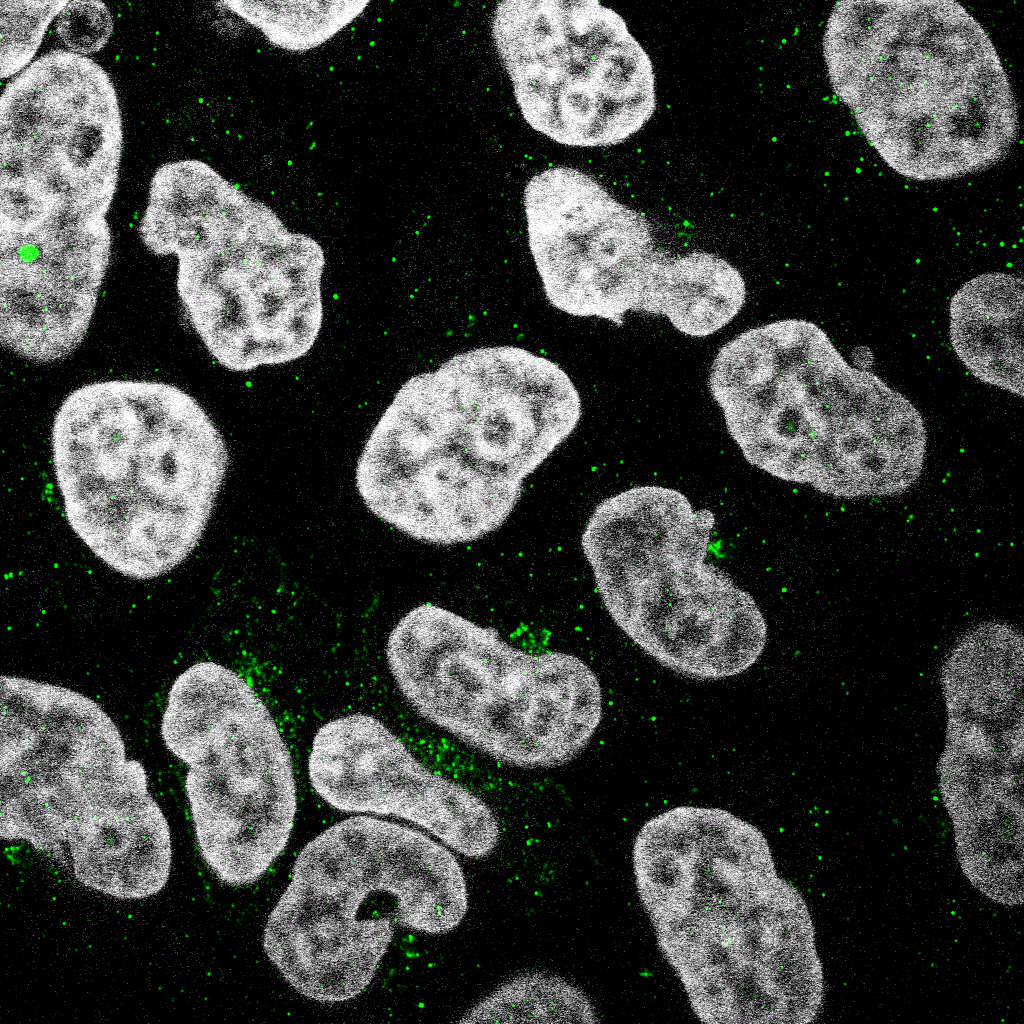

Supplement: Supplementary file 16 — Source data Fig. 8 [file 44319_2024_125_MOESM16_ESM.zip › Figure 8/Figure 8B/siVAPB/sivapb rabbit vapb_merge.tif]

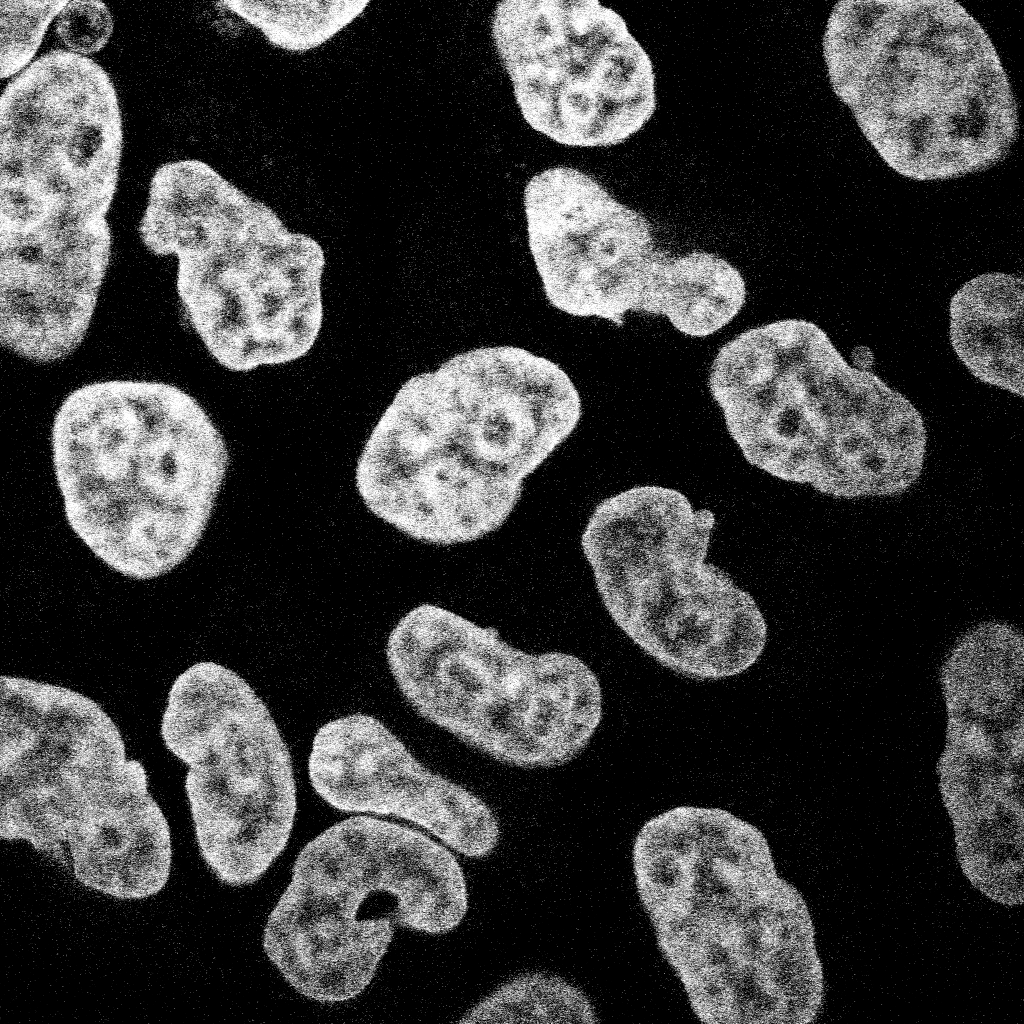

Supplement: Supplementary file 16 — Source data Fig. 8 [file 44319_2024_125_MOESM16_ESM.zip › Figure 8/Figure 8B/siVAPB/sivapb rabbit vapb_DAPI.tif]

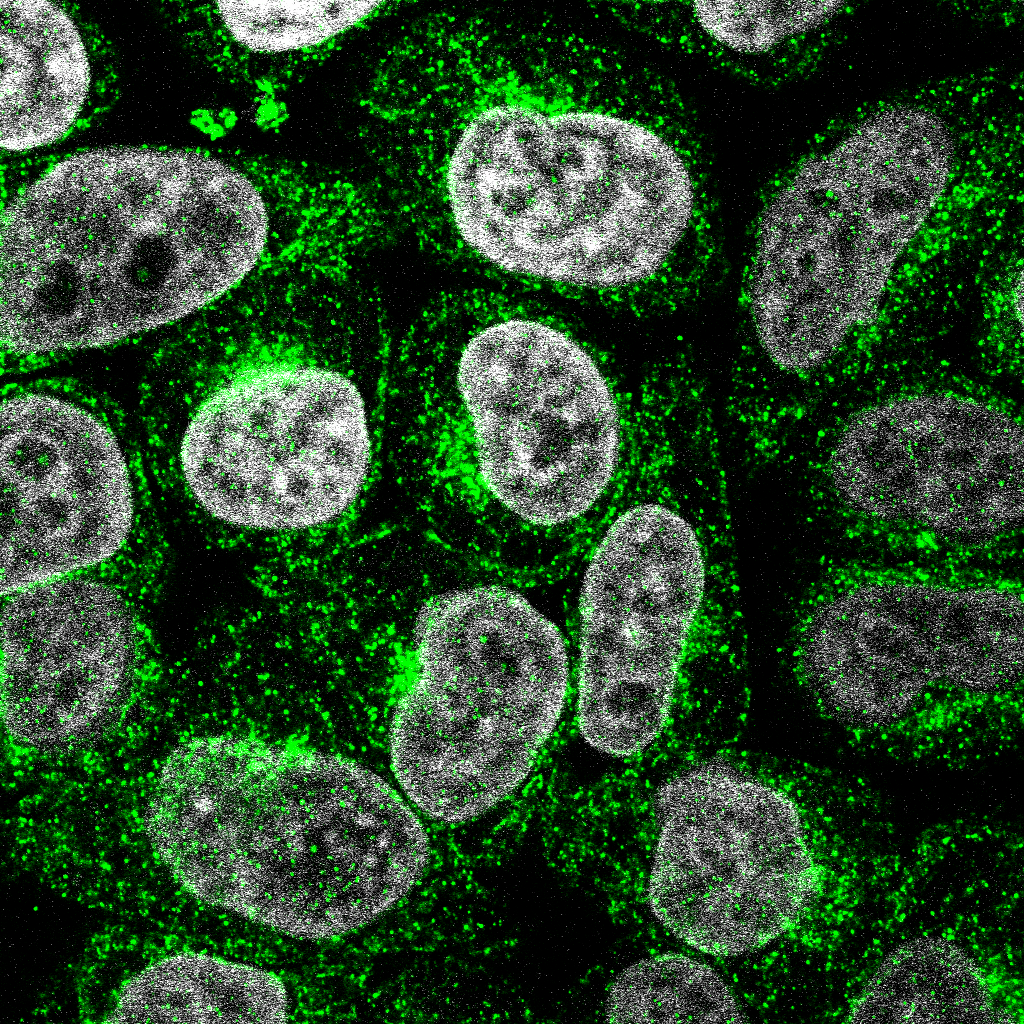

Supplement: Supplementary file 16 — Source data Fig. 8 [file 44319_2024_125_MOESM16_ESM.zip › Figure 8/Figure 8B/sint/si nt_rabbit vapb fig8_merge.tif]

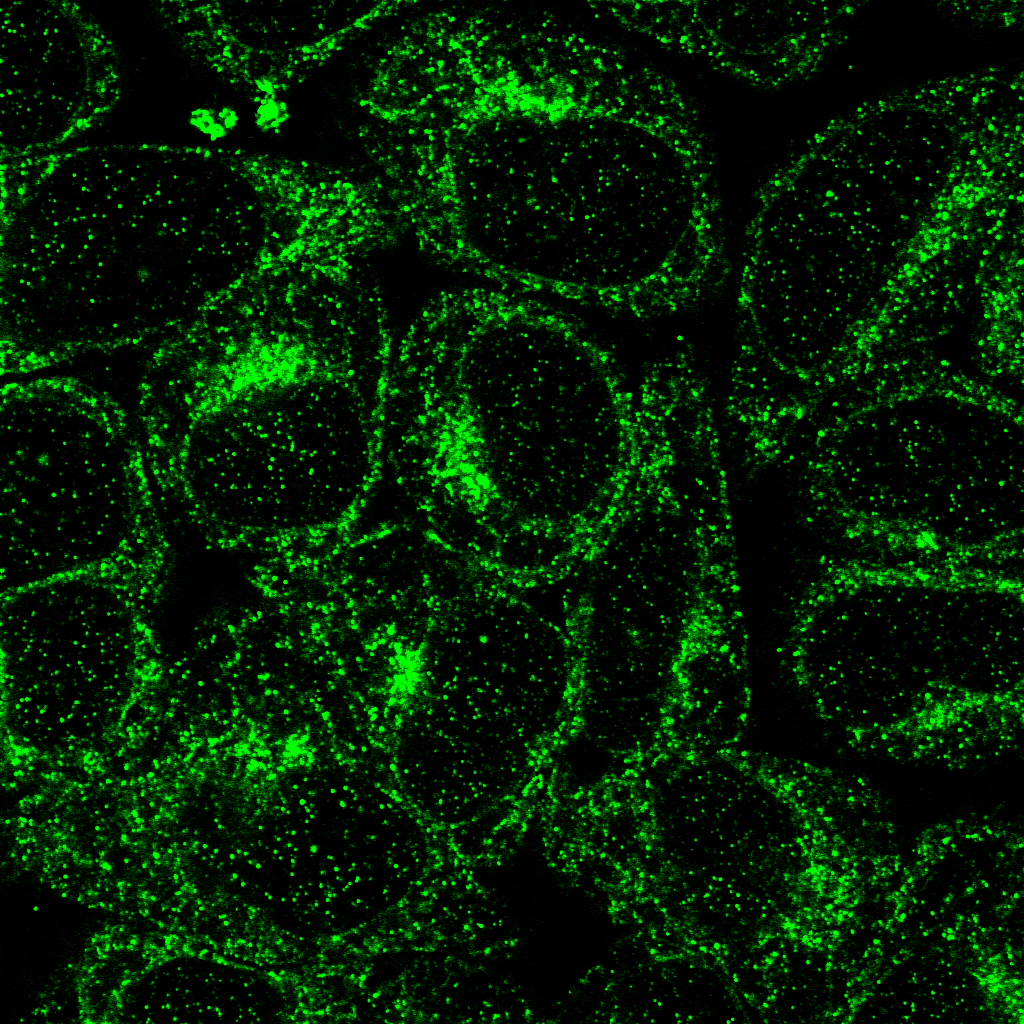

Supplement: Supplementary file 16 — Source data Fig. 8 [file 44319_2024_125_MOESM16_ESM.zip › Figure 8/Figure 8B/sint/si nt_rabbit vapb fig8_VAPB.tif]

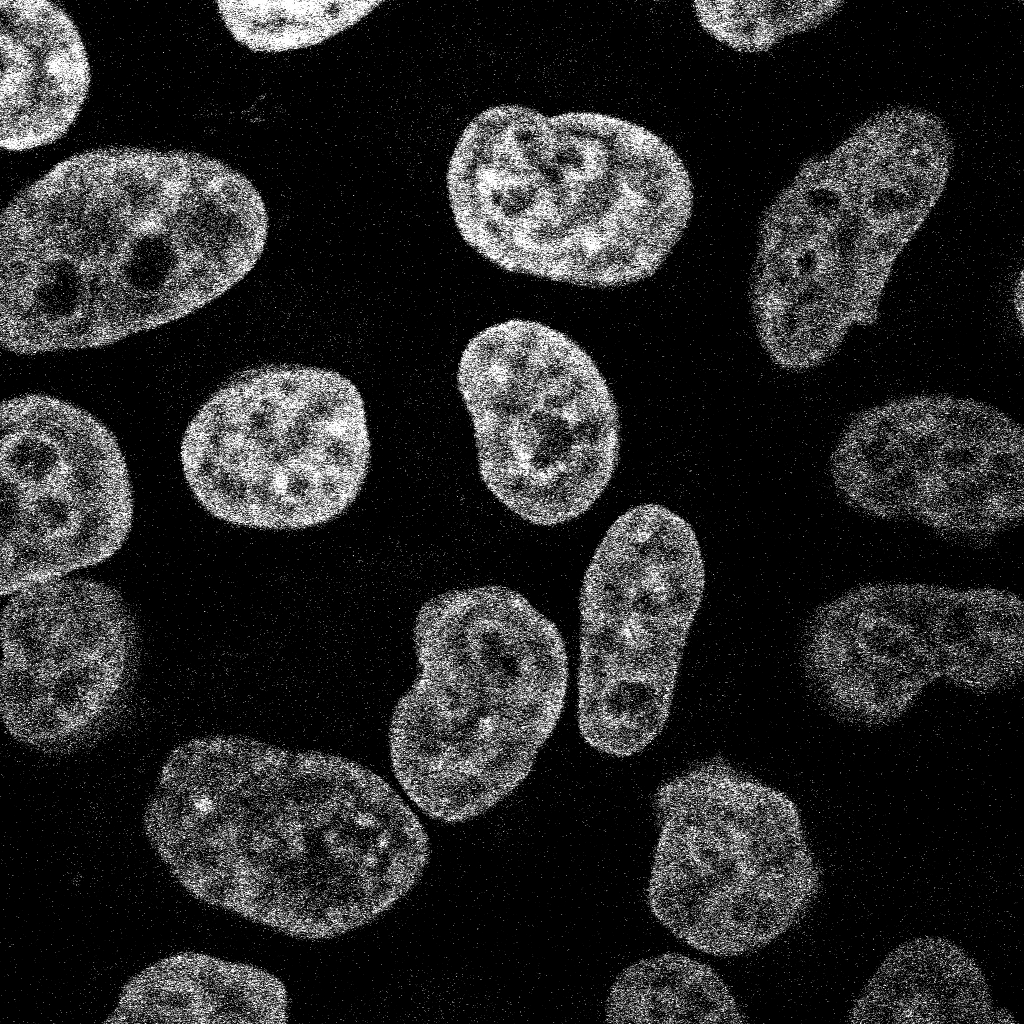

Supplement: Supplementary file 16 — Source data Fig. 8 [file 44319_2024_125_MOESM16_ESM.zip › Figure 8/Figure 8B/sint/si nt_rabbit vapb fig8_DAPI.tif]
