# Supplementary material for: Phosphorylation of ELYS promotes its interaction with VAPB at decondensing chromosomes during mitosis
Source: EMBO Rep. 2024 Apr 11;25(5):18. doi: 10.1038/s44319-024-00125-6 (PMC11094025; doi:10.1038/s44319-024-00125-6)
Supplement: Supplementary file 18 — Expanded View Figures [file 44319_2024_125_MOESM18_ESM.pdf]

Expanded View Figures

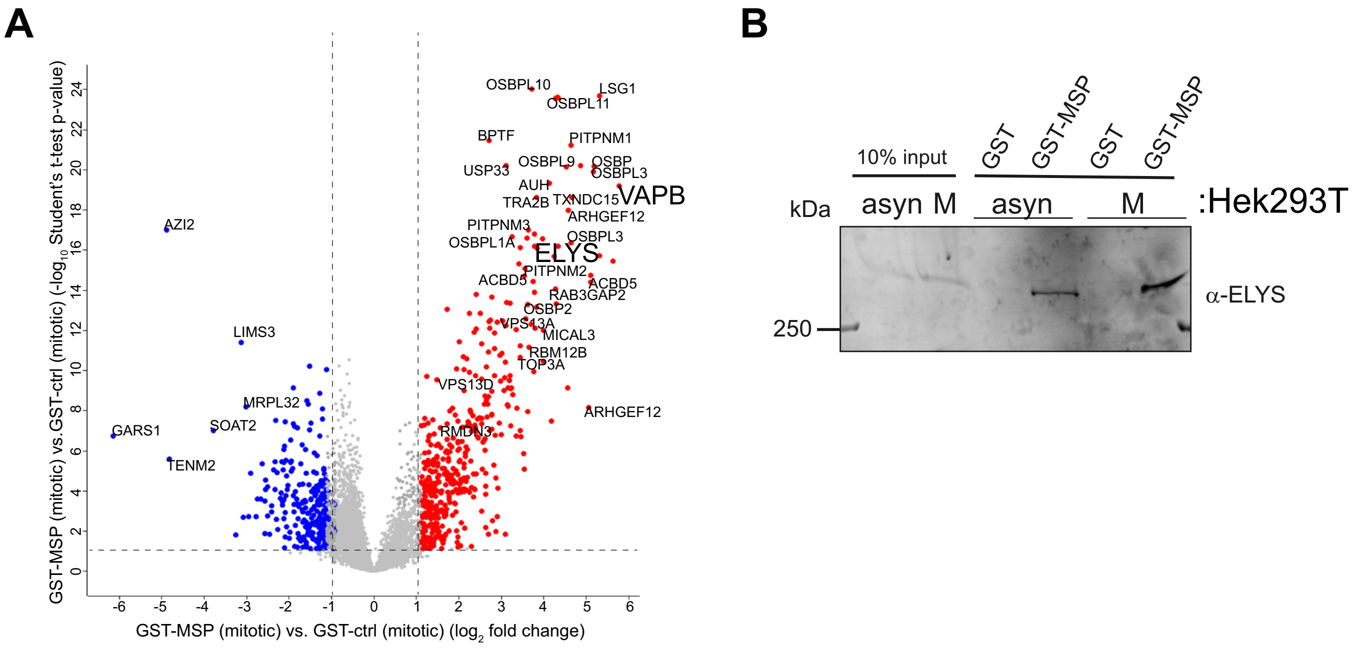

Figure EV1. The MSP-domain of VAPB interacts with ELYS in mitosis.

(**A**) MS-DIA-results comparing proteins from mitotic cells bound to GST or GST-MSP. Proteins enriched on GST-MSP beads are depicted in red. The graph shows the combined results of four independent experiments with two technical replicates each. A two-sided Student's T-test was performed using normalized $\log_2$ ratios. For the analysis, a Permutation-based FDR was applied, and a threshold value of 0.05 was chosen. (**B**) HEK293T-cells were subjected to a synchronization protocol and lysates from asynchronous (asyn) and mitotic (M) cells were obtained. Lysates were then incubated with immobilized GST or GST-MSP and bound proteins were analyzed by SDS-PAGE followed by Western blotting detecting ELYS. Compare Fig. 1E.

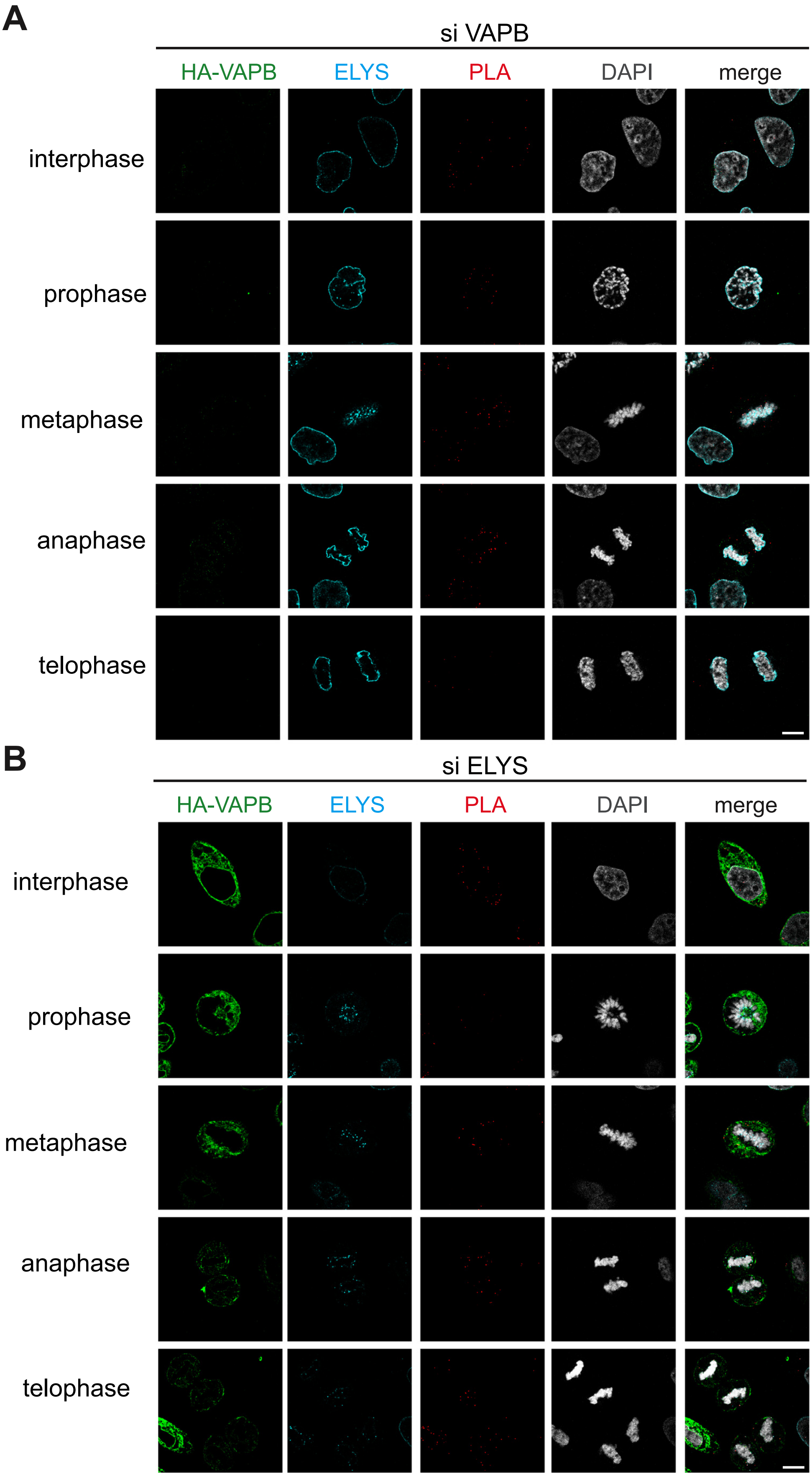
A
si VAPB
HA-VAPB
ELYS
PLA
DAPI
merge
interphase
prophase
metaphase
anaphase
telophase
B
si ELYS
HA-VAPB
ELYS
PLA
DAPI
merge
interphase
prophase
metaphase
anaphase
telophase

 © The Author(s)

Figure EV2. Specificity of VAPB-ELYS interactions in anaphase shown by knockdown of either VAPB or ELYS.

HeLa T-REx Flp-In cells stably expressing HA-VAPB were treated with siRNAs against VAPB (**A**) or ELYS (**B**) and synchronized and released as described in Fig. 6A and subjected to PLAs. Indirect immunofluorescence was performed to detect HA-VAPB and ELYS. Scale bar, 10 µm.

© The Author(s)

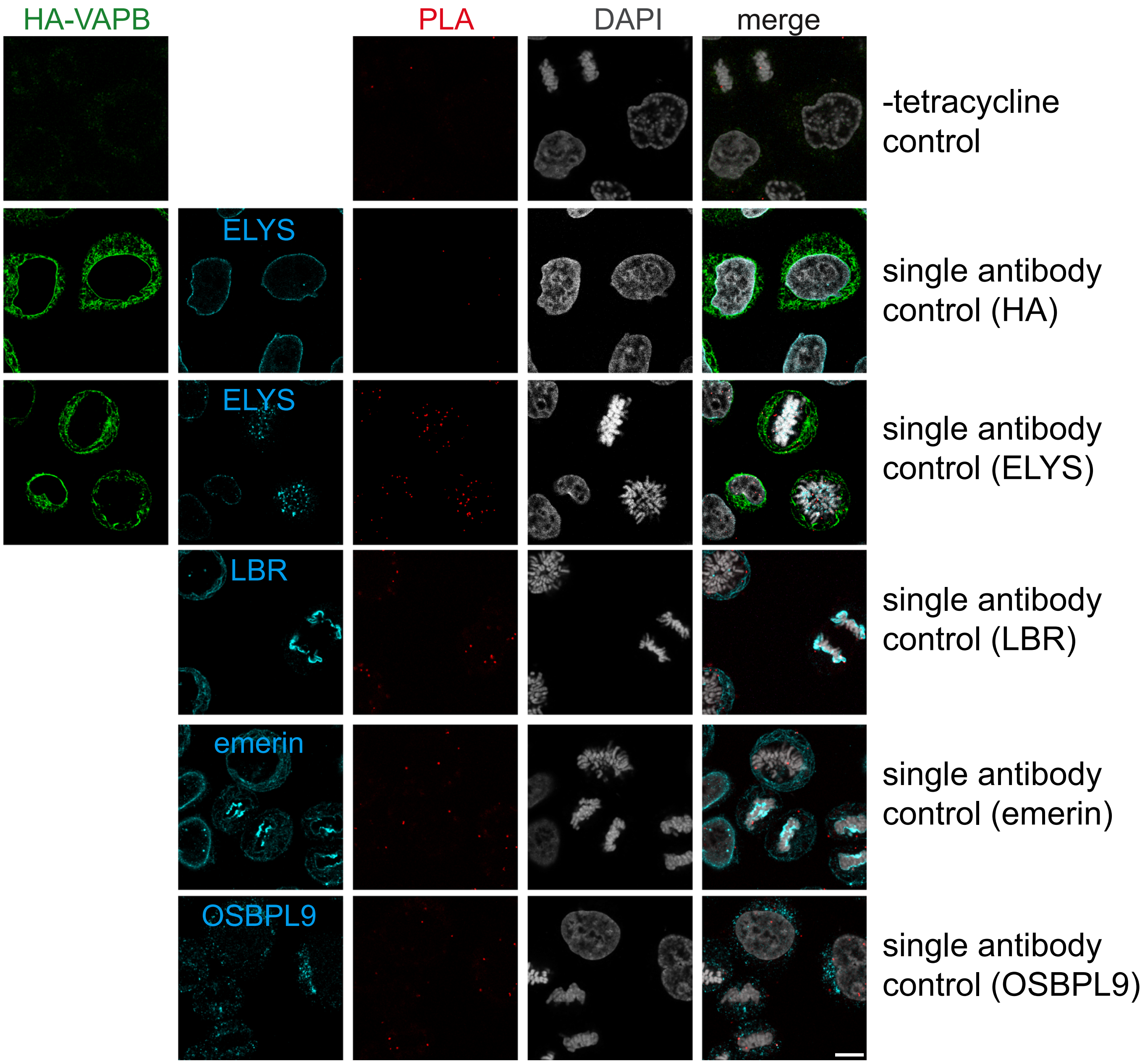

Figure EV3. Single antibody controls using antibodies against HA, ELYS, LBR, emerin, and OSBPL9 to determine the specificity of PLA interactions. Scale bar, 10 µm.

 © The Author(s)

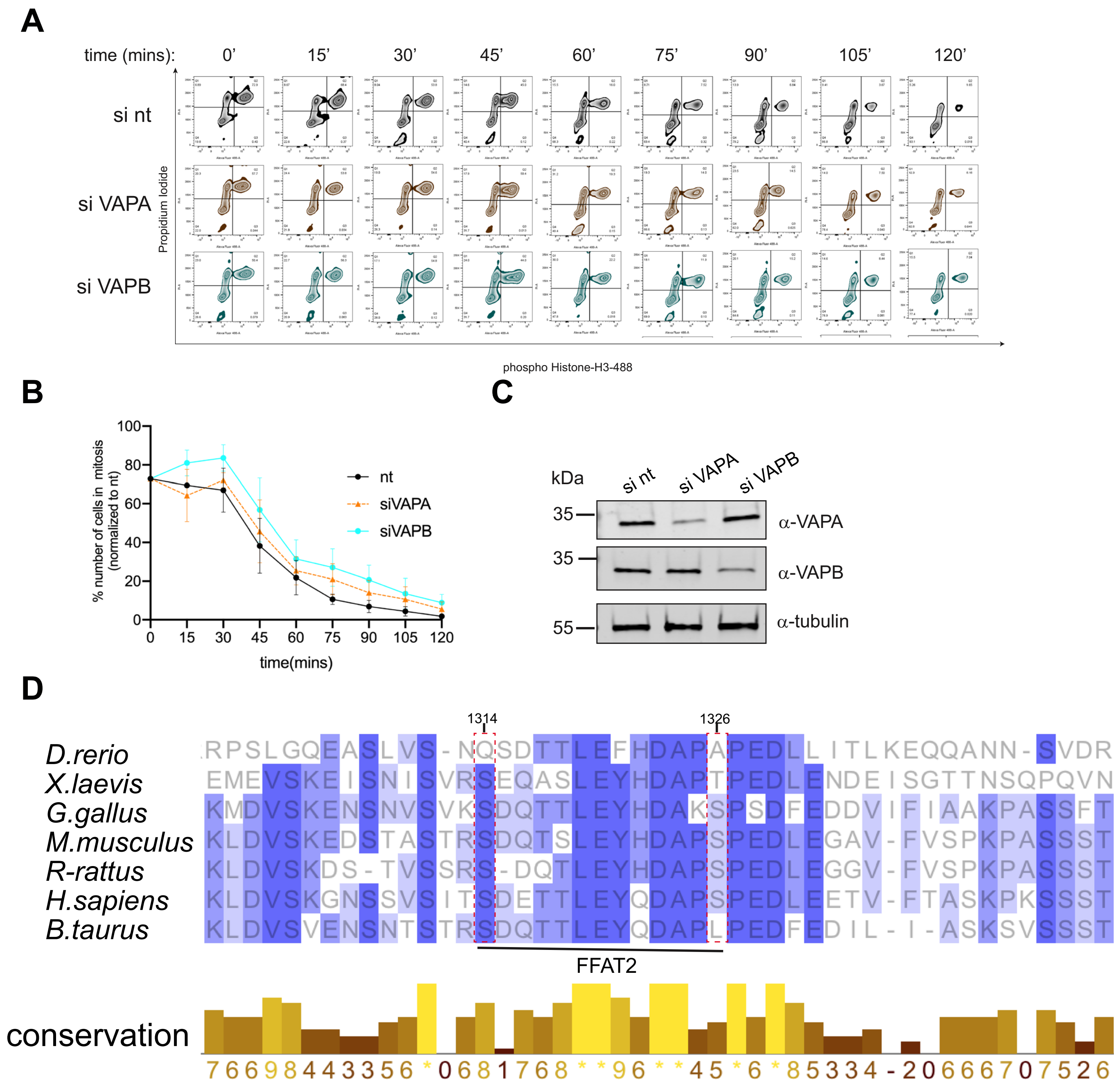

Figure EV4. Effects of knockdown of VAPA and VAPB in mitosis.

(**A**) HeLa P4 cells were transfected with siRNAs against VAPA (siVAPA) or VAPB (siVAPB) or non-targeting siRNAs (si nt) and synchronized and further treated as described in the legend to Fig. 8C. Cells were then analyzed by flow cytometry using propidium iodide and antibodies against phospho-Histone H3. Mitotic cells are observed in the upper right quadrant. (**B**) Quantification of the results in (**B**), indicating the percentage of mitotic cells as determined by flow cytometry. The data are shown as the mean (± standard deviation) of three biological replicates. (**C**) HeLa P4 cells subjected to knock down of VAPA or VAPB as indicated in (**A**) were analyzed by SDS-PAGE and Western blotting using antibodies against VAPA, VAPB, and tubulin. (**D**) Mutiple sequence alignment (MSA) of the FFAT2-motif of ELYS from *Danio rerio, Xenopus laevis, Gallus gallus, Mus musculus, Rattus rattus, Homo sapiens, Bos taurus*. Conservation of the amino acid residues are represented from 0 to 9 and *. Phosphorylation sites S_{1314} and S_{1326} are indicated by dotted red boxes. Jalview software (Waterhouse et al, 2009) was used to perform MSA.

© The Author(s)
